# Structure and function of a fungal AB toxin-like chimerolectin involved in anti-nematode defense

**DOI:** 10.1038/s44318-026-00812-1

**Published:** 2026-05-26

**Authors:** Stefanie S Schmieder, Gabriele Cordara, Flore Kersten, Kevin Steiner, Clara H Samim, David F Plaza, Ahmad Ali-Ahmad, Andreas Boeggild, Jesper L Karlsen, Blanka O Sokolowska, Thomas Boesen, Ute Krengel, Markus Künzler

**Affiliations:** 1 Institute of Microbiology, Department of Biology, ETH Zürich, Zürich, Switzerland; 2 Department of Chemistry, University of Oslo, Oslo, Norway; 3 Centre for Molecular Medicine Norway, University of Oslo, Oslo, Norway; 4 Department of Molecular Biology and Genetics, Aarhus University, Aarhus, Denmark; 5 Present Address: Division of Gastroenterology, Boston Children’s Hospital, Harvard Medical School, Boston, MA USA; 6 Present Address: Institute of Medical Virology, University of Zürich, Zürich, Switzerland

**Keywords:** Digestive System, Microbiology, Virology & Host Pathogen Interaction, Structural Biology

## Abstract

Fungal defense against predators largely relies on protein toxins, many of which are lectins. We previously showed that the production of the nematotoxin CCTX2 is upregulated in the Agaricomycete *Coprinopsis cinerea* upon predation by nematodes. Here, we classify CCTX2 as the founding member of a previously unknown family of fungal chimerolectins. Cryo-EM analysis to 3.2 Å resolution reveals five domains, with the four N-terminal β-trefoil fold (BTF) domains cradling a C-terminal domain, which exhibits an unusual α + β protein fold with some similarity to killer protein 4. Mutational analysis suggests that both terminal domains are functionally required for nematotoxicity. The first two BTF domains enable CCTX2 binding to glycosphingolipids with LacNAc or LacdiNAc glycoepitopes on nematode intestinal epithelial cells, whereas the biochemical function of the C-terminal domain remains unknown. Experiments in the model nematode *Caenorhabditis elegans* demonstrate that CCTX2 exploits the endocytic and retrograde trafficking machinery of cells in the intestinal epithelium to exert its toxicity and access the yet-to-be-identified intracellular target of the non-lectin domain. Our findings thus show that the molecular architecture and mode of action of CCTX2 is reminiscent of bacterial and plant AB toxins.

## Synopsis

Mushrooms defend themselves against fungivorous nematodes by producing cytoplasmic nematotoxins. This work identifies a novel family of fungal nematotoxins, which includes paralogues CCTX1-3 from the agaricomycete *Coprinopsis cinerea*, and displays structural and functional similarity to bacterial and plant AB toxins.

- The cryo-EM structure of CCTX2 reveals a new fold for the C-terminal domain of unknown function (DUF), which is cradled on four N-terminal β-trefoil domains BTF1-4.
- BTF1-2 and DUF domains are required for CCTX2 toxicity towards the model nematode *Caenorhabditis elegans*.
- CCTX2 binds via BTF1-2 to LacNAc and LacdiNAc-containing glycosphingolipids on *C. elegans* intestinal epithelial cells.
- Upon receptor binding, CCTX2 is endocytosed and undergoes retrograde trafficking to elicit toxicity.

## Abstract (web summary)

Identification and characterization of a novel family of mushroom nematotoxins with structural and functional resemblance to bacterial and plant AB toxins.

## Graphical abstract

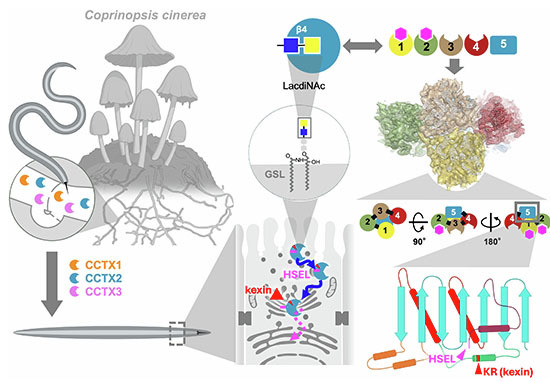

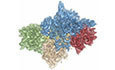

## Introduction

Protein toxins are involved in many inter-organismal conflicts and are especially prevalent in plants and bacteria (Aravind et al, 2012; Zhang et al, 2012). AB toxins are one type of such defense toxins and are formed by two protein components: the A subunit (Active subunit), responsible for exerting toxicity inside the cell, and B subunits (Binding subunits), responsible for cell binding and entry (Song, 2022). Ricin (plants), Shiga and cholera toxin (bacteria) are considered prime examples of this class of toxins (Sánchez and Holmgren, 2011; Melton-Celsa, 2014; Heggelund et al, 2015; Polito et al, 2019). AB toxins can be single-chain or multiprotein toxins (Song, 2022) and are found in nature with varying subunit stoichiometries, ranging from AB (e.g., ricin and diphtheria toxin) and AB_2_ (e.g., cytolethal distending toxin) to AB_5_ (e.g., Shiga, cholera, and pertussis toxins) or A_2_B_5_ (e.g., typhoid toxin) (Song et al, 2013a; Alouf et al, 2015; Brown et al, 2023; Popoff, 2024).

The B subunit often recognizes a glycoepitope with high specificity, thus classifying it as a sugar-binding protein, or lectin. This subunit mediates the binding of the AB toxin to a glycoconjugate on the target cell surface and triggers the entry of the toxin into the target cell by endocytosis (Falnes and Sandvig, 2000; Mancheño et al, 2005; Manna et al, 2017), followed by retrograde transport to the endoplasmic reticulum (ER), disassembly and delivery to its final compartment, which is often the cytosol. Once inside, the A subunit exerts its toxicity by binding to and/or modifying one or more intracellular targets (Teter, 2013). Catalytic activities of AB toxins include ADP-ribosyltransferase activity (cholera, pertussis and diphtheria toxins) (Simon et al, 2014), rRNA *N*-glycosidase activity (ricin and Shiga toxin) (Melton-Celsa, 2014; Polito et al, 2019), proteolysis (botulinum toxin, tetanus toxin, Anthrax lethal factor) (Blasi et al, 1993; Montecucco and Schiavo, 1995; Duesbery et al, 1998) and DNA cleavage (cytolethal distending toxin, typhoid toxin) (Pons et al, 2019)). In many cases, the protein toxins need to be activated in the host e.g., by proteolytic processing (e.g., diphtheria, cholera, Shiga and anthrax toxins, and *Pseudomonas* exotoxin A) and/or the reduction of S-S bonds (e.g., diphtheria, cholera, Shiga, pertussis and typhoid toxins, and *Pseudomonas* exotoxin A) (Gordon and Leppla, 1994; Odumosu et al, 2010; Song et al, 2013a; Alouf et al, 2015). An important family of host proteases exploited for bacterial toxin (and viral protein) activation are furins which play an important role in the maturation of endogenous proteins and peptides in the secretory system (Thomas, 2002; Rockwell and Thorner, 2004). Less is known for fungal protein toxins, but similar activation mechanisms exist. For example, activation of Candidalysin, which is an example of pore-forming toxins, occurs through proteolytic processing by kexins (fungal furin orthologues) as part of its maturation in the fungus *Candida albicans* (Richardson et al, 2018).

The sessile lifestyle and the high concentration of macronutrients render fungi an attractive food source for the soil-grazing fauna. To protect themselves from being foraged by, e.g., nematodes, fungi employ a combination of secondary metabolites and a protein-based defense system; the latter includes several lectins (Singh et al, 2010; Bleuler-Martínez et al, 2011; Kempken, 2011; Varrot et al, 2013; Sommer et al, 2018). Fungivorous nematodes pierce fungal cells and aspirate their cytoplasmic content; consistently, defense lectins are cytoplasmically localized (Künzler, 2015). After ingestion, the lectin travels through the digestive tract of nematodes to eventually bind to a cell surface receptor and exert its toxic effect (Künzler, 2015; Cordara et al, 2018).

Lectins include mero- (monomeric) and hololectins (multimeric), exclusively formed by sugar-binding domains, and chimerolectins, where lectin and non-lectin domains coexist or assemble in the same protein (Peumans and Van Damme, 1995; Peumans et al, 2001). Some chimerolectins are functionally analogous to AB toxins. Studies of the fungal chimerolectin *Marasmius oreades* agglutinin (MOA) suggest that the lectin domain acts as a B subunit and the non-lectin domain as an A subunit (Cordara et al, 2011; Wohlschlager et al, 2011; Cordara et al, 2016). The toxicity mechanism of MOA includes binding to glycosphingolipids exposed on the surface of the target cell as well as endocytosis, retrograde trafficking, and (putative) modification of an intracellular target (Cordara et al, 2011; Wohlschlager et al, 2011; Cordara et al, 2014; Künzler, 2015; Juillot et al, 2016). MOA exerts strong toxicity against nematodes, including the bacterivorous model organism *Caenorhabditis elegans*, and is thought to be part of the chemical defense of the host *M. oreades* against these micropredators (Wohlschlager et al, 2011; Tayyrov et al, 2018a).

*Coprinopsis cinerea* toxin 2 (CCTX2) was previously identified as a nematotoxic defense protein in the Agaricomycete *Coprinopsis cinerea*, based on the induced expression of the *cctx2* gene in the vegetative mycelium of the fungus in the presence of fungivorous nematodes (Plaza et al, 2016). In the present article, we performed a structure/function analysis of the CCTX2 protein toxin. Within the *C. cinerea* genome, we additionally identified the genes of two CCTX2 paralogs, which we termed *cctx1* and *cctx3*. The three genes differ in their expression pattern regarding sexual development and bacterial or nematode challenge; however, we demonstrate that the encoded proteins all exert strong nematotoxicity and show the same domain partition: four N-terminal β-trefoil fold (BTF) domains followed by a C-terminal domain of unknown function (DUF). The structure of CCTX2, determined to 3.2 Å resolution by single-particle cryo–electron microscopy (cryo-EM), revealed that the BTF domains form a cradle carrying the C-terminal domain. The C-terminal domain adopts an unusual α + β protein fold. Deletion of the first two BTF domains of CCTX2 leads to the loss of binding of the chimerolectin to LacNAc and LacdiNAc glycoepitopes. This, together with the presence of experimental density at the carbohydrate-binding sites in the two N-terminal BTF domains, suggests that the nematotoxicity of CCTX2 depends on the binding of the protein to LacdiNAc-containing glycosphingolipids (GSLs) on the surface of nematode intestinal epithelial cells. Upon binding, CCTX2 undergoes endocytosis and retrograde trafficking of the protein-GSL complexes in these cells. The nematotoxicity of CCTX2 also depends on the presence and integrity of the C-terminal, non-lectin domain. The intracellular target and the biochemical function of this domain remain unknown. Based on orthologs encoded in the genomes of other Agaricomycetes and some ascomycetes, we conclude that CCTX2 is the founding member of a family of fungal defense effectors against fungivorous nematodes.

## Results

### CCTX2 belongs to a family of fungal nematotoxins

**Figure 1:**
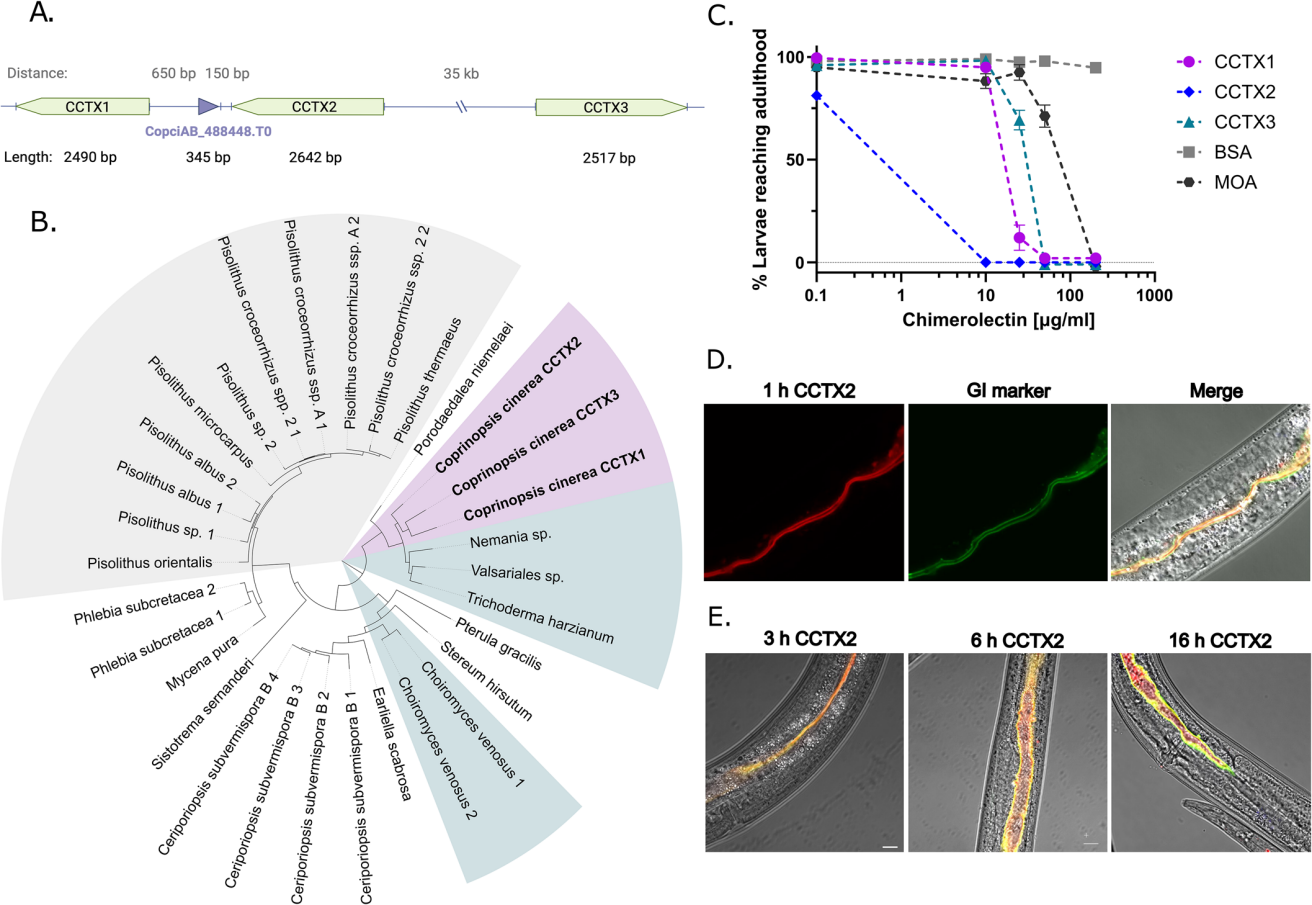
The nematotoxin CCTX2 is a member of a large family of fungal chimerolectins. (**A**) Location of CCTX paralogs in the *C. cinerea* genome (*Coprinopsis cinerea* AmutBmut v.2 annotation from JGI). (**B**) Phylogenetic analysis of full-length homologs of CCTX2. Paralogues in *C. cinerea* are highlighted in pink, homologs in *Pisolithus* spp. in gray, and homologs in Ascomycetes in turquoise. See Appendix Table S1 for the JGI protein IDs of all displayed homologs and Appendix Fig. S1A for a sequence alignment of homologs from the monophyletic clade, including CCTX1-3. (**C**) Nematotoxicity of the paralogous CCTX1, CCTX2, and CCTX3, measured by assessing the percentage of L1 larvae reaching adulthood. Development of *C. elegans* N2 larvae was arrested at the L1 stage in the presence of either of the three proteins at concentrations >10 μg/ml. This toxicity is higher than that of the MOA chimerolectin, which was included as a positive control. Bovine serum albumin (BSA) served as a negative control. Data points with error bars indicate means of *N* = 4 biological replicates with standard error of the mean (SEM). Overlapping data points were nudged by +/− 2 data units in *y* for better readability. For the same reason, the data points were connected despite the fact that no data points in between were determined. (**D**, **E**) Localization of CCTX2-TAMRA upon feeding to L4 larvae of *C. elegans* strain GK70. This strain expresses PGP-1-GFP as an intestinal apical membrane marker. Images in panel D are of the same worm and were taken 1 h after feeding with CCTX2-TAMRA. (**E**) Images in (**E**) show a time course of intoxification by CCTX2. After 3 h, feeding of CCTX2-TAMRA led to aberrant dilatation of the intestinal lumen. Images were taken at the rear end of different worms representative of the population. Scale bar = 10 µm. Source data are available online for this figure.

A query on the *Coprinopsis cinerea* genome (JGI Mycocosm *Coprinopsis cinerea* AmutBmut v2) using the *cctx2* gene sequence (gene model ID CopciAB_369589.T0) revealed the existence of two paralogues, aptly designated as *cctx1* and *cctx3* (gene model IDs CopciAB_369594.T0 and CopciAB_369576.T0, respectively), which are located on the same chromosome in close neighborhood of the *cctx2* gene (Fig. 1A). According to published data (Data ref: Muraguchi et al, 2015; Tayyrov et al, 2018b; Kombrink et al, 2019b) (Appendix Fig. S1A,B), the three paralogues differ significantly in their expression patterns: *cctx2* is expressed at low levels in the vegetative mycelium and is induced upon predation by the fungivorous nematode *Aphelenchus avenae* but not upon bacterial challenge. Expression of *cctx1* is also low in the vegetative mycelium, but the gene is strongly upregulated upon nematode challenge and in stage 1 primordia during sexual development, whereas *cctx3* is strongly expressed in the vegetative mycelium and downregulated during sexual development as well as upon nematode and bacterial challenge. A BLAST search using the full-length CCTX1, CCTX2, and CCTX3 sequences (JGI Mycocosm *Coprinopsis cinerea* AmutBmut v2 protein IDs 2257338, 2257340, and 2257352, respectively) revealed the existence of several CCTX homologs (Tayyrov et al, 2018a; Kombrink et al, 2019a) over the full length of the protein, specific to the fungal kingdom (Fig. 1B; Appendix Fig. S1D and Table S1). Most homologs were detected in Agaricomycetes, with a remarkable expansion in the genus *Pisolithus*. In addition, a few homologs were encoded by Ascomycetes.

The specific induction of *cctx1* and *cctx2* by *A. avenae* suggests a function of these genes in the defense of *C. cinerea* against fungivorous nematodes. Indeed, we previously showed that CCTX2 is toxic toward the nematode model species, *Caenorhabditis elegans* (Plaza et al, 2016). To test if all three paralogous genes encode defense effector proteins, we produced the proteins in *E. coli* and tested their toxicity against *C. elegans*. All three paralogous proteins inhibited the development of *C. elegans* L1 larvae at low concentrations (< 50 µg/ml, Fig. 1C). The inhibition of larval development was comparable to the effects of the *Marasmius oreades* chimerolectin MOA and much stronger than for the *C. cinerea* hololectin CCL2 (*C. cinerea* lectin 2) (Appendix Fig. S2) (Wohlschlager et al, 2011). Feeding of TAMRA-labeled CCTX2 protein to L4-staged *C. elegans* GK70 larvae (harboring GFP-labeled apical plasma membrane marker protein PGP-1) revealed co-localization of the chimerolectin CCTX2 and PGP-1 with the intestinal epithelium (Fig. 1D). Accordingly, exposure to CCTX2 resulted in clear morphological changes of the intestine, such as gross distension of the intestinal lumen and an increasingly undulating phenotype of the apical plasma membrane after more than 2 h of feeding (Fig. 1E). These morphological changes increased in severity over time, leading to death of the worms around 16–24 h after toxin administration. Similar morphological changes to the intestinal phenotypes were previously described for other nematotoxic fungal lectins, such as the chimerolectin MOA and the two *C. cinerea* hololectins CGL2 (*C. cinerea* galectin 2) and CCL2 (Butschi et al, 2010; Wohlschlager et al, 2011; Schubert et al, 2012; Stutz et al, 2015). Besides *C. elegans*, we found that CCTX2 was also toxic to the larvae of two other *Caenorhabditis* species (Appendix Fig. S1C). A similarly broad specificity as CCTX2 was also observed for the chimerolectin MOA but not for the hololectin CGL2 (Appendix Fig. S1C).

### CCTX2 fold comprises four β-trefoil (BTF) domains and a C-terminal domain of unknown function (DUF)

**Figure 2:**
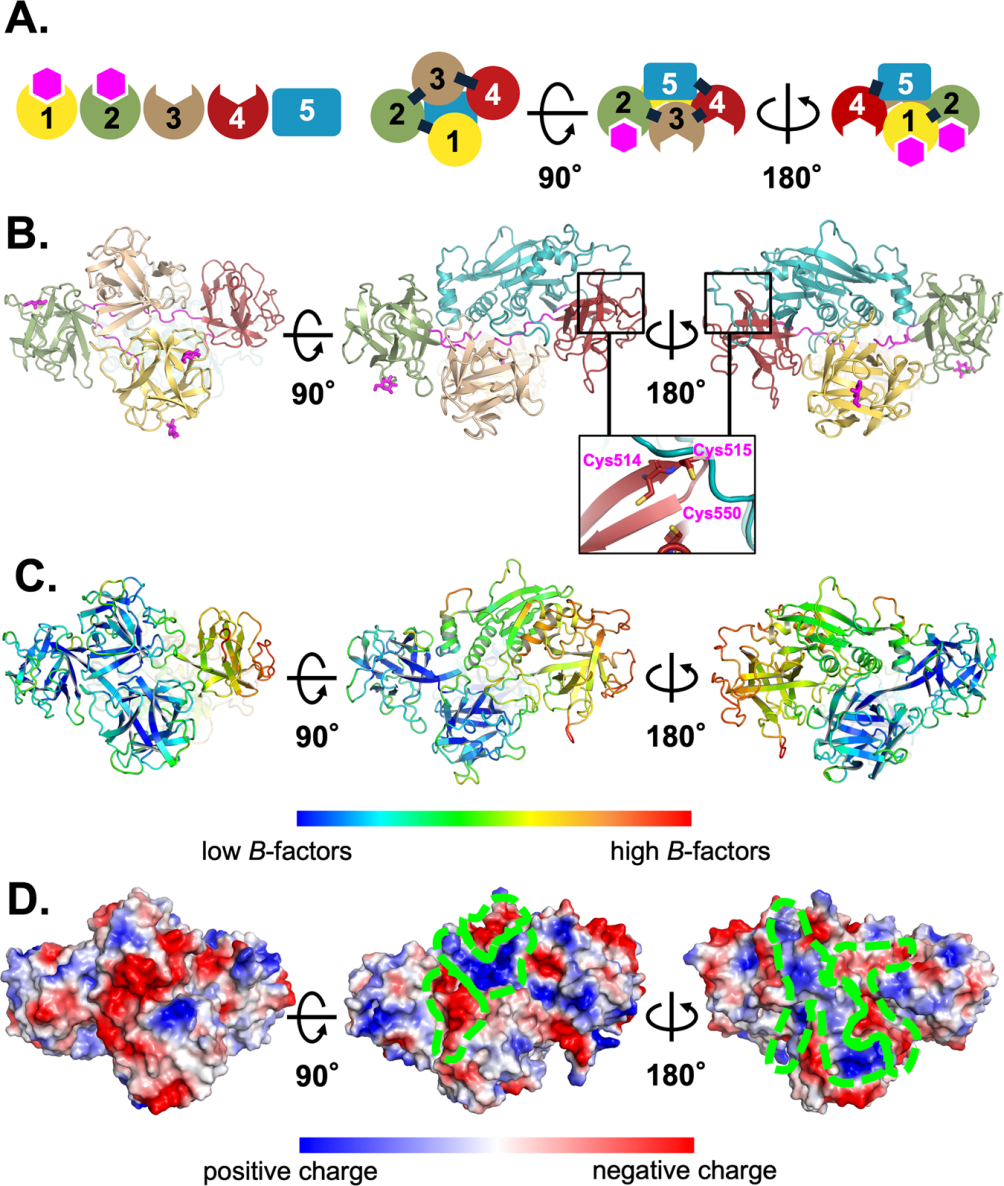
Cryo-EM structure of CCTX2. (**A**) Schematic representation of the CCTX2 domain sequence and topology in the protein tertiary structure. Each domain is represented by a different color: yellow (β-trefoil domain/BTF domain 1), green (BTF domain 2), wheat (BTF domain 3), red (BTF domain 4), and teal (C-terminal domain of unknown function; DUF). The hexagons in magenta represent sugars observed bound to the BTF domains with proposed lectin activity (see also (**B**) and Appendix Fig. S5). (**B**) Cartoon representation of the CCTX2 structure. The domains follow the color code assigned in A, with linkers between domains colored in magenta. d-galactose molecules (see Appendix Fig. S5)—likely remnants from the purification procedure—are depicted as magenta-colored sticks. They were visible in the Coulomb potential map density at the β and γ sugar-binding sites of domain 1 and the α sugar-binding site of domain 2. The inset shows the position of a putative zinc-binding site (see also Appendix Fig. S6). (**C**) Schematic presentation of CCTX2, colored by *B*-factors of Cα atoms, according to the color chart on the bottom. (**D**) Electrostatic vacuum potential of the CCTX2 surface, aligned to the representation of (**B**). The dashed green lines mark areas showing a homogenous negative or positive surface charge. The central view (“front”) shows a negative-positive-negative pattern that extends over the putative active cleft of the DUF domain. The right view (“back”) shows a semi-continuous positively charged area extending over the cleft formed by the DUF domain and BTF domains 1 and 4, and a negatively charged area extending over the cleft formed by the DUF domain and BTF domains 1 and 2.

Sequence analysis revealed that CCTX1, CCTX2, and CCTX3 exhibit four ricin B chain-like domains at the N-terminus, followed by a 220–230 amino acid domain with no homolog in the characterized proteome (Appendix Fig. S1D). The first two domains of each paralogue contain the canonical (QxW)_3_ motif (Hazes, 1996), characteristic of R-type lectins (Appendix Fig. S1D) (Yao et al, 2011), implying a possible lectin function (i.e., binding to glycans or glycoconjugates). An in silico structural model, generated in the pre-AlphaFold era (Jumper et al, 2021) using the Robetta server (Kim et al, 2004; Yang et al, 2020), showed that the ricin-B-chain-like domains adopt the stereotypical β-trefoil fold (BTF); no fully reliable model could be generated for the C-terminal domain, or the tertiary structure of CCTX2 (Appendix Fig. S3). To confirm these predictions and gain a better understanding of the molecular function of the individual domains and their interplay, we determined the CCTX2 structure by single-particle cryo-EM to a maximum resolution of 3.2 Å (Fig. 2; Appendix Fig. S4 and Table S2).

Residues 1–787 (full sequence) were visible in the reconstructed volume; regions with poorly resolved density are reported in Appendix Fig. S3D and Table S3. The structure shows a single polypeptide chain with five identifiable domains connected by short linkers (Fig. 2A), matching our initial predictions; domain boundaries are reported in Table S4. Overall, the protein adopts a compact, globular fold (Fig. 2A,B). *B*-factor analysis shows the presence of two distinct structure-functional units: BTF domains 1–3, characterized by low flexibility (low *B*-factors), and domains 4–5, showing much higher flexibility/disorder (Fig. 2C).

The four ricin-B-chain-like domains adopt the expected β-trefoil fold (BTF) and are arranged in a rhomboid shape, forming a cradle that supports the fifth domain (Fig. 2A,B). Each BTF domain displays the characteristic arrangement of the α, β, and γ subdomains around a pseudo-threefold axis (Appendix Fig. S5A); α, β, and γ subdomain boundaries are reported in Appendix Table S4. A per-domain search for structural homologs using the DALI server (Holm, 2022), limiting the results to the PDB25 data set, returned matches among other fungal lectins and bacterial toxins (Appendix Table S5). Structural alignment shows strong similarity among the four BTF domains, despite low sequence conservation (Appendix Table S6); however, a visual inspection revealed that the fourth BTF domain contains an incomplete β subdomain (Appendix Fig. S5A). The putative sugar-binding sites in all the BTF domains of CCTX2 are located on the outer side of the “cradle” and are thus fully exposed to the solvent (Appendix Fig. S5B,C). The reconstructed Coulomb potential map showed additional density (Appendix Fig. S5D) at the sugar binding pockets of subdomains β and γ of the first BTF domain and subdomain α of the second BTF domain (sites marked in magenta in Fig. 2A; Appendix Fig. S5). The detected density can probably be traced to residual D-galactose, used throughout the purification process. The canonical (QxW)_3_ motifs of R-type lectins are usually involved in the binding of glycans (Yao et al, 2011; Cummings et al, 2022), which is in good agreement with the presence of an intact (QxW)_3_ motif only in the first two BTF domains. Finally, three Cys residues (514, 515, 550) form a putative Zn^2+^ binding site, nestled into BTF domain 4 (Fig. 2B, inset). An AlphaFold3 simulation (Abramson et al, 2024) by including zinc as cofactor, placed a Zn^2+^ ion at the expected location, suggesting that CCTX2 likely binds zinc (Appendix Fig. S6).

**Figure 3:**
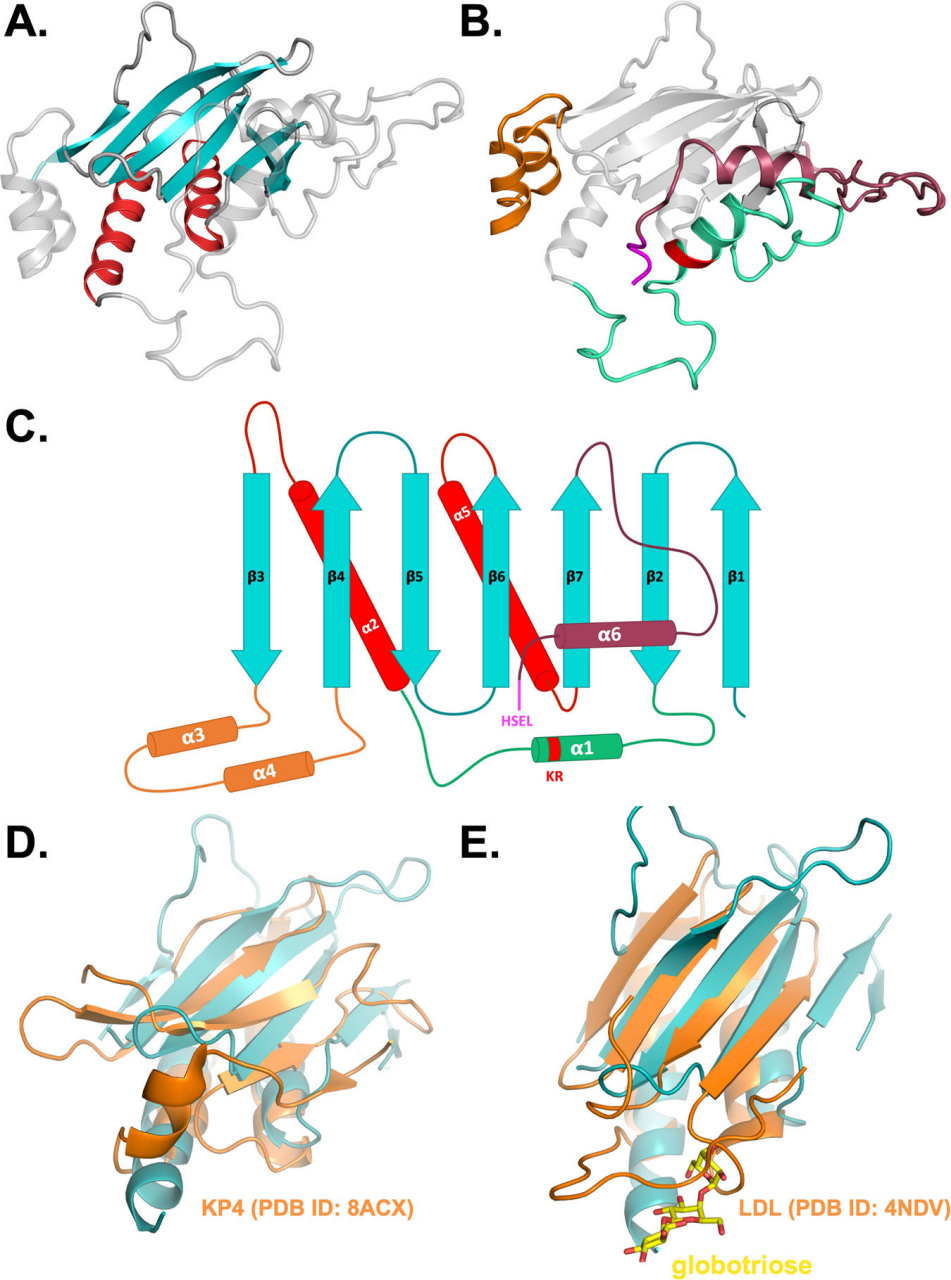
CryoEM structure of CCTX2 C-terminal Domain of Unknown Function (DUF). (**A**) Cartoon representation of the C-cryo-EM structure of the C-terminal DUF. The core elements of the fold, the central seven-stranded β-sheet and the two supporting α-helices are indicated in teal and red, respectively. (**B**) Cartoon representation of the DUF fold indicating insertions and conserved sequons. The KR sequon for kexin cleavage (residues 596–597) in the first insertion (residues 571–619, light green) is highlighted in red. The two-helix insertion (residues 656–683) is shown in orange. The C-terminus (residues 746–783) is colored dark red, with the HSEL sequon (residues 784–787) highlighted in magenta. (**C**) Topology diagram of the C-terminal DUF indicating insertions and conserved sequons shown in (**A**, **B**). (**D**, **E**) DUF domain core structural homologs identified by DALI. (**D**) Structural alignment with the killer protein 4 (KP4) orthologue from *Zymoseptoria tritici* (PDB ID: 8ACX (de Guillen et al, 2025)). (**E**) Structural alignment with the *Lyophyllum decastes* lectin (LDL; PDB ID: 4NDV (van Eerde et al, 2015)). The structure captured the lectin bound to a globotriose molecule (Galα1,4Galβ1,4Glc), represented as sticks and colored in yellow.

The fifth domain (amino acids 557–787) adopts an ɑ+β-fold (Fig. 3A,B). The core of the C-terminal domain is formed by a seven-stranded antiparallel β-sheet, supported by two α-helices; the β-sheet changes orientation between strands 6 and 7 (Fig. 3A,C). A large portion of the β-sheet is solvent-exposed and forms a potential catalytic cleft. The core of the domain is decorated with two structural insertions (Fig. 3B,C). The first insert (residues 571–619) contains a helix and a long stretch of random coil that tightly interacts with the third BTF domain; the second insert (residues 656–683) forms a two-helix subdomain, packed at the side of the central core (Fig. 3B,C). The C-terminal residues (746–787) form a long random coil segment, circling the fourth BTF domain and ending in a helix and another segment of random coil, sandwiched between the first insert and the central core (Fig. 3B,C). Interestingly, the C-terminal loop is in direct contact with the putative Zn^2+^ binding site of CCTX2 (Fig. 2B, inset; Appendix Fig. S6). Analysis of the electrostatic surface potential of CCTX2 shows areas with homogenous charge (Fig. 2D). In particular, the putative binding cleft shows a large patch of negative surface charge, while the adjacent area displays a positive charge (Fig. 2D).

A search for structural homologs of the DUF domain was performed using the DALI server (Holm, 2022). A query against the Protein Data Bank (PDB) yielded twelve structural homologs (Appendix Table S7). All of them displayed very low sequence identity with CCTX2 (max 15% seq. id.). A visual inspection of the result shows that only four hits are real matches, with the homology limited to a weak conservation of the DUF core elements (entries marked in red in Appendix Table S7 and Fig. S7). A second DALI search, conducted using the DUF domain core (amino acids 557–570, 620–655, and 684–745), returned several more hits (> 100). Careful visual inspection of the output allowed the rejection of most of these, reducing the list to twenty-two entries (Appendix Table S8; representative hits in Fig. 3D,E and Appendix Fig. S8) containing, e.g., several pilins and orthologues of killer protein 4. Several homologs bind a wide range of ligands at the central β-sheet (fatty acids, sugars, nucleic acids, cofactors or proteins; e.g., Appendix Fig. S7A,B,D; Fig. S8A). Some of the structural superimpositions suggest that the CCTX2 active site may be placed at the BTF-1/BTF-2/DUF domain cleft, rather than at the solvent-exposed β-sheet of the DUF domain (Appendix Fig. S8C,D). Interestingly, the surface potential map shows semi-continuous areas of homogenous charge extending over the BTF-1/BTF-2/DUF and BTF-1/BTF-4/DUF interdomain clefts (Fig. 2D).

### Nematotoxicity of CCTX2 is dependent on the two N-terminal lectin domains and the C-terminal DUF domain

**Figure 4:**
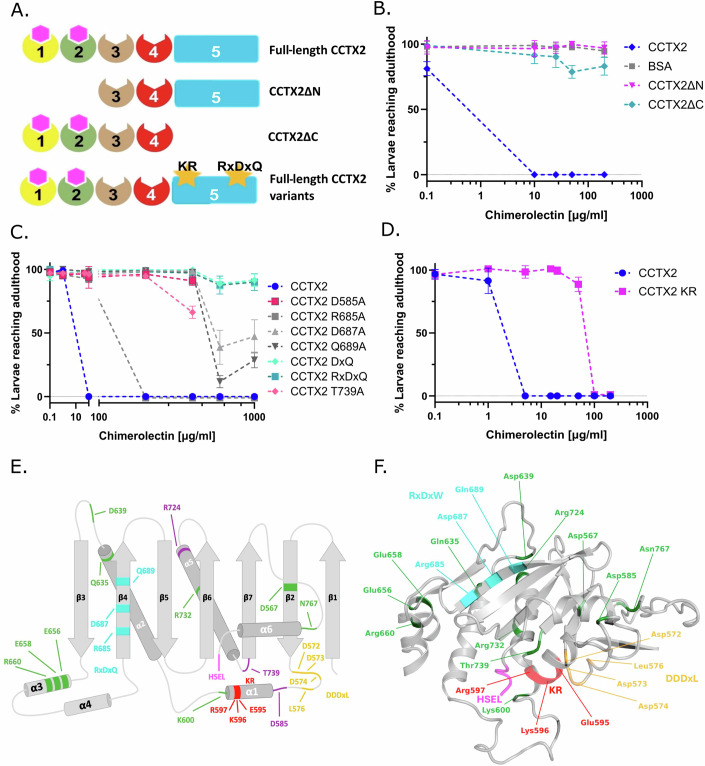
The two N-terminal BTF lectin domains and the C-terminal DUF domain are required for CCTX2 nematotoxicity. (**A**) Schematic representation of the CCTX2 architecture with KR and RxDxQ motifs indicated by yellow stars (see main text for details). (**B**) *C. elegans* nematotoxicity assay with full-length CCTX2 (blue), a CCTX2 variant lacking the two N-terminal BTF domains (CCTX2ΔN, magenta), and a variant lacking the C-terminal domain of unknown function (CCTX2ΔC, teal), with BSA used as a negative control. (**C**) *C. elegans* nematotoxicity assay with CCTX2 variants in the RxDxQ motif either as single substitutions (gray, R685A, D687A, and Q689A) or double (DxQ, light teal) or triple alanine replacements (RxDxQ, teal). Point mutations D585A and T739A also conferred resistance to the toxin. (**D**) Resistance of *C. elegans* to CCTX2-KR (E595A, K596A, R597A) in nematotoxicity assay. Data points with error bars indicate means of *N* = 4 biological replicates with standard error of the mean (SEM). Overlapping data points were nudged by +/− 2 data units in *y* for better readability. (**E**) Topology diagram of the C-terminal DUF reporting the position of amino acids targeted by site mutagenesis. (**F**) Stereo cartoon representation of the C-terminal DUF, with residues targeted by Ala-scanning substitutions (green) mapped onto the cryo-EM structure. Positions corresponding to the key functional motifs KR (Glu595, Lys596, Arg597; red), RxDxQ (Arg685, Asp687, Gln689; cyan), DDDxL (Asp572, Asp573, Asp574, Leu576; yellow), HSEL (C-terminal residues 784–787; magenta) and three variants significantly affecting toxicity (Asp585, Arg724, Thr739; purple) have been marked in different colors. Source data are available online for this figure.

To characterize the molecular functions of the different CCTX2 domains, we generated both N- and C-terminally truncated variants of the protein (Fig. 4A). Since both sequence and structural analysis predicted a lectin function for the first two BTF domains, we produced a variant lacking these two domains (residues 304–787), termed CCTX2∆N. To assess carbohydrate-binding, we probed AlexaFluor488-labeled versions of both full-length CCTX2 and CCTX2∆N for binding to the CFG mammalian glycan array v5.1, using the service provided by the Consortium of Functional Glycomics (CFG, Emory University, USA). The data show that CCTX2 is indeed a lectin with specificity towards LacNAc(Galβ1-4GlcNAc)- and LacdiNAc(GalNAcβ1-4GlcNAc)-containing glycans (Appendix Fig. S9 and Supplementary Datasets EV1–5). No binding was detected for CCTX2∆N (Supplementary data files), but the protein was soluble, suggesting structural and functional integrity. Overall, results indicate that the first two BTF domains of CCTX2 are required for its carbohydrate-binding activity, in agreement with the sequence predictions and the structural results. To test whether the carbohydrate-binding activity of CCTX2 is necessary for its nematotoxicity, we analyzed CCTX2∆N with our nematotoxicity assay (Fig. 4B). We found that this protein variant was not nematotoxic, strongly suggesting that the lectin function is essential for the nematotoxicity of CCTX2.

Although the C-terminal domain does not show strong similarity (> 20% identity) to proteins of known function in the NCBI database using a BLASTP search, it is well-conserved among the three CCTX paralogues, with a sequence identity of approximately 40% (residues 555–787). To assess its role for nematotoxicity, we created a variant of CCTX2, termed CCTX2ΔC, which lacks this domain (residues 570–787) (Fig. 4A). This variant exhibited severely reduced nematotoxicity, indicating that this domain is also essential to mediate fungal defense (Fig. 4B). Currently, we can only speculate about the molecular function of this domain. An amino acid sequence alignment between the members of the monophyletic clade of CCTX homologs revealed several conserved amino acid residues within the domain (Appendix Fig. S1D). We individually replaced sixteen of these residues with alanine and evaluated the toxicity of the resulting CCTX2 variant proteins in our nematotoxicity assay against *C. elegans* (Appendix Fig. S1D and Table S9). We also mapped the residues onto the structure (Fig. 4E,F). Nine of these CCTX2 variants showed a reduction in toxicity of CCTX2 toward *C. elegans* (Fig. 4C; Appendix Table S9, Appendix Fig. S10A,B). Of these, substitutions D687A and Q689A had the strongest effects with a reduction of the toxicity of CCTX2 towards *C. elegans* by two orders of magnitude (Fig. 4C, Appendix Fig. S10A). A double replacement of both residues (“DxQ” variant), as well as a triple variant (“RxDxQ” variant) that includes neighboring residue Arg685, the individual substitution of which caused only a weak reduction in toxicity (Fig. 4C), rendered the protein completely non-toxic. We renamed these three amino acids the “RxDxQ motif” (referring to Arg685, Asp687 and Gln689 in CCTX2) (Fig. 4C). In the CCTX2 structure, this motif is exposed on the surface of the central β-sheet (Fig. 4E,F). The single substitutions D585A and T739A in the C-terminal domain also resulted in a significant reduction in toxicity (non-toxic up to a concentration of 500 mg/l), while the variants R724A as well as Q635A and E656A retained more toxic activity, and were non-toxic at a concentration below 200 mg/l and 100 mg/l, respectively (Appendix Figs. S10A and S11 and Table S9).

Apart from the ‘RxDxQ motif’, we found that the C-terminal domain contains a conserved putative cleavage site (Lys596 and Arg597 in CCTX2) for the Golgi-localized endoproteinase kexin (Figs. 3B,C and 4E,F). Substitution of these two residues (and the preceding Glu595) to alanine in CCTX2 resulted in a reduction of toxicity of this protein variant, CCTX2-KR, against *C. elegans* (Fig. 4D). This site is embedded in the second structural insert, on a solvent-exposed α-helix (Fig. 4F). To check whether this motif was functional, purified CCTX2 protein was incubated with Kex2p protease from *Saccharomyces cerevisiae* and the reaction was subjected to a subsequent pulldown experiment using Sepharose beads. Analysis of the various fractions by SDS-PAGE and immunoblotting, using a custom-made anti-CCTX2 antiserum, showed that CCTX2 was cleaved into two fragments of ≈23 and ≈67 kDa, corresponding to the molecular masses expected from a proteolytic processing at the presumptive cleavage site (Appendix Fig. S10C). Intriguingly, however, Kex2p cleavage alone was insufficient to separate the two fragments, as we could not detect the C-terminal fragment, neither in the pulldown flow-through nor in the wash. In conclusion, the C-terminal fragment of CCTX2 is tightly associated with the N-terminal fragment and can only be separated under denaturing conditions, such as SDS-PAGE (Appendix Fig. S10C). An AlphaFold3 simulation of the two peptides generated by Kex2p cleavage suggests a possible conformational change (Appendix Fig. S12). The in silico model predicts that in the cleaved protein, the C-terminus (residues 779–787) shifts to occupy the putative catalytic cleft of CCTX2 (Appendix Fig. S12A). In the new conformation, amino acids of the RxDxQ motif would stabilize the C-terminus, and the HSEL motif would become fully exposed to the solvent (Appendix Fig. S12A,C).

Finally, we noticed a conserved motif, Asp572 to Leu 576, which we refer to as DDDxL, located between BTF4 and the “KR motif” (Fig. 4E,F). Substitution of all three Asp residues and the terminal Leu residue of this motif to alanine resulted in a protein (CCTX2-DDDxL) with reduced toxicity (non-toxic at a concentration below 100 mg/l) (Appendix Figs. S10A and S11 and Table S9) to a similar extent as the single substitutions of residues Gln635 and Glu656 (see above).

Taken together, this mutational analysis confirmed that the C-terminus of CCTX2 is essential for its nematotoxicity but did not reveal the biochemical function of the DUF domain.

### CCTX2 binds to *C. elegans* intestinal epithelial glycosphingolipids in vivo and in vitro

**Figure 5:**
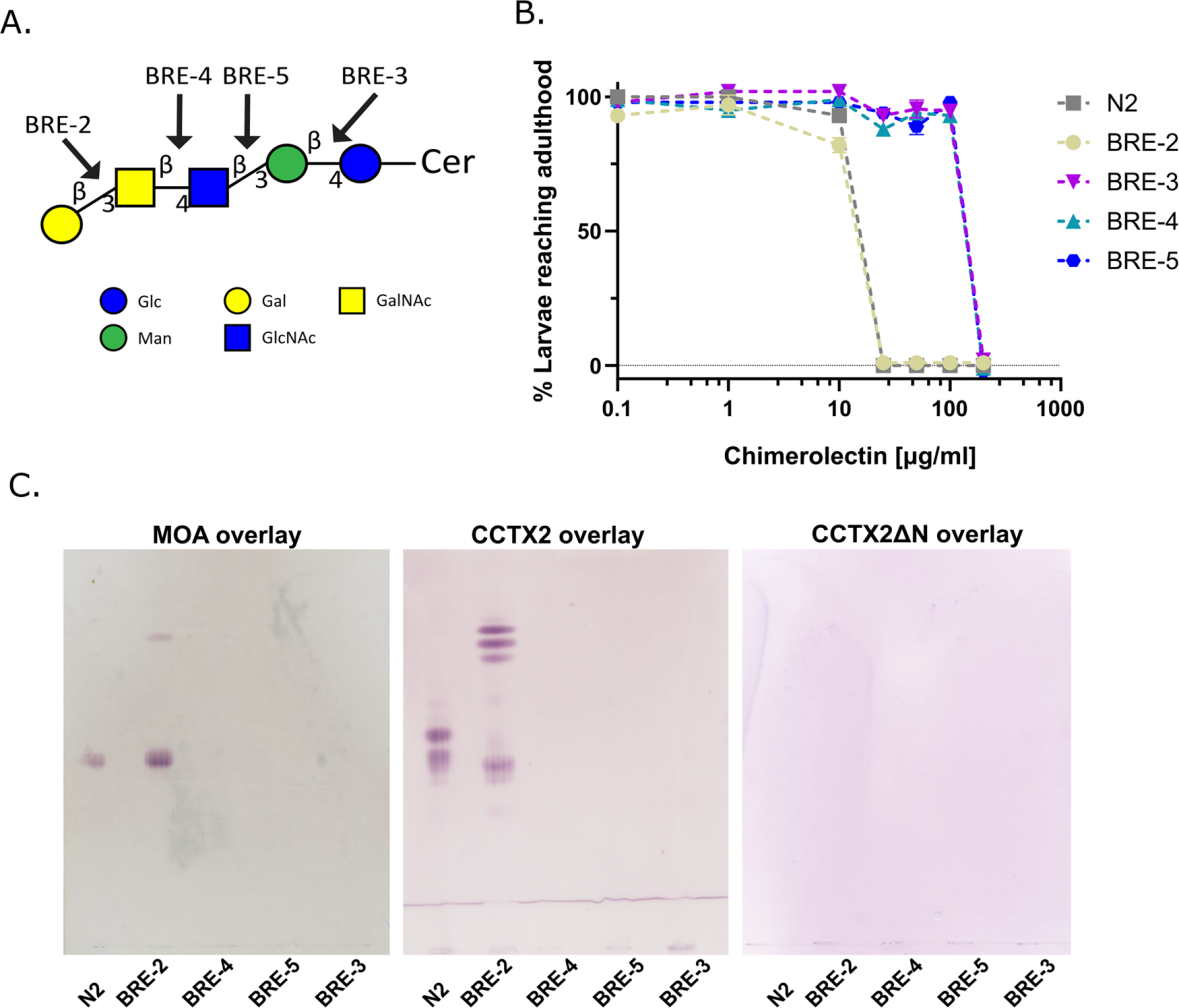
CCTX2 binds to *C. elegans* intestinal epithelial glycosphingolipids in vivo and in vitro. (**A**) Schematic representation of the arthroseries GSL core, including the *C. elegans* enzymes responsible for the addition of individual monosaccharides. The ceramide-linked core is synthesized by sequential action of the enzymes BRE-3, BRE-5, and BRE-4; GalNAc is transferred by BRE-2. Monosaccharides are represented as introduced by the Consortium for Functional Glycomics (CFG) (Varki et al, 2015). (**B**) Toxicity assay on *C. elegans* GSL biosynthesis mutants *bre-3(ye26), bre-5(ye-17), bre-4(ye27)* and *bre-2(ye31)*. L1 larvae of mutant and wild-type worms were fed with different concentrations of CCTX2 (up to 200 µg/ml) and scored for the percentage of larvae reaching the L4 stage/adulthood. *C. elegans* N2 and the mutant for the GalNAc transferase *bre-2(ye31)* were sensitive to CCTX2 at low concentrations. Data points with error bars indicate means of *N* = 4 biological replicates with standard error of the mean (SEM). Overlapping data points were nudged by +/− 2 data units in *y* for better readability. (**C**) TLC overlays of upper phase GSLs isolated from the respective *C. elegans* GSL biosynthesis mutants and N2 wild-type strains, blotted with 150 nM of the indicated biotinylated protein. Source data are available online for this figure.

The dependence of the nematotoxic activity of CCTX2 on two lectin domains (Fig. 4B) and the localization of TAMRA-labeled CCTX2 to the intestinal epithelial membrane in *C. elegans* (Fig. 1D,E) suggest that this chimerolectin targets a glycoconjugate present in the apical intestinal plasma membrane of nematode enterocytes. The specificity of CCTX2 towards LacNAc and LacdiNAc-containing motifs (Appendix Fig. S9), the binding of nematotoxic chimerolectin MOA to *C. elegans* glycosphingolipids (GSLs) (Wohlschlager et al, 2011), and the presence of LacdiNAc in the core of the glycan part of *C. elegans* GSLs (Barrows et al, 2006) point to these glycoconjugates as putative target molecules. The biosynthesis of the *C. elegans* GSL glycans occurs by the sequential action of the enzymes BRE-3 (β1,4-mannosyltransferase), BRE-5 (β1,3-*N*-acetylglucosaminyl transferase), BRE-4 (β1,4 *N*-acetylgalactosaminyl transferase), and BRE-2 (β1,3-galactosyltransferase) (Fig. 5A) (Barrows et al, 2006). To test the role of GSLs in toxin binding, loss-of-function *C. elegans* mutants of the four GSL glycosyltransferase-encoding genes were tested for resistance towards CCTX2. All the tested *C. elegans* GSL mutant strains displayed increased resistance to CCTX2, except for the *bre-2(ye31)*-mutant strain (Fig. 5B). This result strongly suggests that the LacdiNAc motif in the core of the *C. elegans* GSLs is the glycoepitope targeted by CCTX2. To demonstrate direct binding of CCTX2 to *C. elegans* GSLs, we performed CCTX2 protein overlays for GSLs isolated from wild-type and mutant *C. elegans* strains. *C. elegans* GSLs were separated by Thin Layer Chromatography (TLC) and overlayed with biotinylated CCTX2, using biotinylated MOA as a positive control. The results showed binding of CCTX2 to GSLs from *C. elegans* N2 wild-type and the *bre-2(ye31)* mutant strain, similar to the MOA positive control (Fig. 5C). No binding of CCTX2 or the MOA positive control to the GSLs isolated from *bre-3, -4*, or* -5* strains was observed, consistent with the resistance of those strains to both proteins (Wohlschlager et al, 2011). In addition, we could not observe any binding to GSLs for the CCTX2∆N variant, which lacks the carbohydrate-binding domains (Fig. 5C).

### CCTX2 undergoes endocytosis and retrograde trafficking in *C. elegans* enterocytes

**Figure 6:**
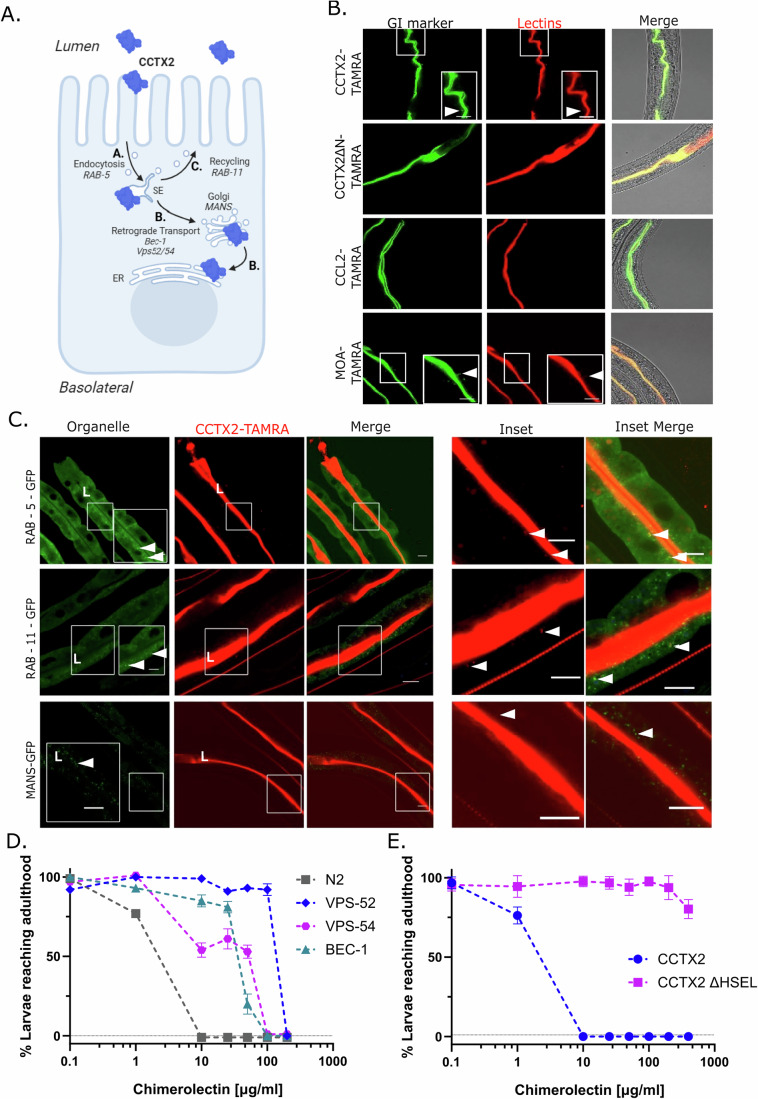
Endocytosis and retrograde trafficking of CCTX2 in *C. elegans* enterocytes. (**A**) Schematic representation of the endocytic network in *C. elegans* enterocytes. After endocytosis and fusion of the endocytic vesicles with the sorting endosome (SE) (1), the cargo is sorted either for recycling back to the plasma membrane (3) or for retrograde trafficking to the TGN, Golgi, and endoplasmic reticulum (ER) (2). Cargo destined for degradation is sorted into the late endosome (LE) and the lysosomal pathway. (**B**) Internalization of CCTX2 into *C. elegans* enterocytes. Fluorescently labeled CCTX2 (red), truncated CCTX2∆N (red), the GSL-binding chimerolectin MOA (red), or the N-glycan binding lectin CCL2 (red) were fed to *C. elegans* GK70 ('GI marker', green) for 3 h at 20 °C. Arrowheads indicate fluorescent puncta (vesicles) as a sign of endocytosis. These vesicles appear red (due to the TAMRA-labeled cargo), green (due to the intestinal apical membrane marker PGP-1-GFP) or yellow (merge). (**C**) Co-localization of CCTX2 with endocytic markers. *C. elegans* strains expressing GFP-labeled RAB-5 (RAB-5-GFP), RAB-11 (RAB-11-GFP) as well as the Golgi marker α-Mannosidase II (MANS-GFP) were used as endocytic and retrograde trafficking markers to test for co-localization with CCTX2. TAMRA-labeled CCTX2 was fed to the respective *C. elegans* L4 larvae for 6 h at 20 °C and monitored for co-localization. Images show the intestinal cross-sections. Arrowheads indicate overlapping puncta (vesicles). “L” indicates the position of the intestinal lumen. Scale bars = 10 µm. (**D**) Nematotoxicity assay with CCTX2 with different *C. elegans* endosome-to-Golgi retrograde trafficking (transport) mutants: Strains VC625 and VC985 bear mutations in the genes coding for the GARP complex subunits VPS-52 and VPS-54, respectively; strain VC424 carries a mutation in the BEC-1-encoding gene which is required for retromer localization to endosomes. (**E**) Nematotoxicity assay of CCTX2 variant lacking the terminal HSEL sequon. Data points with error bars indicate means of *N* = 4 biological replicates with standard error of the mean (SEM). Overlapping data points were nudged by +/− 2 data units in *y* for better readability. Source data are available online for this figure.

Many bacterial GSL-binding AB toxins (e.g., Shiga or cholera toxins (Sandvig et al, 2014)) are endocytosed upon binding to a glycolipid receptor (Fig. 6A). We therefore hypothesized that the fungal chimerolectins CCTX2 and MOA follow a similar pathway. In contrast, toxic hololectins, which bind to and cluster *N*-glycoprotein receptors, i.e., protein-bound glycans (e.g., CGL2 (Butschi et al, 2010) and CCL2 (Schubert et al, 2012)), might not require internalization and exert toxicity by a different mechanism (Stutz et al, 2015; Bleuler-Martinez et al, 2017). To assess if endocytosis of CCTX2 was required for its nematotoxicity, we fed TAMRA-labeled CCTX2 to L4 larvae of *C. elegans* GK70, a strain carrying the apical plasma membrane marker PGP-1 fused to GFP, and monitored the localization of CCTX2 using confocal fluorescence microscopy. The fungal chimerolectin MOA and the hololectin CCL2 were included for comparison. After 3 h of feeding, intracellular TAMRA-positive vesicles were observed in *C. elegans* enterocytes (Fig. 6B). Several endocytic vesicles co-stained with PGP-1-GFP were detected, indicating active endocytosis of the chimerolectin CCTX2. Indeed, we also observed such endocytic vesicles for TAMRA-labeled and GSL-binding chimerolectin MOA. In contrast, we did not detect TAMRA-CCL2 in endocytic vesicles, which is in agreement with previous studies (Stutz et al, 2015). In addition, we could not detect endocytic vesicles with the CCTX2∆N variant (Fig. 6B), suggesting again that GSL receptor binding is necessary for endocytosis of the protein toxin.

To confirm the endocytosis of CCTX2 in enterocytes, we used *C. elegans* strains with fluorescently marked endocytic compartments (Fig. 6A,C). For this purpose, we fed TAMRA-labeled CCTX2 to *C. elegans* strains expressing GFP-labeled RAB-5 (small GTPase present on early endosomes (Bucci et al, 1992)), RAB-11 (a small GTPase on recycling endosomes (Hoekstra et al, 2004; Jing and Prekeris, 2009; Sato et al, 2014)); and MANS (α-Mannosidase II,an enzyme localized in the Golgi (Shah et al, 2008)). After 6 h of feeding, double-positive intracellular vesicles containing both CCTX2 and the endocytic marker protein were observed in enterocytes of the *rab-5::GFP* strain (Fig. 6C), confirming that fluorescently labeled CCTX2 was endocytosed upon receptor binding. Co-localization of CCTX2 and RAB-11-GFP was also observed (Fig. 6C). Localization of CCTX2 to downstream organelles such as the Golgi apparatus (MANS-GFP) was rarely detected.

After endocytosis, many GSL-binding AB toxins hijack retrograde vesicle trafficking to reach the endoplasmic reticulum (ER) (Teter, 2013). From this compartment, many AB toxins are released into the cytoplasm by retro-translocation, where they exert their toxic activity (Sandvig and van Deurs, 2005; Sandvig et al, 2010; Sandvig et al, 2013). The retrograde trafficking of CCTX2-containing vesicles and its requirement for nematotoxicity were evaluated using *C. elegans* strains deficient in different components of the endosome-to-Golgi retrograde trafficking machinery. Deletions in the genes coding for GARP (Golgi-associated retrograde protein) complex subunits VPS-52 and VPS-54, rendered the *C. elegans* partially resistant to intoxication with CCTX2 (Fig. 6D). In addition, a balanced knock-out mutation of the *bec-1* gene, whose product is, in conjunction with phosphatidylinositol-3-kinase (PI3K) VPS-34, required for the recruitment of the retromer protein complex to endosomes, led to increased resistance to CCTX2 compared to the *C. elegans* N2 wild-type strain (Fig. 6D). Neither of these strains showed increased resistance towards MOA or CCL2, indicating that their resistance towards CCTX2 was toxin-specific and not a pleiotropic effect due to overall changes in membrane or trafficking homeostasis (Fig. S2). Given that the intracellular target for the chimerolectin MOA is unknown, we speculate that this chimerolectin might not require retrograde trafficking to reach its target or uses a different trafficking route.

The three *C. cinerea* paralogues contain a conserved C-terminal four-residue sequence (HSEL) at their C-termini, resembling the canonical ER-retention signal (KDEL), present, e.g., in cholera toxin for retention in the ER (Fig. 3B,C; Appendix Fig. S1D) (Lencer et al, 1995; Newstead and Barr, 2020). Removal of the sequon from CCTX2 (CCTX2ΔHSEL) abolished toxicity of the protein towards *C. elegans* N2 (Fig. 6E). This indicates that the presence of an intact HSEL sequon is a prerequisite for toxicity, and its removal likely affects either translocation into the ER or retention of the protein in this compartment.

Taken together, our results suggest that CCTX2 is internalized into early endosomes (RAB-5-dependent). From there, the toxin can be recycled back to the plasma membrane via RAB-11-positive vesicles or undergo retrograde trafficking to the Trans-Golgi Network (TGN), Golgi, and the ER facilitated by the GARP complex (VPS-52 and VPS-54) and the PI3K machinery (BEC-1).

## Discussion

Fungal lectins have been implicated in defense against foraging nematodes or insects (Bleuler-Martínez et al, 2011; Wohlschlager et al, 2011; Tayyrov et al, 2018a). In the present work, we describe CCTX1, CCTX2, and CCTX3, three nematotoxic chimerolectins from the basidiomycete *C. cinerea*. We performed an in-depth investigation of CCTX2, the holotype of this new family of fungal chimerolectins, and provided a structure–function analysis by characterizing its cryo-EM structure, its target glycoconjugates, and its internalization route in the model nematode *C. elegans*.

### CCTX family of chimerolectins and its holotype CCTX2

CCTX1, CCTX2, and CCTX3 are the first known representatives of a new family of fungal defense proteins with a mechanism of action reminiscent of bacterial AB toxins. AB toxins are relatively rare in the kingdom of fungi, with MOA and its homolog PSL1a being the most prominent members described so far (Wohlschlager et al, 2011; Juillot et al, 2016). Orthologues of the CCTX family are restricted to the fungal kingdom (Fig. 1B), predominantly in the phyla Basidiomycota and Ascomycota. Intriguingly, the closest sequence homolog of the CCTX toxins can be found in *Trichoderma harzianum*, a pathogen of plants and other fungi without a known sexual stage (Gams and Meyer, 1998) (THTX2, UniProt ID: A0A0F9ZAH1).

The *cctx1*, *cctx2*, and *cctx3* genes are located next to each other in the genome of *C. cinerea*, suggesting that they are the result of gene duplication events. Similar tandem arrangements of defense genes can be found in *C. cinerea* for nematotoxic hololectins CGL1-CGL2 (*C. cinerea* galectin) and CCL1-CCL2 (Boulianne et al, 2000; Schubert et al, 2012) as well as bacteriocidal cysteine-stabilized β-defensin CPP1 (Copsin)/CPP2 (*C. cinerea* Copsin paralogue) (Kombrink et al, 2019a). Gene duplications allow organisms to diversify a gene without losing its original function (Moore and Purugganan, 2003). Similar to the other known examples of duplicated *C. cinerea* defense genes, we observed diversification of the *cctx* genes at the level of transcriptional regulation. Whereas *cctx2* is expressed at low levels only in the vegetative mycelium and induced upon challenge with fungivorous nematodes (Plaza et al, 2016), *cctx1* and *cctx3* are constitutively expressed at significant levels in young fruiting bodies and vegetative mycelium, respectively (Appendix Fig. S1A,B). The signals and signaling pathways leading to the induction of *cctx2* upon nematode challenge are subject to current investigations. Previous results indicate that active feeding by the fungivorous nematode is needed and that a sudden drop in the turgor pressure caused by the nematode feeding could be a possible trigger (Plaza et al, 2016; Guillaume-Gentil et al, 2022).

In silico predictions of CCTX2 (Appendix Fig. S3A,B) suggested that CCTX family members are composed of five domains: four N-terminal BTF domains and a C-terminal domain of unknown function. This arrangement resembles that of AB_4_ toxins, represented by MTX from *Bacillus sphaericus* and pierisin-1 from *Pieris rapae*, the cabbage butterfly (Treiber et al, 2008; Oda et al, 2017). However, these toxins display a swapped domain organization, with the four BTF domains placed *C-terminally* and the effector domain at the *N-terminus*. The structure of CCTX2, here determined by cryo-EM to 3.2 Å resolution, confirms the β-trefoil fold for the N-terminal domains, and reveals their homology with BTF domains from other fungal lectins and bacterial toxins (Appendix Table S5), including MOA (Grahn et al, 2007; Grahn et al, 2009), PSL1a (Kadirvelraj et al, 2011) and the *Clostridium botulinum* hemagglutinin component (HA1) (Inoue et al, 2003). The BTF domains of CCTX2 cluster in a rhomboid fashion, forming a cradle for the fifth domain (Fig. 2A,B). This differs from MTX or pierisin-1, where the four BTF domains are arranged in a linear fashion around one side of the effector domain (Treiber et al, 2008) (Appendix Fig. S13). Furthermore, the C-terminal domain of CCTX2 does not show any sequence conservation or structural similarity to the effector domain of MTX and pierisin-1, which in both proteins has ADP-ribosyltransferase activity (Treiber et al, 2008; Oda et al, 2017).

BTF domains often have lectin activity, and, accordingly, CCTX2 was confirmed to be a lectin by glycan array analysis, specifically recognizing the LacNAc and LacdiNAc glycoepitopes (Appendix Fig. S9). The first two N-terminal BTF domains appear to be required for sugar binding, as the N-terminal deletion construct CCTXΔN failed to bind to the array (Supplementary data files). Although we cannot exclude that the N-terminal deletion causes structural changes that may compromise glycan binding activity, our conclusion is supported by the fact that the protein behaves well upon production and purification and that the putative sugar-binding sites are clustered on the same, solvent-exposed molecular surface (Appendix Fig. S5). Moreover, QxW motifs β and γ on the first domain and site α on the second domain show additional density, which likely corresponds to bound D-galactose from the purification process. This indicates that the two N-terminal BTF domains 1 and 2 are important for glycan binding on GSL receptors. The lack of residual density at the putative sugar-binding sites of the third and fourth BTF domains and the less-conserved β subdomain in the fourth BTF domain suggest that BTF domains 3 and 4 have a function different from receptor recognition—potentially for structural support of the C-terminal domain.

### Endocytosis and intracellular trafficking of CCTX2

CCTX2 enters *C. elegans* enterocytes by binding to arthroseries glycosphingolipids carrying the LacdiNAc moiety (Fig. 5). *C. elegans* GSLs are also targeted by the pore-forming nematicidal toxin Cry5B from *Bacillus thuringensis* as well as by the fungal chimerolectin/AB toxin MOA (Griffitts et al, 2005; Wohlschlager et al, 2011). Glycoproteins might provide a secondary entry route, as indicated by the residual toxicity detectable in GSL-deficient mutants *bre-3*, *bre-5*, and *bre-4* (Fig. 5B). Receptor binding is likely mediated by the two N-terminal BTF lectin domains, since no GSL binding was detected for CCTXΔN (Fig. 4C). GSL binding triggers internalization of CCTX2, as the toxin colocalized with the apical plasma membrane marker PGP-1 in vesicles within *C. elegans* enterocytes (Fig. 6B). Co-localization of CCTX2 with fluorescent endocytic markers, such as RAB-5 or RAB-11, was conclusive evidence that CCTX2 undergoes endocytosis (Fig. 6C).

Several AB toxins (e.g., Shiga toxin, cholera toxin, etc.) take the same internalization route, followed by retrograde trafficking to the ER and retro-translocation to the cytoplasm (Sandvig and van Deurs, 2005; Cho et al, 2012; Teter, 2013). CCTX2 retrograde trafficking was demonstrated using several *C. elegans* mutants deficient for intracellular trafficking components (Fig. 6 and Appendix Table S10). These include mutants in the genes coding for subunits VPS-52 and VPS-54 of the GARP (Golgi-associated retrograde protein) complex, and for BEC-1 (Fig. 6D). In *C. elegans*, BEC-1 mediates autophagy and is required for retrograde transport from endosomes to the TGN by recruiting, in conjunction with the phosphatidylinositol-3 kinase VPS-34, the retromer protein complex to endosomes (Takacs-Vellai et al, 2005; Ruck et al, 2011). The toxicity resistance of the *bec-1* mutant  clearly links the toxic effect of CCTX2 to  retrograde trafficking. In this pathway, the GARP complex is localized within the TGN and required for tethering the endosome-derived vesicles to the Golgi apparatus (Luo et al, 2011). The toxin resistance of the *vps-52* and *vps-54* mutants thus indicates a strong requirement for TGN transit in CCTX2 trafficking. None of the retrograde trafficking mutants showed resistance to the chimerolectin/AB toxin MOA. This suggests that the observed effects were specific to sorting and trafficking of CCTX2, and not pleiotropic effects (Fig. 6D). Apparently, the retrograde trafficking pathways of CCTX2 and MOA substantially diverge from the endosome onwards. Retrograde trafficking of bacterial AB toxins is often required for toxicity, with ER processing being a fundamental step in toxin activation (Nowakowska-Gołacka et al, 2019). Several bacterial toxins, such as cholera toxin or *Pseudomonas* exotoxin A rely on a KDEL sequon for efficient ER targeting and accumulation by Erd2, the ER protein retention receptor (Lencer and Saslowsky, 2005). CCTX paralogues carry a conserved HSEL sequon at the C-terminus, similar to the ER-retention signal HDEL/KDEL in yeast/mammalian cells. Removal of the sequon decreased the toxicity of the protein substantially (Fig. 6E), suggesting that CCTX2 exploits the ER protein retention receptor Erd2 for vesicular trafficking. However, it is worth noting that not all CCTX2 homologs share this C-terminal KDEL-like sequon.

Structural data provide further insights into the possible translocation of CCTX2 to the ER. The HSEL sequon is hidden inside the bulk of the C-terminal domain (Fig. 4E,F), shielded from direct contact with the solvent by an α-helix (residues 589-600). However, the helix carries a putative kexin cleavage site (K596-R597), which is cleaved by the *S. cerevisiae* homolog Kex2p in in vitro assays (Appendix Fig. S10C). Kexin is a TGN protease and a lower eukaryote orthologue of furin, a peptidase involved in the activation of several toxins (e.g., Shiga toxin) (Garred et al, 1995). The fragments resulting from Kex2p cleavage do not dissociate after proteolysis (Appendix Fig. S10C), which can be explained by the highly compact fold of CCTX2. Thus, we hypothesize that proteolytic processing causes a conformational change in the protein, exposing the HSEL sequon to the solvent, allowing recognition by the KDEL receptor and transportation back to the ER.

### CCTX2 effector domain

The nematotoxicity of CCTX2 is dependent on its C-terminal domain of unknown function (Fig. 4B,C), thus identifying it as the defense effector domain. Neither BLAST nor structural searches revealed any similarities to proteins with known functions, leaving its biological role unclear. Sequence comparisons among fungal homologs and functional analysis revealed a conserved RxDxQ motif that is essential for the nematotoxicity of CCTX2 (Fig. 4C; Appendix Fig. S1D). This finding does, however, not help to draw conclusions regarding the function of the domain as such a motif has, to our knowledge, not been described for any other protein.

The cytotoxic domain adopts an α + β-fold: a large, mixed seven-stranded β-sheet supported by two α-helices constitutes its core folding motif (Fig. 3). The β-sheet exhibits a large solvent-exposed surface, which could act as a catalytic cleft, and is decorated by two insertions, mostly composed of α-helices and random coil segments. Searching the PDB for structural matches returned hits with weak structural similarity, and with a wide variety of origins and functions. Overall, structural matches hint at a ligand-binding role for the solved-exposed surface of the central β-sheet, either to small molecules (e.g., carbohydrates) or protein partners (Appendix Figs. S7 and S8). Remarkably, some homologs suggest alternative substrate binding sites, located along the cleft formed by the DUF domain with BTF-1/BTF-4 or BTF-1/BTF-2 (Appendix Figs. S8 and  S13). Several of the hits are of fungal origin and are often associated with a toxic effect (Appendix Tables S7 and S8). This is exemplified by the remarkable similarity between the core of the DUF domain and two orthologues of the killer protein 4 family (Brown, 2011). KP4 orthologues from the fungi *Zymoseptoria tritici* (ZtKP4) and *Ustilago maydis* (UmKP4) bear a strong resemblance to the DUF domain core (Fig. 3D). ZtKP4 and UmKP4 are antifungal toxins, one of which (UmKP4) is of mycoviral origin and acts on mammalian cells (Gu et al, 1995; de Guillen et al, 2025). These toxins act through an unknown mechanism, with UmKP4 showing an inhibitory effect on mammalian Ca^2+^ channels (Gu et al, 1995). Another interesting hit is the *Lyophyllum decastes* lectin (LDL; Fig. 3E), which shares glycan binding preference with Shiga and Shiga-like toxins (Goldstein et al, 2007; Sandvig et al, 2015). LDL likely acts at the fungal cell wall interface and may function in host defense, serving as a holotype of a large family of fungal and plant lectins (van Eerde et al, 2015). Despite these interesting leads, none of these structures and sequence alignments directly reveal the biochemical activity of the effector domain of CCTX2. However, they provide some hypotheses that will be tested in future experiments.

Site-directed mutagenesis targeting 23 amino acid residues conserved within the CCTX family identified D687 and Q689 as essential for *C. elegans* toxicity (Fig. 4C; Appendix Fig. S10, Table S9). Their placement at the putative catalytic cleft (Fig. 4E,F) is consistent with a potential role in substrate binding. However, the fold observed in the cryo-EM structure might not be the active form of the effector domain of CCTX2. As discussed in the context of the putative ER retention signal, the proteolytic processing by kexin (or another host protease) could trigger a structural rearrangement that considerably alters the domain topology and eventually also the overall fold of CCTX2. Interestingly, an Ala variant targeting residue Asp585, positioned in the high-*B*-factor loop immediately preceding the kexin-cleaved helix, shows a decrease in toxicity (Appendix Figs. 4C,E,F and S11A,B), suggesting its active role in dynamic changes affecting that region, such as a conformational change. An in silico folding simulation of the two fragments generated from the Kex2p-cleaved protein predicts a rearrangement of the C-terminus of CCTX2, with the KDEL sequon moving to a solvent-exposed position (Appendix Fig. S12). In this model, the C-terminus is held in place by the RxDxQ motif, providing a possible explanation for its importance for toxicity (Fig. 4C; Appendix Fig. S12). Together, this suggests that CCTX2 needs to be activated by *C. elegans* furin orthologues for full toxicity (Thacker and Rose, 2000; Salzberg et al, 2014). Proteolytic activation, glycolipid binding and structure are features that this fungal protein toxin shares with bacterial AB toxins.

Finally, several features hint at a role of metal ions for DUF domain activity. A putative Zn^2+^-binding site formed by Cys residues 514, 515, and 550 was detected at the interface between BTF domain 4 and the DUF domain (Fig. 2B). A similar Cys arrangement can be found in, e.g., cytidine deaminases (Cys65, Cys99, and Cys102; PDB ID: 2FR6, Teh et al, 2006) (Appendix Fig. S6). Here, zinc plays a catalytic role, whereas the zinc ion embedded in BTF domain 4 likely provides structural stability or functional regulation (Maret, 2005; Maret and Li, 2009). The three cysteines are generally well-conserved among putative members of the CCTX family (Appendix Fig. S1D). Interestingly, however, they are not conserved in CCTX1 and CCTX3 (Appendix Fig. S1D). More intriguingly, the substitution of Thr739 with Ala leads to a sharp decrease in toxicity (Fig. 4C). Thr739 lies at the bottom of a solvent-exposed pocket, placed at the base of the DUF domain (Appendix Fig. S11A,D) and is mostly lined by negatively charged amino acids (Glu786 and aspartates from the DDDxL motif) - an ideal binding site for divalent cations. Metal-binding has also been found to be essential for the activity of other chimerolectins/toxins, e.g., MOA and PSL1a (Cordara et al, 2011; Wohlschlager et al, 2011; Cordara et al, 2016; Cordara et al, 2017; Manna et al, 2020).

### Concluding remarks

We provide an in-depth investigation of CCTX2, the holotype of a new family of fungal chimerolectins/AB toxins. While CCTX2 undergoes the same GSL-mediated internalization and retrograde trafficking as many plant and bacterial AB toxins, it reveals a unique domain topology and effector domain. This domain adopts an unusual protein fold that is only vaguely similar to a few other structures deposited in the PDB. Further investigation will be needed to identify the biochemical function of this domain and the detailed molecular mechanism of this protein toxin.

## Methods

**Table:** Reagents and tools table

Reagent/resource	Reference or source	Identifier or catalog number
**Experimental models**		
*Escherichia coli* strains	Caenorhabditis Genetics Center (CGC), New England Biolabs, Sigma-Aldrich, Merck, Thermo Fisher Scientific	See Appendix Table S10
*Caenorhabditis elegans* strains	CGC	See Appendix Table S10
**Recombinant DNA**		
pET22	Sigma-Aldrich Merck (Novagen)	
pET24	Sigma-Aldrich Merck (Novagen)	
**Antibodies**		
Rabbit anti-CCTX2 polyclonal serum	Seramun Diagnostica	Custom service
Goat-anti-Rabbit IgG-HRP	BioRad	
**Oligonucleotides and other sequence-based reagents**		
PCR primers	Microsynth	See Appendix Table S11
**Chemicals, enzymes, and other reagents**		
Nucleospin Plasmid Kit	Macherey-Nagel	
Nucleobond Xtra Midi Kit	Macherey-Nagel	
NEBuilder HiFi DNA Assembly kit	New England Biolabs	
Q5 High-Fidelity DNA Polymerase	New England Biolabs	
Phusion DNA Polymerase	New England Biolabs	
Restriction enzymes	Thermo Fisher Scientific, New England Biolabs	na
Transcriptor Universal cDNA Master Kit	Roche	
Recombinant Kex2p	PreproTech	
Ni-NTA beads	Macherey-Nagel	
Sepharose CL 6B	GE Healthcare Life Sciences	
Immobilized D-galactose gel	Thermo Fisher Scientific	
HiTrap desalting column	Cytiva	
Superdex 200 Increase 10/300 GL	Cytiva	
EZ-Link®-sulfo-NHS-biotin kit	Thermo Fisher Scientific	
Alexa Fluor 488	Thermo Fisher Scientific	
TAMRA	Thermo Fisher Scientific	
Vivaspin 20 Centrifugal Filter Units 10 K Molecular Weight Cut Off (MWCO)	VWR	
Sep-Pak cartridge	Merck Millipore	
**Software**		
ZEN system imaging software	Zeiss	
Fiji/ImageJ	Open source	
**Other**		

### Strains and cultivation conditions

*Escherichia coli* strains DH5α and Turbo (New England Biolabs Inc.) were used for cloning and plasmid amplification, while strains BL21(DE3) and C41(DE3) (Sigma) were used for protein production. *E. coli* strains were cultivated in Luria broth (LB) medium, using well-known protocols described, e.g., in Sambrook et al (Sambrook and Russell, 2001). *E. coli* strain OP50 was used as a food source for *C. elegans* (Stiernagle, 2006). The Bristol isolate N2 was used as the wild-type *C*. *elegans* strain; *C. elegans* strains were propagated on nematode growth medium (NGM) as previously described (Stiernagle, 2006). All the *C. elegans* strains included in this study were obtained from *Caenorhabditis* Genetics Center (CGC; University of Minnesota) and are listed in Appendix Table S10.

### Cloning and site-directed mutagenesis

The full-length coding sequences for CCTX2, CCTX1 and CCTX3 were amplified from *Coprinopsis cinerea* AmutBmut fruiting body (CCTX2, CCTX1) and vegetative mycelium cDNA (CCTX3), using primer pairs CCTX1for_His_NdeI/CCTX1rev_NotI (CCTX1), CCTX2for_NdeI/CCTX2rev_NotI (CCTX2), and CCTX3for_His_NdeI/CCTX3rev_NotI (CCTX3), respectively (Appendix Table S11). cDNA synthesis was performed using the Transcriptor Universal cDNA Master kit (Roche) and Phusion DNA polymerase (NEB) as described in Plaza et al (Plaza et al, 2014). The resulting PCR amplicons were cloned into the expression vector pET24b (Novagen) using *Nde*I (Thermo Scientific) and *Not*I (Thermo Scientific) restriction sites. The list of constructs used in this work and their use is reported in Appendix Table S7.

#### Constructs for functional studies

The pET24_CCTX2 plasmid served as the template for the construction of N- and C-terminal truncations of the CCTX2 coding region, including the deletion of the C-terminal HSEL sequon, and for the site-directed mutagenesis of specific residues to alanine. Constructs pET24_CCTX2∆N (lacking residues 2–303) and pET24_CCTX2∆C (lacking residues 570–787), both with an N-terminal His_8_ tag, were generated using primer pairs CCTX2∆Nfor_His_NdeI/CCTX2rev_NotI and CCTX2for_His_NdeI/CCTX2∆C_rev_NotI, respectively. The pET24-CCTX2ΔHSEL construct was cloned using primer pair CCTX2for_NdeI/CCTX2_∆HSEL_rev_NotI (Appendix Table S11); the corresponding PCR products were cloned into the pET24b expression vector using *Nde*I and *Not*I restriction sites. Site-directed mutagenesis of pET24-CCTX2 was performed by overlap extension PCR using Phusion DNA polymerase and a combination of CCTX2for_NdeI, CCTX2rev_NotI, and mutagenesis primers with the respective single, double, and triple mutations. All plasmids were amplified in *E. coli* strain DH5α, purified using mini- and midi-preparation kits (Macherey-Nagel), and verified by Sanger sequencing (Microsynth, Switzerland). Plasmids carrying the correct mutations were then transformed into *E. coli* strain BL21(DE3) for protein production and purification.

#### Constructs for structural studies

A different expression vector was generated for structural studies, carrying CCTX2 fused to a tobacco etch virus (TEV) protease-cleavable His_8_ tag. Starting from the pET24_CCTX2 construct, the *cctx2* gene was subcloned into the pET22b(+) expression vector (Novagen) to generate the pET22b(+)-8H-TEV-CCTX2 construct (Appendix Table S7). The cloning was carried out using the NEBuilder HiFi DNA Assembly kit (New England Biolabs), following guidelines from the manufacturer. Cloning primer pairs for the gene (CCTX2_fwd/CCTX2_rev; Appendix Table S11) and the vector (pET22_fwd/pET22_rev; Appendix Table S9) were designed using the NEBuilder Assembly tool (https://nebuilder.neb.com). PCR was performed using Q5 High-Fidelity DNA Polymerase (New England Biolabs Inc.) following the protocol provided by the manufacturer. Template DNA was degraded using endonuclease *Dpn*I (Thermo Scientific), and assembly of the pET22b(+)-8H-TEV-CCTX2 vector was carried out using the HiFi DNA Assembly Master Mix (New England Biolabs Inc.). Turbo *E. coli* cells (New England Biolabs Inc.) were transformed using the assembly digest and grown overnight (o/n) at 37 °C on LB-agar plates, complemented with 100 μg/mL ampicillin. Single colonies were used to inoculate 5 mL LB media cultures supplemented with 100 μg/mL ampicillin; DNA extraction and purification were performed using the Nucleospin Plasmid kit (Macherey-Nagel). Constructs were verified by Sanger sequencing (Eurofins Genomics GmbH, Germany). Clones carrying plasmids with the correct sequence were amplified by o/n growth at 37 °C in 200 mL LB cultures, supplemented with 100 μg/mL ampicillin. DNA extraction and purification for long-term storage were performed using the NucleoBond Xtra Midi kit (Macherey-Nagel).

### Protein production and purification

#### Protein for functional studies

Constructs were transformed into *E. coli* BL21(DE3) for protein production and purification. *E. coli* BL21(DE3) transformants were grown in LB medium to an optical density at 600 nm (OD_600_) ≈ 0.7 at 37 °C. Cultures were put on ice for 10–15 min before protein production was induced with 0.5 mM IPTG (isopropyl β-D-1-thiogalactopyranoside) for 16 h at 20 °C under constant shaking at 120 rotation per minute (rpm) (Multitron incubator-shaker, Infors HT). All proteins were tested for solubility according to the method described by Künzler et al (Künzler et al, 2010). Bacteria were harvested by centrifugation, and the pellets were resuspended in 20 mL of 20 mM HEPES, 500 mM NaCl, pH 7.5, and lysed using a French press (Avantec). Thereafter, the lysates were centrifuged at 16,000 rpm (Sorvall SS34 rotor) for 30 min to remove cell debris. The resulting supernatant, containing the soluble whole protein fraction, was incubated for 1 h at 4 °C with Sepharose™ CL 6B (GE Healthcare Life Sciences) beads or Ni-NTA beads (Macherey-Nagel), for untagged and His-tagged proteins, respectively. Proteins were batch eluted from Sepharose™ CL 6B or Ni-NTA beads by gravity flow using lysis buffer supplemented with either 200 mM lactose or 250 mM imidazole, respectively. Proteins were desalted on a PD-10 column (GE Healthcare Life Sciences) and the buffer exchanged for 20 mM HEPES and 150 mM NaCl, pH 7.5. MOA, *Coprinopsis cinerea* galectin 2 (CGL2) and *Coprinopsis cinerea* lectin 2 (CCL2) were expressed and purified as described previously (Butschi et al, 2010; Wohlschlager et al, 2011; Schubert et al, 2012).

#### Protein for structural studies

*E. coli* C41 (DE3) cells were transformed with pET22b(+) following a standard protocol, like the one described, e.g., in Sambrook et al (Sambrook and Russell, 2001). C41 (DE3) was selected among a panel of several *E*. *coli* strains, as it minimized CCTX2 cleavage by endogenous proteases. Glycerol stocks were prepared from successful transformants, cultured over day (o/d) at 37 °C in LB supplemented with 100 µg/mL ampicillin and stored at −80 °C. In all, 2 mL precultures were inoculated with multiple colonies, taken from LB-agar plates streaked with glycerol stocks, and grown o/d at 37 °C, shaking at 120 rpm (Multitron incubator-shaker, Infors HT). In total, 50 mL LB medium cultures containing 100 µg/mL ampicillin were inoculated with the 2 mL precultures and grown at 37 °C, 120 rpm for 16 h. The 50 mL cultures were then used to inoculate 1 L of LB medium complemented with 100 µg/mL ampicillin to an optical density at 600 nm (OD_600_) of 0.1, and incubated at 37 °C, 120 rpm. When OD_600_ reached ≈0.6, the culture was cooled down in iced water for 10 to 15 min. Expression of the gene was induced with 0.5 mM IPTG for 18–20 h at 20 °C, shaking at 120 rpm (Multitron incubator-shaker, Infors HT).

Cells were harvested at 4000×*g*, 4 °C for 30 min and resuspended in 5 mL of lysis buffer per gram of cell paste. The lysis buffer was prepared to a final concentration of 50 mM HEPES, 200 mM NaCl, 2 mM EDTA, 5 mM DTT; the pH was adjusted to pH 7.5. The buffer was complemented with c*O*mplete EDTA-free protease Inhibitor cocktail (Roche Life Science) to the working concentration recommended by the manufacturer. Cells were lysed using an ultrasound homogenizer (Qsonica Q500) at 20% amplitude for 1 min (10 s cycles: 3 s “on”, 7 s “off”). Cell lysates were clarified at 40,000×*g*, 4 °C for 40 min. The supernatants were recovered and directly used for purification.

Galactose-affinity chromatography was carried out at RT on an ÄKTA Start (GE Life Sciences), using Immobilized D-galactose gel (Thermo Scientific) packed into a 5 mL Tricorn 10/50 column (GE Life Sciences). The affinity column was equilibrated with loading buffer (20 mM HEPES, 500 mM NaCl, pH 7.5), loaded with cell lysate, and washed with six column volumes (CV) of loading buffer. Bound protein was eluted with ten CVs of elution buffer (20 mM HEPES, 500 mM NaCl, 1 M D-galactose, pH 7.5). Fraction content was assessed by SDS-PAGE; fractions containing CCTX2 were pooled and concentrated by ultrafiltration at 3500×*g*, 4 °C using Vivaspin® 20 Centrifugal Filter Units 10 K Molecular Weight Cut Off (MWCO) (VWR). Size exclusion chromatography (SEC) was performed to ensure sample purity and monodispersity. A Superdex 200 Increase 10/300 GL (Cytiva) gel-filtration column was equilibrated with SEC buffer (50 mM HEPES, 150 mM NaCl, 100 mM D-galactose; pH adjusted to 7.5) and loaded with the concentrated sample from ultrafiltration. The content of eluted fractions was assessed by SDS-PAGE; fractions containing pure CCTX2 were pooled and loaded onto a 5 mL HiTrap desalting column (Cytiva), previously equilibrated with dialysis buffer (20 mM HEPES, pH 7.5). The dialyzed protein was kept at 4 °C until its use for structural studies.

### Protein labeling with Biotin, Alexa Fluor®488, and TAMRA

For thin-layer chromatography (TLC), glycan array analysis, and *C. elegans* in vivo imaging, proteins were purified as described above (‘Protein purification for functional studies’) and biotinylated with the EZ-Link®-sulfo-NHS-biotin kit (Thermo Scientific, USA). The proteins were labeled following the manufacturer's instructions, using a 20-fold molar excess of labeling agent. Protein labeling with Alexa®488 (Thermo Scientific, USA) and 5-carboxytetramethylrhodamine (TAMRA; Thermo Scientific, USA) was performed as described previously (Bleuler-Martínez et al, 2011; Wohlschlager et al, 2011; Stutz et al, 2015).

### Preparation and analysis of glycosphingolipids from *C. elegans*

Total lipids were extracted, and upper phase lipids containing glycosphingolipids (GSLs) were separated by thin-layer chromatography (TLC) according to Barrows et al (Barrows et al, 2006; Wohlschlager et al, 2011). The binding of separated lipids to CCTX2 was assessed by overlaying the TLC plates with biotinylated CCTX2 and HRP-coupled avidin. The biotinylated GSL-binding chimerolectin MOA (Wohlschlager et al, 2011) and the N-terminally truncated version of CCTX2 (CCTX2∆N) were used as controls. GSLs were extracted from *C. elegans* wild-type N2 strain as well as from the mutant strains *bre-2, bre-3, bre-4*, and *bre-5* and separated using TLC, as described by Barrows et al (Barrows et al, 2006). In brief, L4-staged larvae from each strain were collected and washed three times in 10 ml H_2_0. The resulting pellet was flash-frozen in liquid nitrogen, and worm pellets were sonicated to break the worm cuticle. 1 volume of homogenized worm pellet was mixed first with methanol and then with chloroform to a final ratio of 4:8:3 (chloroform:methanol:water). Samples were incubated for 2 h at 37 °C and afterward centrifuged 5 min at 1400×*g*. Supernatant was transferred into a new glass vial. In all, 0.147 volumes of water were added to the supernatant. Samples were centrifuged again as above to achieve phase separation. The upper phase (containing the glycolipids) was transferred onto a Sep-Pak cartridge (Millipore) for reversed-phase chromatography. For TLC overlay analysis, 150 nM biotinylated MOA, CCTX2, or CCTX2∆N were used following the protocol described by Wohlschlager et al (Wohlschlager et al, 2011).

### Glycan array analysis

Alexa Fluor 488-labeled full length and N-terminally truncated CCTX2 was used to probe the mammalian plate glycan array v5.1 offered by the Consortium for Functional Glycomics (CFG, Emory University, Atlanta). The screening of the array was performed at lectin concentrations of 200, 50, 5 and 2 μg/mL (full-length CCTX2) and 200 μg/mL (CCTX2ΔN). The data is available as Supplementary Datasets EV1–5.

### *C. elegans* toxicity assay in liquid medium with purified protein

*C. elegans* toxicity assays were carried out as previously described (Künzler et al, 2010; Stutz et al, 2015) with the following modifications: Assays were performed in 96-well plates, with ~30 L1 staged larvae. Larvae were incubated in 100 µl 1× phosphate-buffered saline (PBS; pH 7.4) containing *E. coli* OP50 OD_600_ = 2 and the indicated protein concentrations for 72 h at 20 °C. Larval development into the L4 stage or adulthood was scored as full development.

### Microscopy of *C. elegans*

*C. elegans* strains were grown into L4 stage and fed with 150 μg/ml of 5-TAMRA-labeled MOA or CCTX2 or with 500 μg/ml of 5-TAMRA-labeled CCL2 for the indicated time, before washing 3x with 1× PBS to remove excess protein. Worms were immobilized using 10 mM levamisole and mounted on a 3% w/v agarose pad. Fluorescence images were taken using a Zeiss LSM 780 upright confocal microscope. Image acquisition was performed using the ZEN system imaging software (Zeiss, Germany). Image analysis was performed using the software Fiji (Schindelin et al, 2012), a distribution of the open-source imaging software ImageJ (Schneider et al, 2012).

### Kex2p proteolysis assay

In total, 100 μg of CCTX2 were incubated at 37 °C overnight (o/n) with 10 μg recombinant Kex2p from *Saccharomyces cerevisiae* (PeproTech). Samples from the reactions were collected, diluted 1:10, and separated by SDS-PAGE. Gel bands were excised and identified by tryptic digest and peptidomics (Functional Genomics Center Zürich). The remainder of the reactions was incubated with Sepharose^TM^ CL 6B or Ni-NTA beads for 1 h at RT on a rotating wheel. Thereafter, the slurry was loaded onto Mobicol spin columns (MoBiTec, Germany) and the flow-through was collected. The beads were washed with 50 column volumes of 20 mM HEPES, 150 mM NaCl, pH 7.5, and boiled in Laemmli buffer (Laemmli, 1970). The eluates were subjected to an immunoblot analysis using a polyclonal rabbit antiserum raised against recombinant CCTX2 (Seramun Diagnostica; Appendix Fig. S10C).

### Sequence analysis, phylogeny, and structure prediction

Domain predictions were performed using the SMART software (Simple Modular Architecture Research Tool, biobyte solutions GmbH, Germany; http://smart.embl-heidelberg.de). CLUSTALW amino acid sequence alignments were performed using the Pole Bio-Informatique Lyonnais (PBIL)-based software NPS@CLUSTALW (Combet et al, 2000) (Network Protein Sequence @nalysis, Lyon, France; https://npsa-prabi.ibcp.fr/NPSA/npsa_clustalw.html). The multiple sequence alignment of the three *C. cinerea* paralogues CCTX1, CCTX2, and CCTX3, as well as their fungal full-length homologs, was done using the MUltiple Sequence Comparison by Log-Expectation (MUSCLE) algorithm (Edgar, 2004). The alignment was visualized in Unipro UGENE software (Okonechnikov et al, 2012) and manually trimmed (Appendix Fig. S1D). The phylogenetic tree was constructed using the RAxML-ng algorithm (Kozlov et al, 2019), which implements the maximum-likelihood (ML) optimality criterion, setting 500 bootstrap replicates under a Jones-Taylor-Thornton matrix. The phylogenetic tree with the highest bootstrap support was visualized (Appendix Fig. 1B) using ITOL (Letunic and Bork, 2024). In silico structure prediction for CCTX2 was initially carried out using the Robetta server (Kim et al, 2004) (https://robetta.bakerlab.org), based on the Rosetta simulation framework (Baek et al, 2021), applying the ‘comparative modeling’ protocol and automatic template selection (Song et al, 2013b). Later on, a model was generated using AlphaFold 3 (Abramson et al, 2024). Homology models of CCTX1 and CCTX3, used for domain boundary prediction, were generated using the Robetta server (Kim et al, 2004) (https://robetta.bakerlab.org), applying the “comparative modeling” protocol and uploading the cryo-EM structure of CCTX2 as a template.

### Statistical analysis

No inferential statistics were applied; results are presented as raw data counts.

### Cryo-EM grid preparation and data collection

Cryo-EM grids were prepared at the SciLife lab cryo-EM Swedish National facility, Umeå node (Sweden) according to standard procedures described, e.g., Passmore and Russo (Passmore and Russo, 2016). Quantifoil R2/1 300 mesh carbon grids (Quantifoil Micro Tools, Germany) were glow-discharged at 15 mA for 30 s with a Pelco easiGlow glow-discharge cleaning system (Ted Pella, Inc.). In total, 4 μL of 1 mg/mL CCTX2 was applied to the grids using a Vitrobot Mark IV (Thermo Scientific) equilibrated at 4 °C, 100% humidity. In total, 7500 movies were collected on a FEI Titan Krios (Thermo Scientific) operating at 300 kV and equipped with a Gatan K2 camera, at a magnification of ×80,000. The total electron exposure of 59.5 e^-^/Å^2^ was distributed over 40 frames. Nominal defocus range was −1.5 to −3.0 μm in 0.3-μm steps. Data collection statistics are reported in Appendix Table S2.

### Image processing and model building

The dataset was processed using *CryoSPARC* v3.1 (Punjani et al, 2017) as described in Appendix Fig. S4. In brief, patch motion correction and contrast transfer function (CTF) estimation were performed. After manual particle picking, homogenous refinement was carried out to generate a volume for template-based particle picking, generating a stack of 1.8 million particles. After three rounds of 2D classification, 641,000 particles remained with a final extraction at a box size of 480 pixels and downsampled to 256 pixels. These particles were used for ab initio reconstruction, where the best class was chosen for heterogeneous refinement. A final stack of 204,000 particles was selected for ‘Local refinement’ in *CryoSPARC*. Global resolution was determined based on the gold-standard Fourier Shell Correlation (FSC) 0.143 cut-off using two independently refined half-maps (Appendix Fig. S4B). Local resolution estimates were calculated using CryoSPARC (Appendix Fig. S4C). The final map was locally filtered and sharpened using the overall *B*-factor determined during the refinement step.

Predictive models were generated using the Robetta server (Kim et al, 2004) for all the CCTX2 domains, using either template-based (BTF domains 1–4) or ab initio (C-terminal domain) protocols. Directly fitting the models into the reconstructed volume proved impossible, due to the strong structural homology among the BTF domains (plus the presence of a pseudo-threefold axis) and the poor quality of the C-terminal domain prediction; automated protein chain building was not successful either. However, a polyalanine trace, manually built using *Coot* (Casañal et al, 2020), a component of the *CCP4* software suite (Agirre et al, 2023), revealed the correct domain topology. The first three BTF domains were easily docked onto their respective polyAla trace, whereas the fourth domain had to be manually aligned in PyMOL (Schrödinger, Inc.), only relying on the backbone segments built into the discontinuous Coulomb potential density (Appendix Fig. S3A). Poorly predicted segments were removed from the model of the C-terminal domain, keeping only a core region (residues 619-740; Appendix Fig. S3B,C). The CCTX2 model was completed using *Coot*, by accurately fitting the models into the cryo-EM volume and building missing residues; an in silico model generated using AlphaFold 3 (Abramson et al, 2024) was used to improve areas with poor density fit. Refinement was carried out by alternating cycles of real-space building using *Coot* (Casañal et al, 2020) and real-space refinement with *phenix.refine*, a component of the *Phenix* software suite for structural biology (Liebschner et al, 2019). The final model includes the entire CCTX2 chain (residues 1–787); the C-terminal amino acid residues from the His_8_-TEV tag were poorly defined and were not modeled. Similarly, galactose molecules at the putative sugar-binding sites of the first (subdomains β and γ) and second (subdomain α) BTF domains were not modeled due to poor fit density. Refinement statistics are reported in Appendix Table S2: although the model displays a remarkably high number of Ramachandran outliers (8, or about the 1%, evaluated with RAMPAGE (Lovell et al, 2003)), their geometry is generally close to the allowed regions of the plot. All structural illustrations were prepared using PyMOL (Schrödinger, Inc.).

## Supplementary information

Appendix

Peer Review File

Dataset EV1

Dataset EV2

Dataset EV3

Dataset EV4

Dataset EV5

Figure Source Data Checklist

Source data Fig. 1

Source data Fig. 4

Source data Fig. 5

Source data Fig. 6

## Data Availability

Source data of the main figures and additional figures, tables, and datasets are supplied as supplementary information in this paper. CCTX2 structural data were deposited at the Electron Microscopy Data Bank (EMDB) under the ID EMD-54430; coordinates were deposited at the Protein Data Bank (PDB) under the ID 9S11. The source data of this paper are collected in the following database record: biostudies:S-SCDT-10_1038-S44318-026-00812-1.
