# Supplementary material for: Structure and function of a fungal AB toxin-like chimerolectin involved in anti-nematode defense
Source: EMBO J. 2026 May 26;45(13):4766–86. doi: 10.1038/s44318-026-00812-1 (PMC13324453; doi:10.1038/s44318-026-00812-1)
Supplement: Supplementary file 1 — Appendix [file 44318_2026_812_MOESM1_ESM.pdf]

Appendix for

Structure and function of a fungal AB toxin-like chimerolectin involved in anti-nematode defense

Stefanie S. Schmieder[1*‖], Gabriele Cordara[2*#], Flore Kersten[2,3], Kevin Steiner[1&], Clara H. Samim[2], David F. Plaza [1], Ahmad Ali-Ahmad[, 3], Andreas Boeggild[4], Jesper L. Karlsen[4], Blanka O. Sokolowska[1], Thomas Boesen[4], Ute Krengel[2#] and Markus Künzler[1#]

[1]Institute of Microbiology, Department of Biology, ETH Zürich, Zürich, Switzerland

[2]Department of Chemistry, University of Oslo, Norway

[3]Centre for Molecular Medicine Norway, University of Oslo, Norway

[4]Department of Molecular Biology and Genetics, Aarhus University, Denmark

*Contributed equally to the work

‖present address: Division of Gastroenterology, Boston Children's Hospital, Harvard Medical School, Boston, USA

[&]present address: Institute of Medical Virology, University of Zürich, Zürich, Switzerland

[#]Corresponding authors: gabriele.cordara@kjemi.uio.no and ute.krengel@kjemi.uio.no (structure), mkuenzle@ethz.ch (biochemistry and function)

Table of content

**Appendix item**	**Page**
Figure S1. Differential expression, toxicity and sequence alignment of CCTX2 paralogues	2
Figure S2. Toxicity profile of MOA and CCL2 in *C. elegans* endocytosis mutants	4
Figure S3. Model building and comparison of BTF domains	5
Figure S4. CCTX2 cryo-EM data processing	7
Figure S5. β-trefoil fold (BTF) domains of CCTX2	9
Figure S6. Putative zinc binding site of CCTX2	11
Figure S7. DUF structural homologues identified by DALI	12
Figure S8. DUF domain core structural homologues identified by DALI	14
Figure S9. Glycan array analysis of CCTX2 and the N-terminally truncated CCTX2ΔN	16
Figure S10. Alanine scanning of conserved residues within the C-terminal DUF domain	17
Figure S11. Structural details of functionally relevant Ala mutations	18
Figure S12. AlphaFold3 model of post-Kex2-cleavage CCTX2	19
Figure S13. Comparison of CCTX2 with the mosquitocidal holotoxin from *Pieris rapae* (MTX)	21
Table S1. List of full-length CCTX2 homologs in the fungal kingdom	22
Table S2. Cryo-EM data collection and model refinement statistics	23
Table S3. Poorly-defined regions in the cryo-EM map	24
Table S4. CCTX paralogue domain boundaries and features outline	25
Table S5. BTF domain homology	26
Table S6. CCTX2 β-trefoil fold (BTF) domains	27
Table S7. DALI - DUF domain (full-length) homology	28
Table S8. DALI - DUF domain *core* homology	29
Table S9. Plasmids generated in this study, and toxicity results	30
Table S10. *Caenorhabditis* spp. and *Escherichia coli* strains used in this study	31
Table S11. Selected primers used in this study	32
References	33

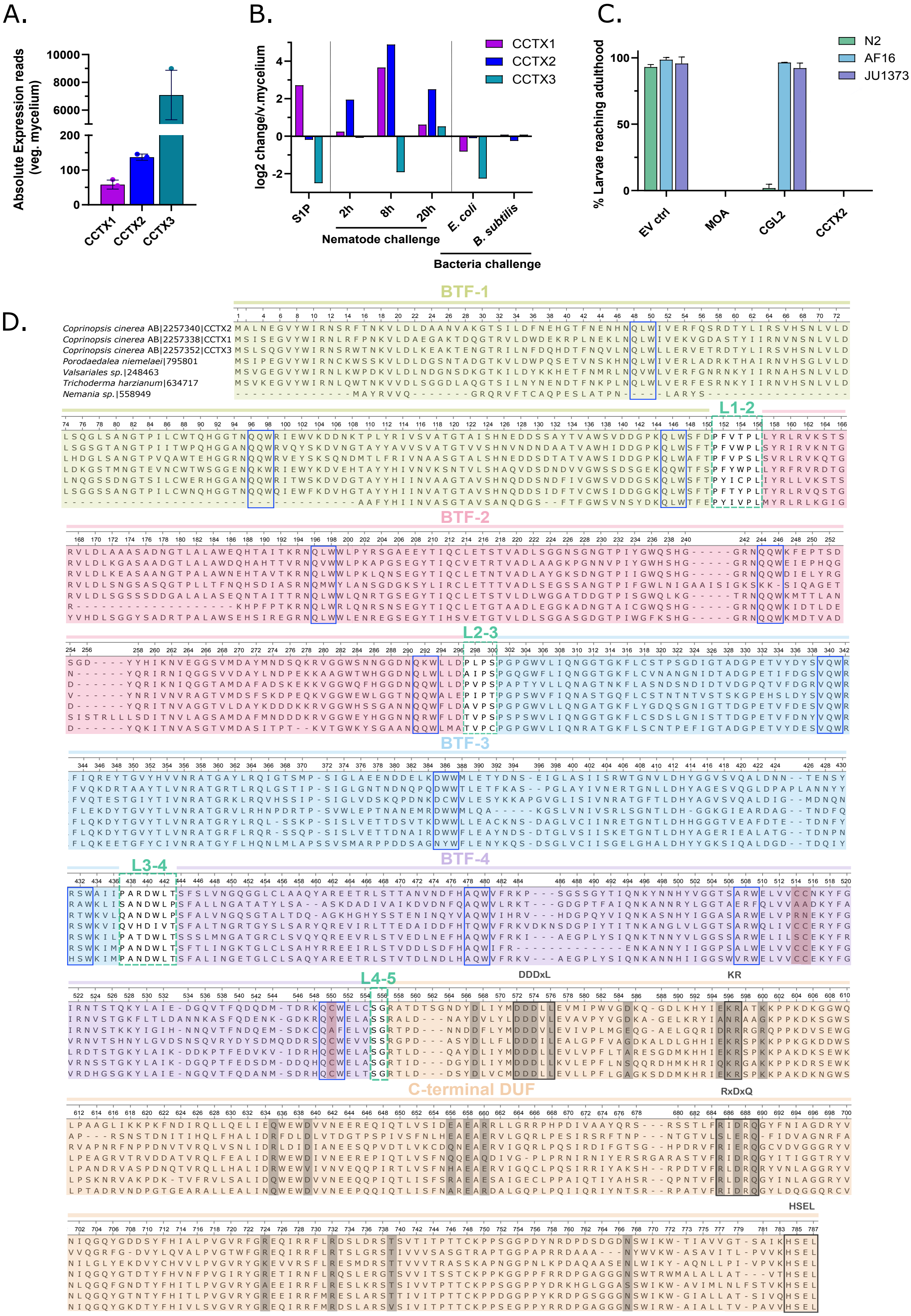

A.
Absolute Expression reads (veg. mycelium)
10000
8000
6000
200
100
0
CCTX1
CCTX2
CCTX3
B.
log2 change/v.mycelium
CCTX1
CCTX2
CCTX3
S1P
2h
8h
20h
Nematode challenge
E. coli
B. subtilis
Bacteria challenge
C.
% Larvae reaching adulthood
N2
AF16
JU1373
EV ctrl
MOA
CGL2
CCTX2
D.
BTF-1
Coprinopsis cinerea AB|2257340|CCTX2
Coprinopsis cinerea AB|2257338|CCTX1
Coprinopsis cinerea AB|2257352|CCTX3
Porodaedalea niemelaei|795801
Valsariales sp.|248463
Trichoderma harzianum|634717
Nemania sp.|558949
L1-2
BTF-2
L2-3
BTF-3
L3-4
BTF-4
DDDxL
KR
L4-5
C-terminal DUF
RxDxQ
HSEL

Appendix Figure S1. Differential expression, toxicity and sequence alignment of CCTX2 paralogues. **A**. Absolute mRNA levels for CCTX1, CCTX2 and CCTX3 in *C. cinerea* vegetative mycelium. Data points with error bars are means of N = 3 biological replicates with standard deviation (SD) (Data ref: Muraguchi et al, 2015). **B.** Differential mRNA expression analysis of CCTX1, CCTX2 and CCTX3 in *C. cinerea* vegetative mycelium vs S1P primordia and vegetative mycelium challenged by fungivorous nematode *A. avenae*, and two bacterial species, *E. coli* and *B. subtilis* (Data ref: Muraguchi et al., 2015; Tayyrov et al, 2018; Kombrink et al, 2019). **C**. Differential toxicity of the chimerolectins CCTX2, MOA and the lectin CGL2 against three bacterivorous *Caenorhabditis* species N2 (*elegans*), AF16 (*briggsae*) and JU1373 (*tropicalis*). Here, genes were overexpressed in *E. coli* and the recombinant bacteria fed to the nematodes. **D.** Sequence alignment of members of the monophyletic clade of fungal full-length homologues including the three paralogues from *C. cinerea* (see complete results of BLAST search in Fig. 1A). The alignment shows that the overall chimeric architecture of *C. cinerea* proteins, consisting of four lectin domains and a C-terminal domain of unknown function, as well as a DDDxL motif, a putative kexin-cleavage site (KR), a RxDxQ motif and a putative ER-retention signal (HSEL) are conserved among these homologues. Putative zinc binding sites, which are conserved among homologues, are indicated by red shading, while conserved residues that were subjected to single substitutions by alanine in CCTX2 for functional analysis (alanine scanning), are highlighted in grey.

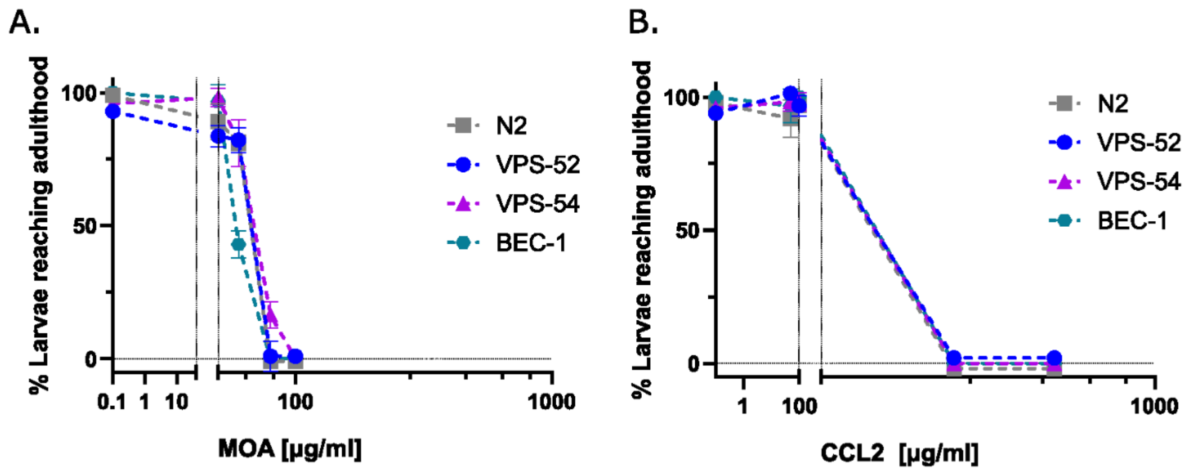

Appendix Figure S2. Toxicity profile of MOA and CCL2 in *C. elegans* endocytosis mutants. Toxicity assay with CCTX2 and different *C. elegans* retrograde trafficking mutants VPS-52 (VC625) and VPS-54 (VC985) bear mutations in the GARP complex subunits VPS-52 and VPS-54, respectively. BEC-1 is required for retromer localization to endosomes. VPS-36 (VC947) exhibits a mutation in the ESCRT II subunit. **A.** Liquid toxicity assay, performed with *C. elegans* retrograde transport mutants using different concentrations of the purified GSL binding lectin MOA. **B.** The same assay was performed with the *N*-glycan binding lectin CCL2 using 50, 100, 250 and 500 µg/ml of protein. Data points with error bars indicate means of N = 4 biological replicates with standard error of the mean (SEM). Overlapping data points were nudged by +/- 2 data units in *y* for better readability.

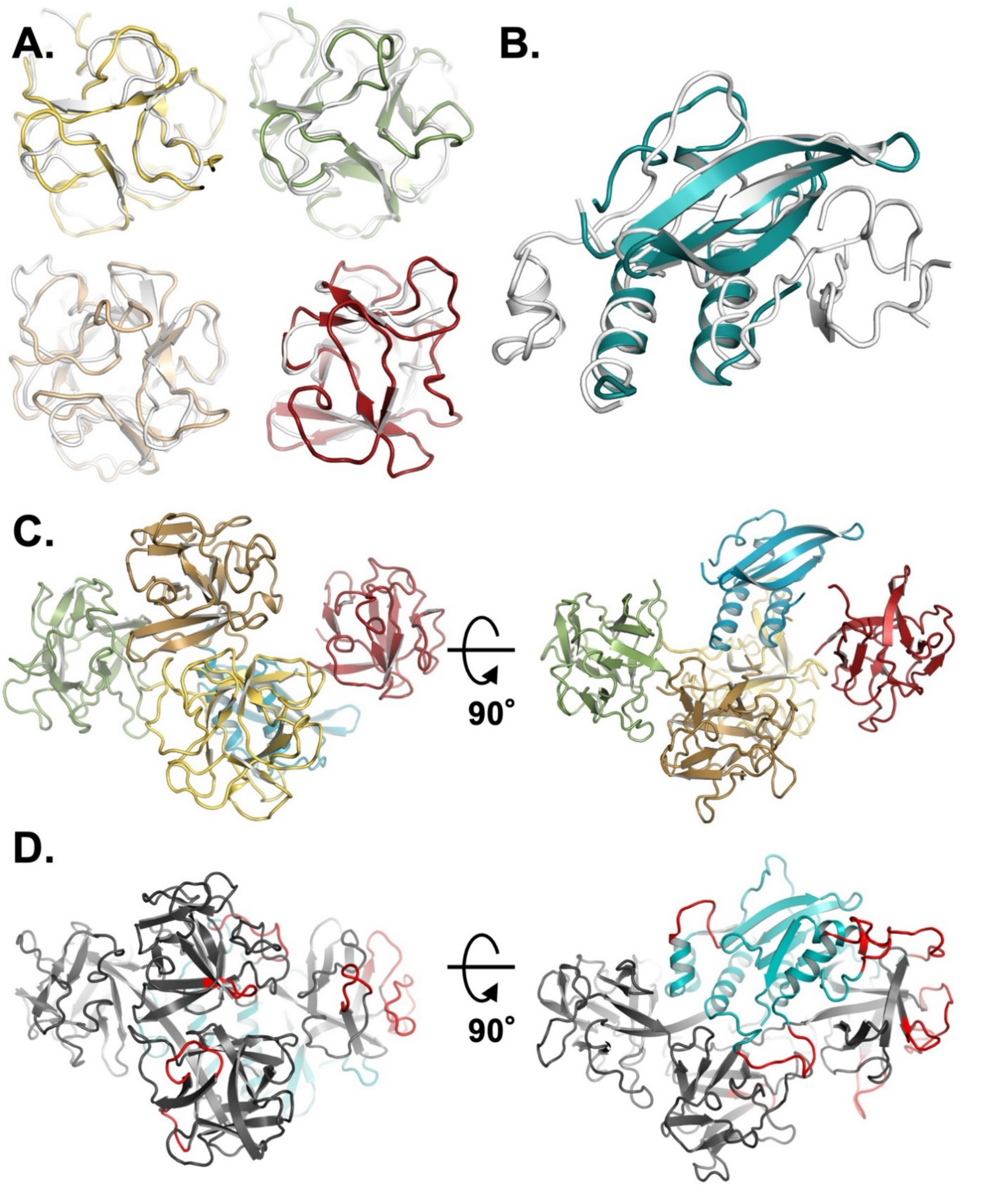

Appendix Figure S3. Model building and comparison of BTF domains. A. Cartoon representation of the structural alignments of the manually-built polyalanine trace (white) and the template-based homology models generated with Robetta (Kim et al, 2004) for domain 1 (yellow), domain 2 (green), domain 3 (wheat) and domain 4 (red). **B.** Cartoon representation of the structural alignment between the manually built polyalanine trace (white) and the core portion (amino acids 619-740) of the model for the C-terminal domain (deep teal). The model was generated using the Robetta server, applying the *ab initio* protocol and removing

all the poorly predicted regions. **C.** Cartoon representation of CCTX2 starting model, after docking all the domain models onto the polyalanine trace. **D.** Final model of CCTX2, with the BTF domains colored in black and the C-terminal DUF domain colored in teal. Highlighted in red are the parts of the model built into partially discontinuous density; boundaries for each region are reported in Appendix Table S3.

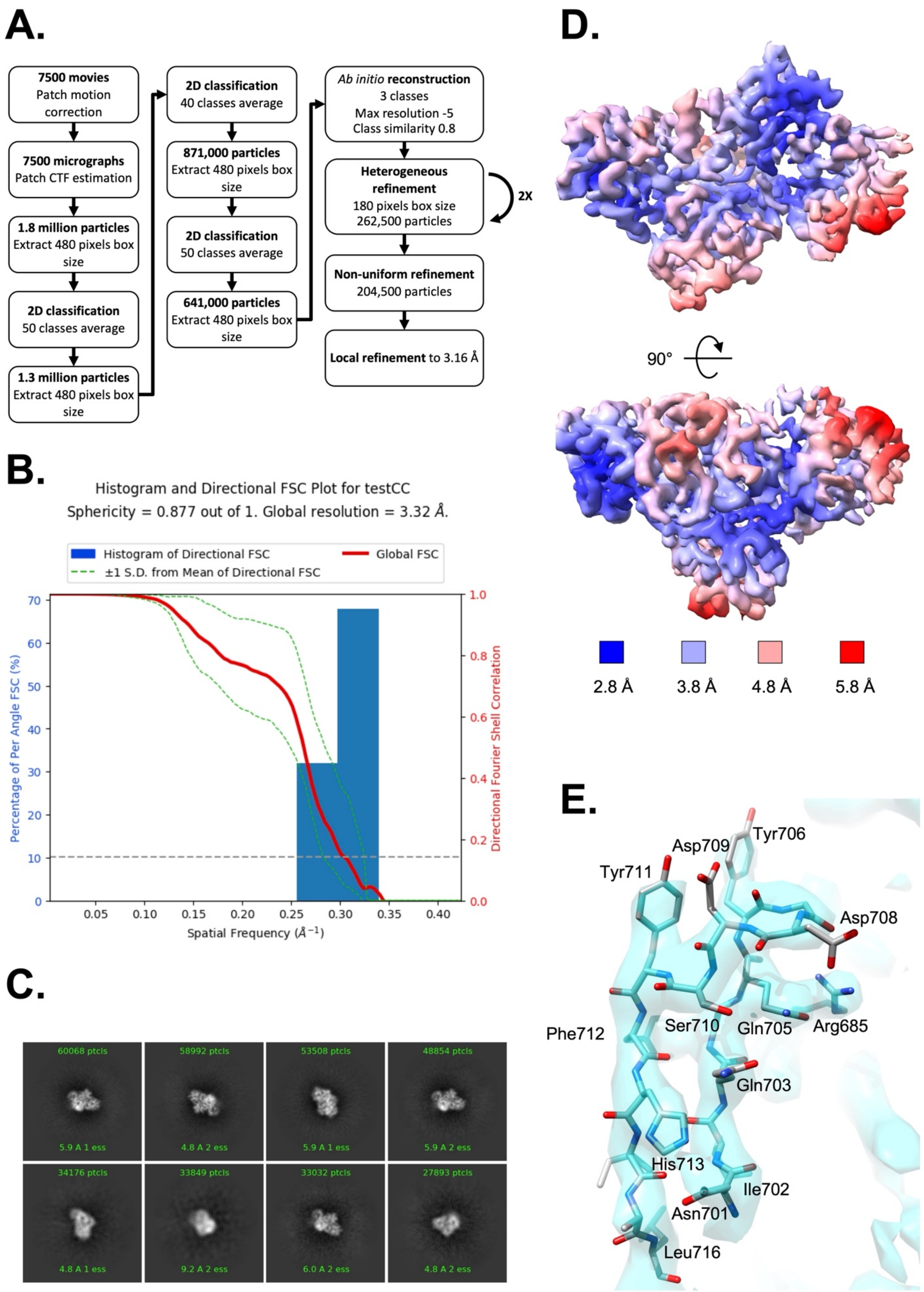
A.
7500 movies
Patch motion correction
7500 micrographs
Patch CTF estimation
1.8 million particles
Extract 480 pixels box size
2D classification
50 classes average
1.3 million particles
Extract 480 pixels box size
2D classification
40 classes average
871,000 particles
Extract 480 pixels box size
2D classification
50 classes average
641,000 particles
Extract 480 pixels box size
Ab initio reconstruction
3 classes
Max resolution -5
Class similarity 0.8
Heterogeneous refinement
180 pixels box size
262,500 particles
2X
Non-uniform refinement
204,500 particles
Local refinement to 3.16 Å
B.
Histogram and Directional FSC Plot for testCC
Sphericity = 0.877 out of 1. Global resolution = 3.32 Å.
Histogram of Directional FSC
Global FSC
±1 S.D. from Mean of Directional FSC
Percentage of Per Angle FSC (%)
Directional Fourier Shell Correlation
Spatial Frequency (Å^-1)
C.
60068 ptcls
58992 ptcls
53508 ptcls
48854 ptcls
5.9 A 1 ess
4.8 A 2 ess
5.9 A 1 ess
5.9 A 2 ess
34176 ptcls
33849 ptcls
33032 ptcls
27893 ptcls
4.8 A 1 ess
9.2 A 2 ess
6.0 A 2 ess
4.8 A 2 ess
D.
90°
2.8 Å
3.8 Å
4.8 Å
5.8 Å
E.
Tyr706
Asp709
Tyr711
Asp708
Phe712
Ser710
Gln705
Arg685
Gln703
His713
Ile702
Asn701
Leu716

Appendix Figure S4. CCTX2 cryo-EM data processing. A. Processing workflow in CryoSPARC, leading to the reconstructed cryo-EM volume used to build the CCTX2 model. **B.** Histogram and directional FSC plot for testCC. **C.** Representative 2D classes for particles extracted from the cryo-EM micrographs for the last volume reconstruction. **D.** Local resolution map. **E.** Example of the quality of the reconstructed Coulomb potential map in the DUF domain.

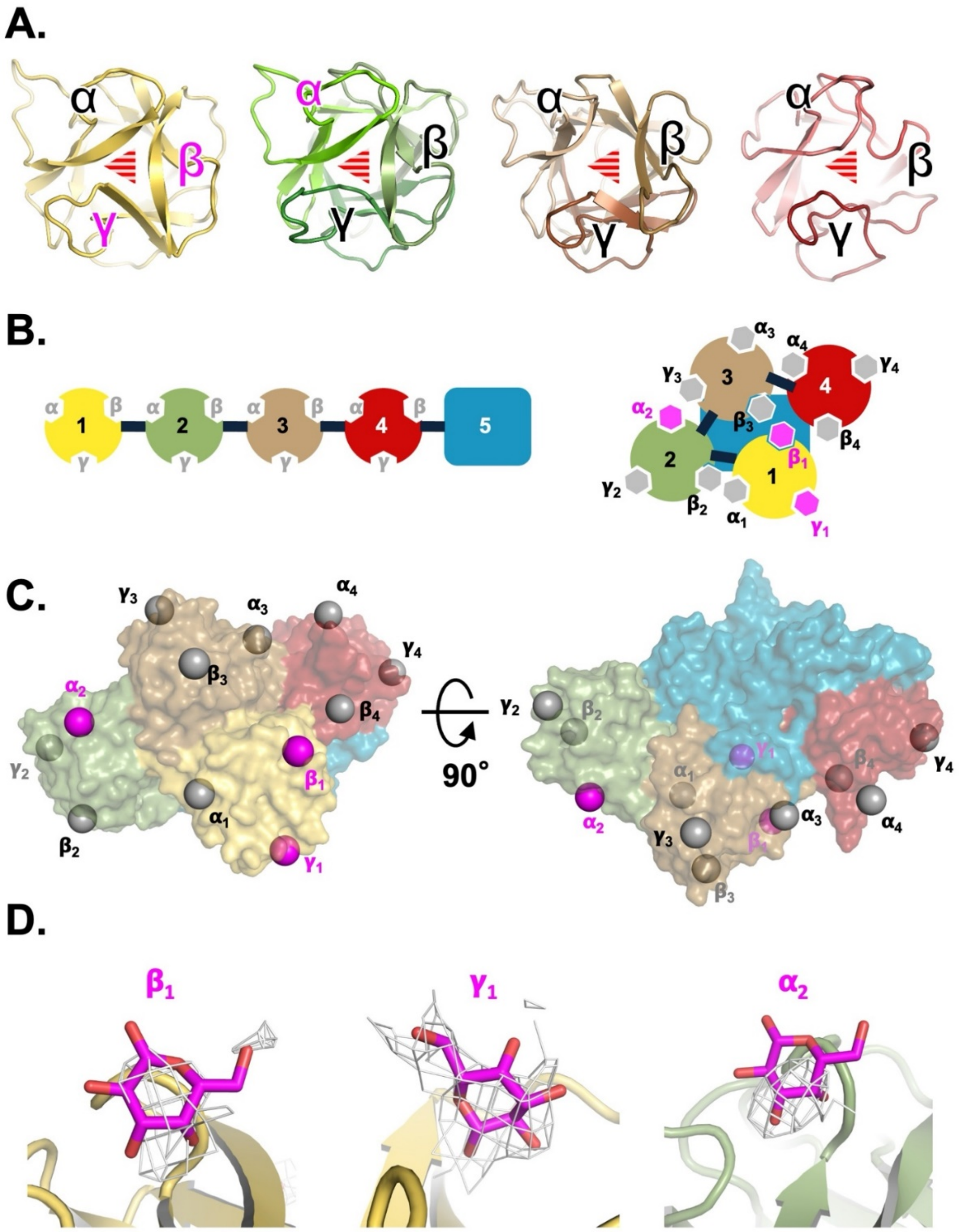

Appendix Figure S5. Beta-trefoil fold (BTF) domains of CCTX2. A. Cartoon representation of BTF domains 1 (yellow), 2 (green), 3 (wheat) and 4 (dark red). α, β and γ subdomains are marked on each BTF domain; subdomains showing ligand density are marked in magenta. Coordinates of BTF domains 2 to 4 are shown superimposed to those of BTF domain 1 (grey) for comparison. The pseudo-threefold symmetry axis associated with the BTF fold is represented by a striped triangle at the center of each domain. **B.** Schematic representation of CCTX2, with the positions of α, β and γ subdomains marked on the BTF domains. The right panel shows a schematic drawing of CCTX2, with the positions of all possible sugar-binding sites marked on the solvent-exposed surface of the BTF domain cradle. Sugar-binding sites with supporting Coulomb potential density are marked in magenta. **C.** Surface representation of CCTX2. Putative carbohydrate-binding sites are marked with a grey-colored sphere, while those displaying

experimental density are marked with a magenta-colored sphere. **D.** Patch of density at the putative sugar binding sites of BTF subdomain β_1, γ_1 (left and central) and α_2 (right) (left unmodelled in the deposited structure).

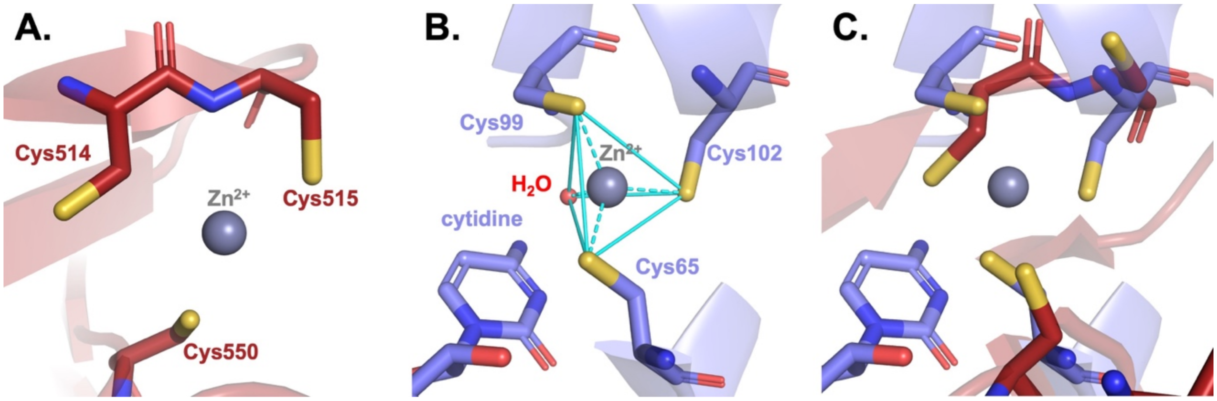

Appendix Figure S6. Putative zinc binding site of CCTX2. A. Zinc binding site of CCTX2 predicted by AlphaFold 3 (Abramson et al, 2024) in a simulation performed in the presence of Zn^{2+}. The predicted site coincides with the zinc binding site inferred from analysis of the cryo-EM structure. **B.** Zinc binding site of murine cytidine deaminase (PDB ID: 2FR6; Teh et al, 2006), showing the tetrahedrally-coordinated Zn^{2+} ion at the catalytic site. **C.** Superposition between the coordinates of cysteine residues involved in zinc coordination in murine cytidine deaminase (PDB ID: 2FR6; Teh et al., 2006) and CCTX2 (this study).

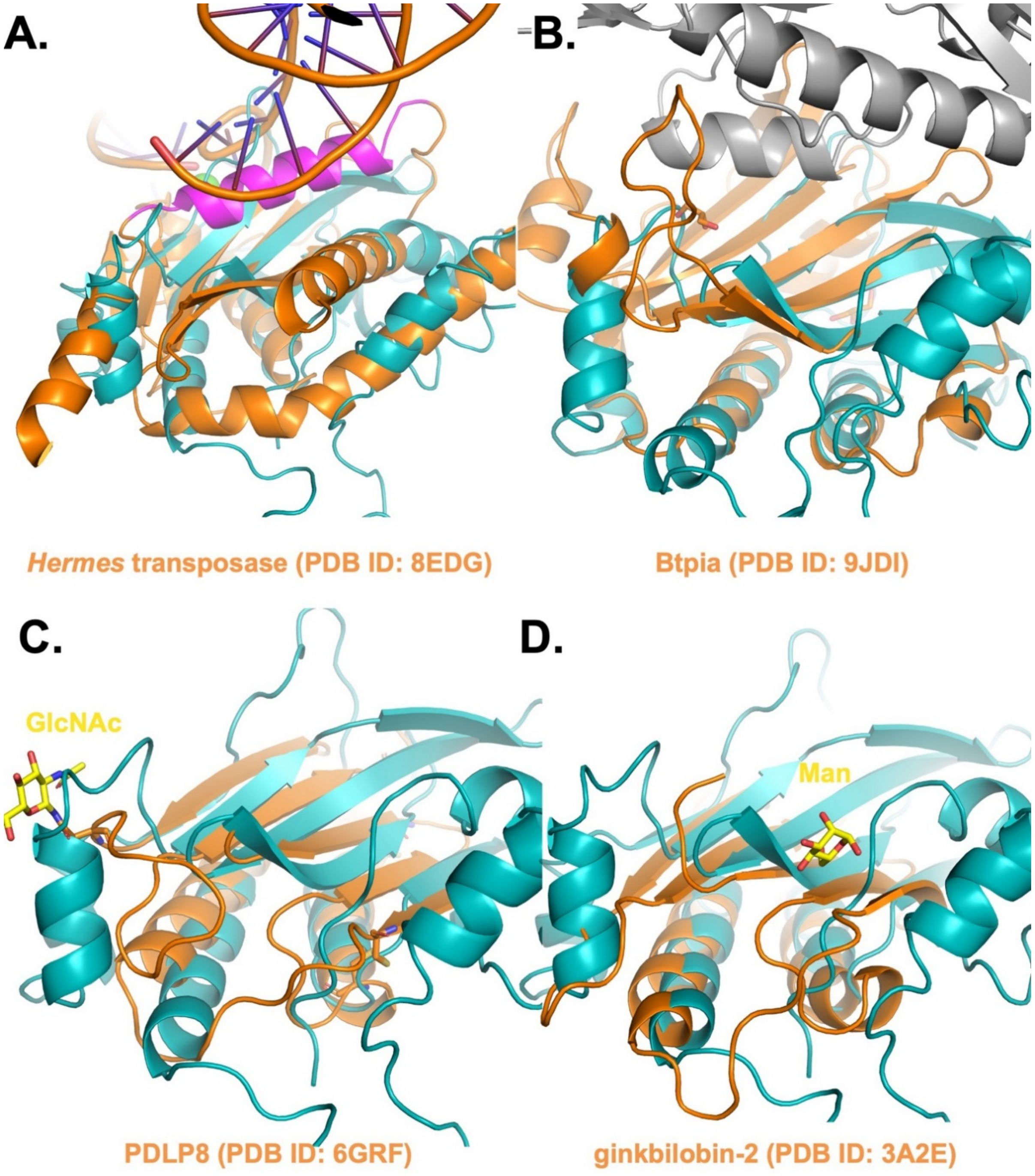

Appendix Figure S7. DUF structural homologues identified by DALI. The figure shows structural alignments of the full-length DUF domain (aa 557-787, teal; this work) with structural homologues identified through a DALI search (orange). **A.** Structural alignment with the RNase-H-like domain of the housefly *Hermes* transposase (PDB ID: 8EDG; Lannes et al, 2023). The RNase H-like unit contacts nucleic acids through an α-helix (magenta) supported by its central β-sheet. **B.** Structural alignment with the inhibitory component of the toxin-antitoxin BtpeA-BtpiA system of *Bacteroides fragilis* (PDB ID: 9JDI; Li et al, 2025). BtpiA (orange) uses the solvent-exposed cleft of its large β-sheet to bind its cognate partner (BtpeA, black). **C.** Structural alignment with the structure of the DUF26 ectodomain of plasmodesmata-located protein 8 (PDLP8) from *Arabidopsis thaliana* (PDB ID: 6GRF; Vaattovaara et al, 2019). The latter is *N*-glycosylated (yellow) on a loop insertion between two β-strands, corresponding to the

second insert of the CCTX2 DUF domain (residues 656-683). **D.** Structural alignment with the antifungal protein ginkbilobin-2 from *Ginkgo biloba* (PDB ID: 3A2E; Miyakawa et al, 2009). The structure of ginkbilobin-2 was solved in complex with mannose (yellow), binding to a solvent-exposed β-sheet that superimposes with a similar folding motif of CCTX2.

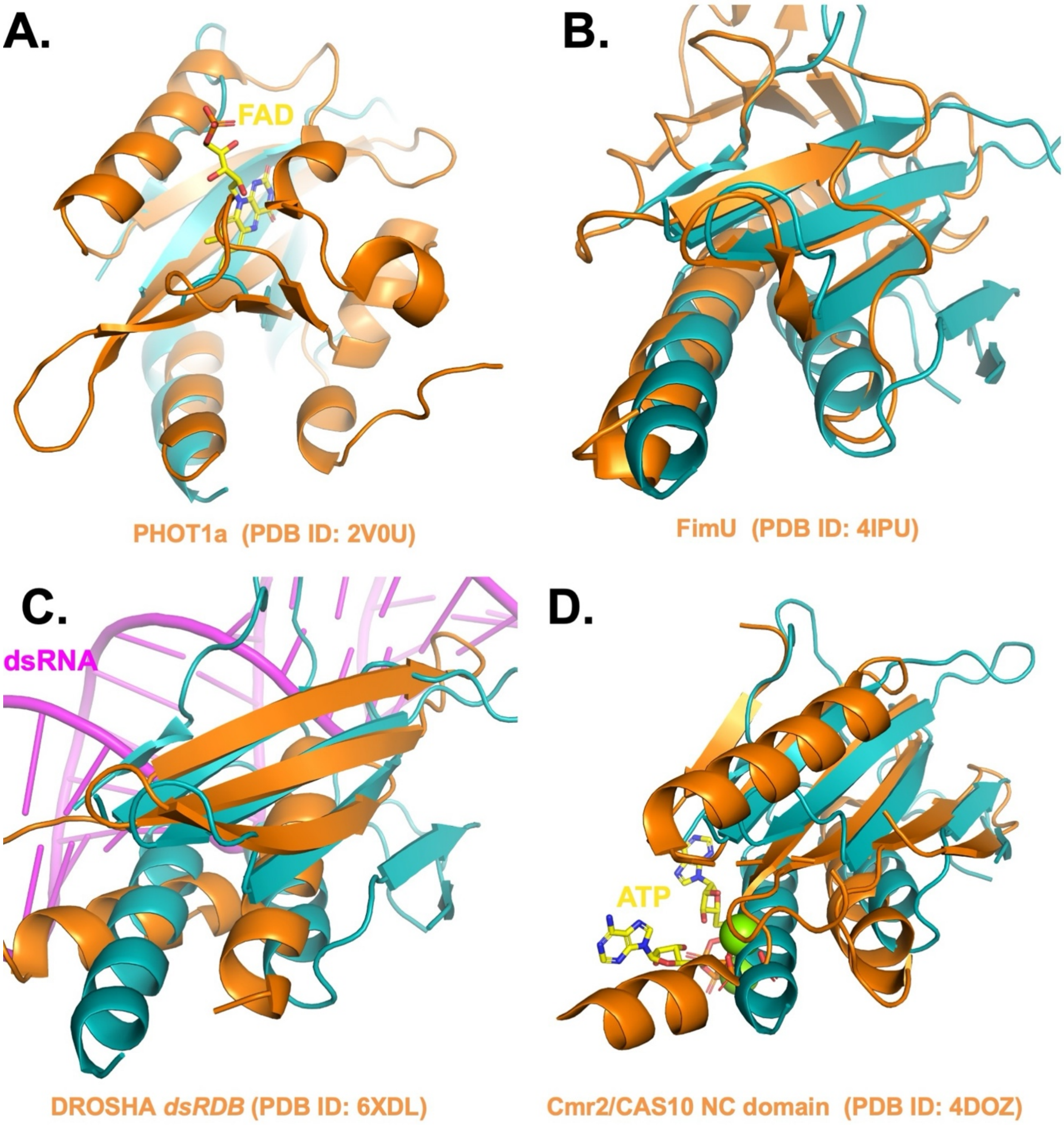

Appendix Figure S8. DUF domain core structural homologues identified by DALI. The figure shows alignments between the coordinates of the DUF domain core (aa 557-570+620-655+684-745; teal, this work) and the most representative hits from a full-PDB DALI search (orange). **A.** Structural alignment with nonphototropic hypocotyl / phototropin 1a (NPH1-1/PHOT1a) from *Avena sativa* (PDB ID: 2V0U (Halavaty & Moffat, 2007)). The FAD cofactor, sandwiched between the central β-sheet and an α-helix, is represented as sticks and colored in yellow. **B.** Structural alignment with the FimU type IV minor pilin from *Pseudomonas aeruginosa* (PDB ID: 4IPU (Nguyen et al, 2015)). **C.** Structural alignment with double-strand-RNA-binding domain (dsRDB) of the ribonuclease DROSHA (PDB ID: 6XDL (Kwon et al, 2016)). A double strand of RNA, captured interacting with the dsRDBm, is represented as cartoon and colored in magenta. The superimposition places the RNA molecule at a site that corresponds to the BTF-1/BTF-2/DUF interdomain cleft in CCTX2. **D.** Structural alignment with nucleotide cyclase (NC) of the Cmr2/CAS10 nucleotide cyclase-related enzyme in type III CRISPR-Cas (PDB ID: 4DOZ (Zhu & Ye, 2012)). Two ATP molecules, bound at the catalytic site of

Cmr2, are represented as sticks and colored in yellow. As in case of the alignment in panel **C**, the superimposition places the ligand at a site that corresponds to the BTF-1/BTF-2/DUF domain cleft of CCTX2.

A.

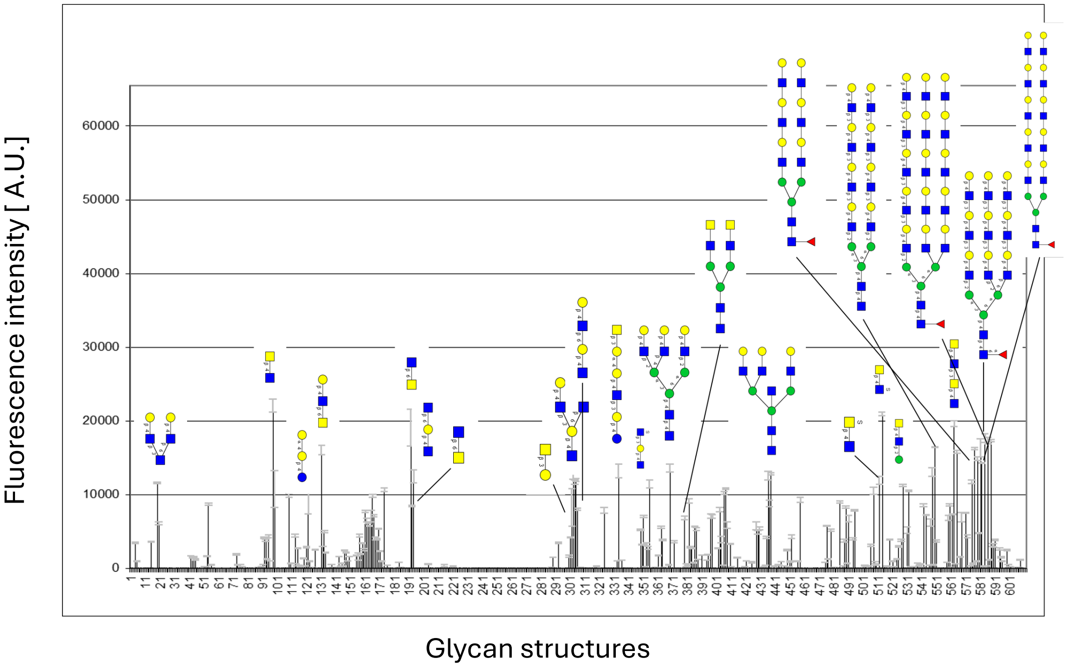

B.

Unbound structures:

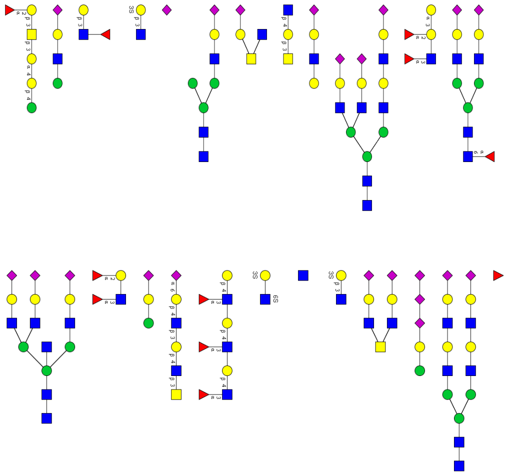

Legend:

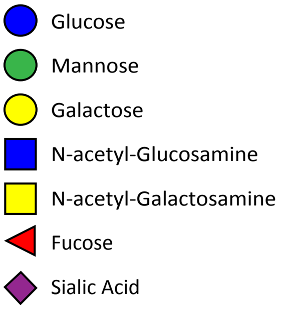

Appendix Figure S9. Glycan array analysis of CCTX2 and the N-terminally truncated CCTX2ΔN. Purified CCTX2 and CCTX2ΔN protein was fluorescently labeled with Alexa Fluor 488 (Invitrogen). The labeled protein was used, at various concentrations, to screen the mammalian glycan array (version 5.1) of Core H of the Consortium for Functional Glycomics (CFG). The data of this analysis are provided as Supplementary Datasets EV1-5. **A.** Histogram displaying the binding of CCTX2 at a concentration of 20 µg/ml to the glycan array. Glycans with the highest affinity, containing terminal LacNAc and LacDiNAc, are represented as symbols according to (Varki et al, 2015). **B.** Exemplary glycan structures from the glycan array screen that contain LacNAc and LacDiNAc motifs but are not bound by CCTX2. In these cases, the motifs are decorated with additional monosaccharides.

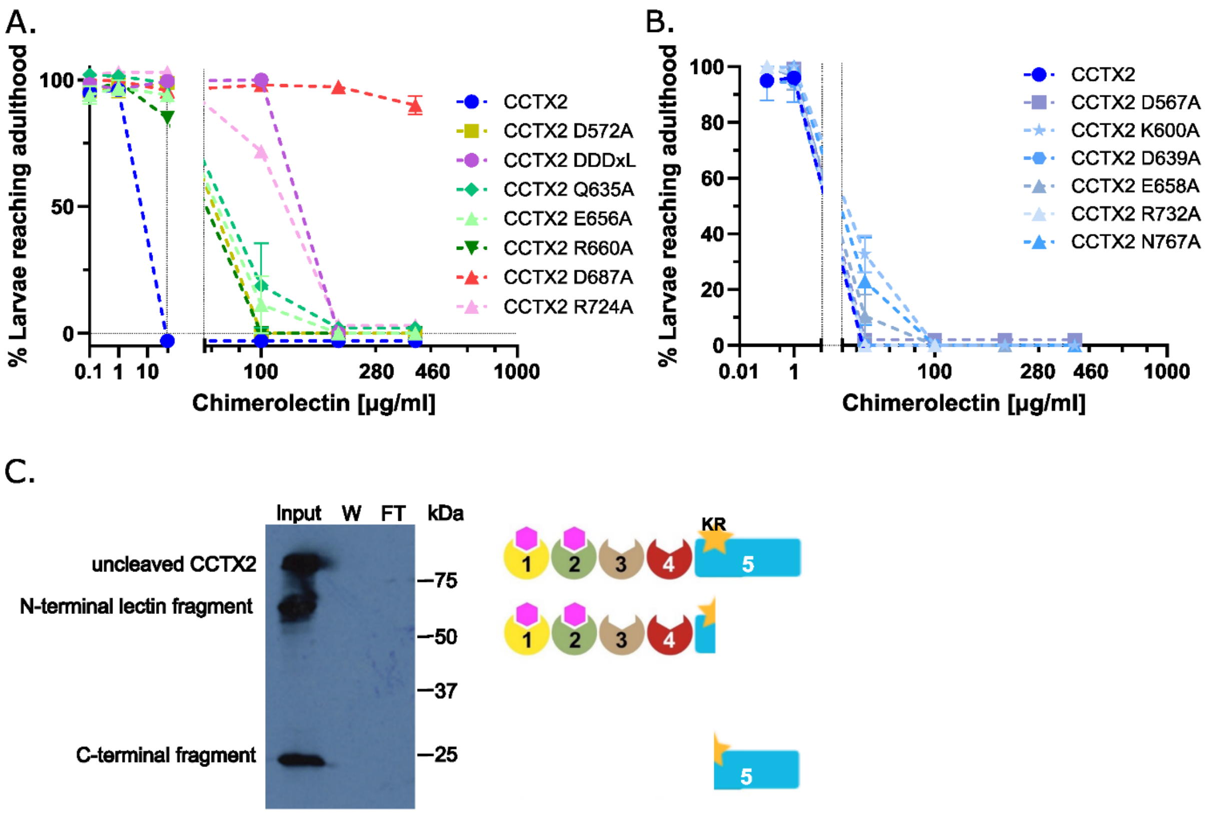

Appendix Figure S10. Alanine scanning of conserved residues within the C-terminal DUF domain. A. Nematoxicity assay with CCTX2 variants carrying one of seven single residue substitutions that conferred a moderate resistance to the toxin (>100 to <400 µg/ml). WT CCTX2 (dark blue) and D687A, which is part of the 'RxDxQ' motif (red) were included as references. **B.** Nematoxicity assay with CCTX2 variants carrying one of six single residue substitutions that did not reduce toxicity compared to wildtype CCTX2. Data points with error bars indicate means of N = 4 biological replicates with standard error of the mean (SEM). Overlapping data points were nudged by +/- 2 data units in *y* for better readability. **C**. *In vitro* cleavage assay of CCTX2 with recombinant *Saccharomyces cerevisiae* Kex2p protease. Kex2p-digested CCTX2 was subjected to a pulldown experiment using Sepharose beads. An immunoblot, using a custom-made anti-CCTX antiserum, of the various fractions on SDS-PAGE, showed that all the protein was found in the pulled-down fraction ('Input' lane), including the Kex2p-generated C-terminal fragment, which should be devoid of any lectin domains (BTF1-4). Conversely, no CCTX2 was detected in the wash ('W' lane) or flow-through ('FT' lane) fractions. Two CCTX2 proteolytic fragments (23 and 67 kDa), generated by the Kex2p digestion, are labeled on the left and shown as cartoons on the right side of the gel picture.

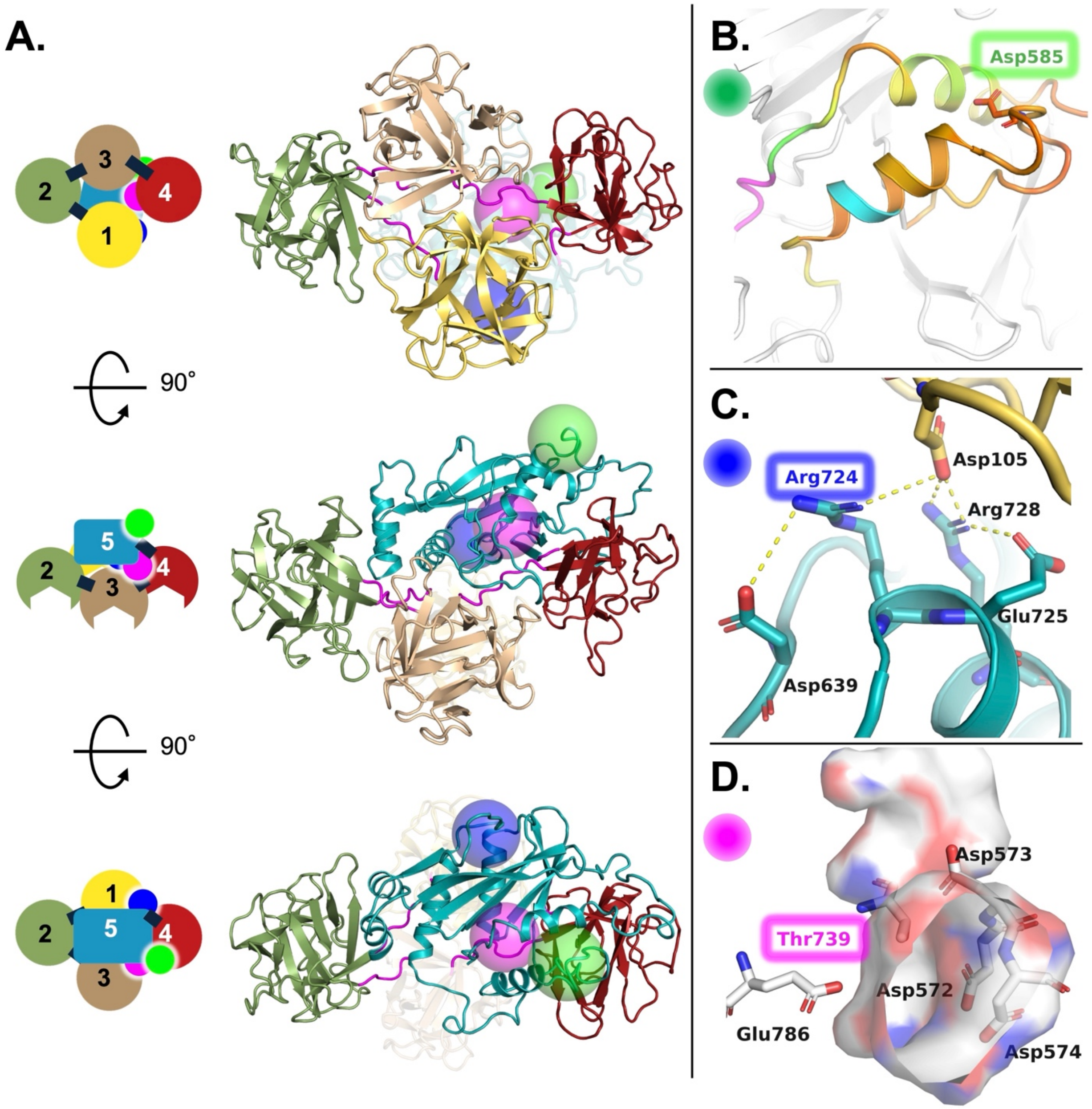

Appendix Figure S11. Structural details of functionally relevant Ala mutations. A. Cartoon representation of the CCTX2 structure in three different orientations, schematically presented on the left; a green sphere marks the position of Asp585, a blue sphere that of Arg724 and a magenta sphere that of Thr739. **B.** Position of Asp585 on the loop preceding helix-1; helix-1 and helix-6 are colored according to their Cα *B*-factors, with the kexin cleavage site marked in cyan and the HSEL sequon in magenta. **C.** Interaction between Arg724 and surrounding residues, suggesting its possible role in stabilizing the CCTX2 fold by mediating the interaction between the DUF domain (teal) and BTF domain 1 (yellow). **C.** Thr739 lying at the bottom of a pocket, lined by the negatively-charged Glu786 and aspartate residues from the DDDxL motif (Asp572, Asp573 and Asp574).

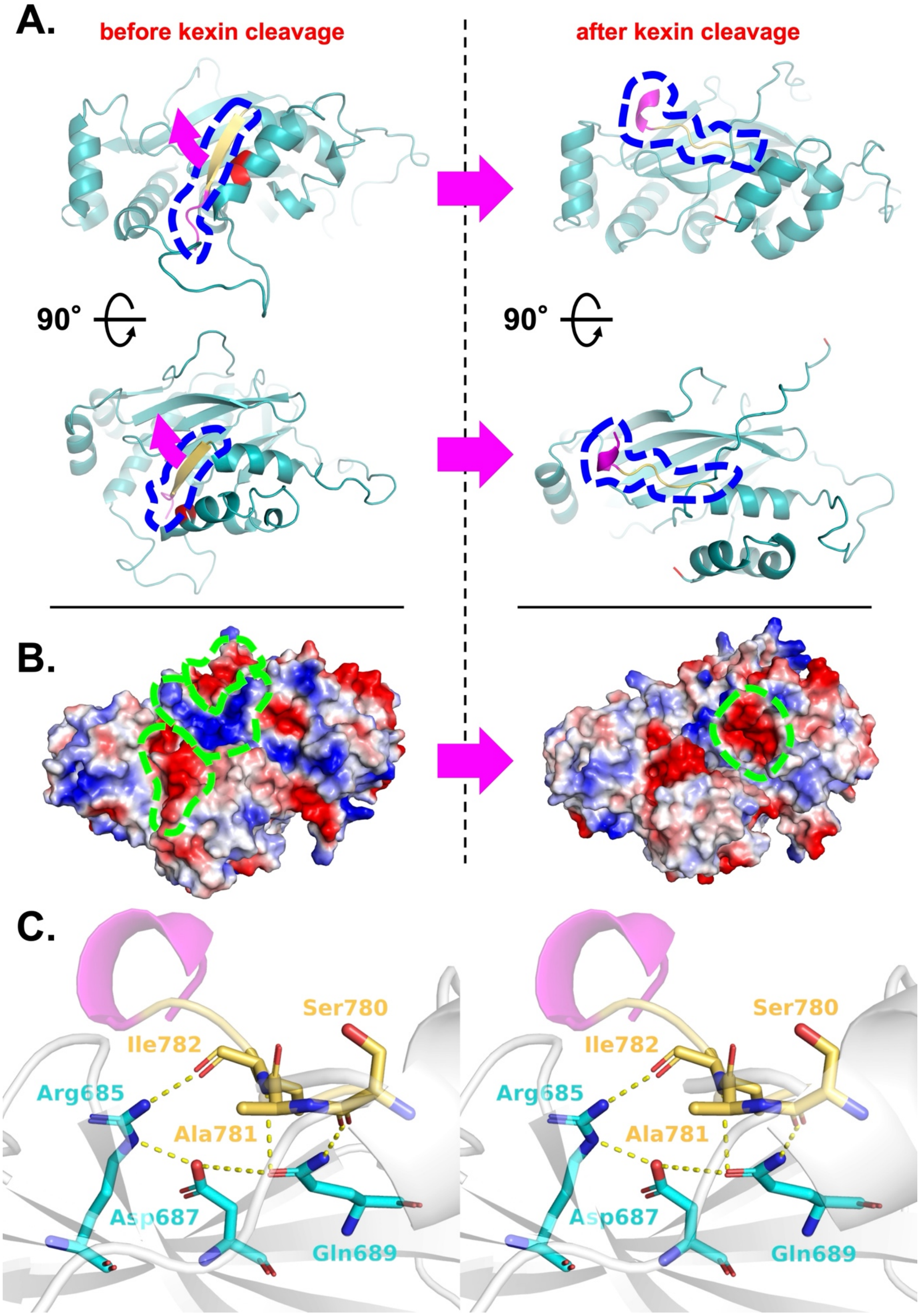
A.
before kexin cleavage
after kexin cleavage
90°
90°
B.
C.
Ser780
Ile782
Arg685
Ala781
Asp687
Gln689
Ser780
Ile782
Arg685
Ala781
Asp687
Gln689

Appendix Figure S12. AlphaFold3 model of post-Kex2-cleavage CCTX2. A. Cartoon representation of CCTX2 DUF domain (teal), with the last nine amino acids colored in yellow (aa768-783) and magenta (aa 784-787, HSEL sequon); the KR sequon recognized by Kex2/furin is colored in red; the blue arrow shows the direction of the conformational change of the C-terminus (outlined in blue). The left panels show the structure from cryo-EM data (this study); the right panels show the result from an AlphaFold3 simulation of the Kex2-cleavage products of CCTX2. The upper row shows the domain in 'side' view, while the lower panels show it in 'top' view, after a 90° rotation. **B.** Surface charge potential of CCTX2 before (left, cryo-EM data) and after (right, AlphaFold3 simulation) Kex2 cleavage. The green dashed lines mark areas of homogenous charge: after the cleavage, the conformational change exposed a patch of negative charge, corresponding to the DDDxL motif. **C.** Stereo view of the interaction between the RxDxQ motif (cyan) and the C-terminus of CCTX2 (yellow/magenta), after its AlphaFold-predicted conformational rearrangement towards the solvent-exposed surface of the DUF domain β-sheet. Interacting side chains are represented as sticks, while the rest of the domain is depicted in cartoon representation.

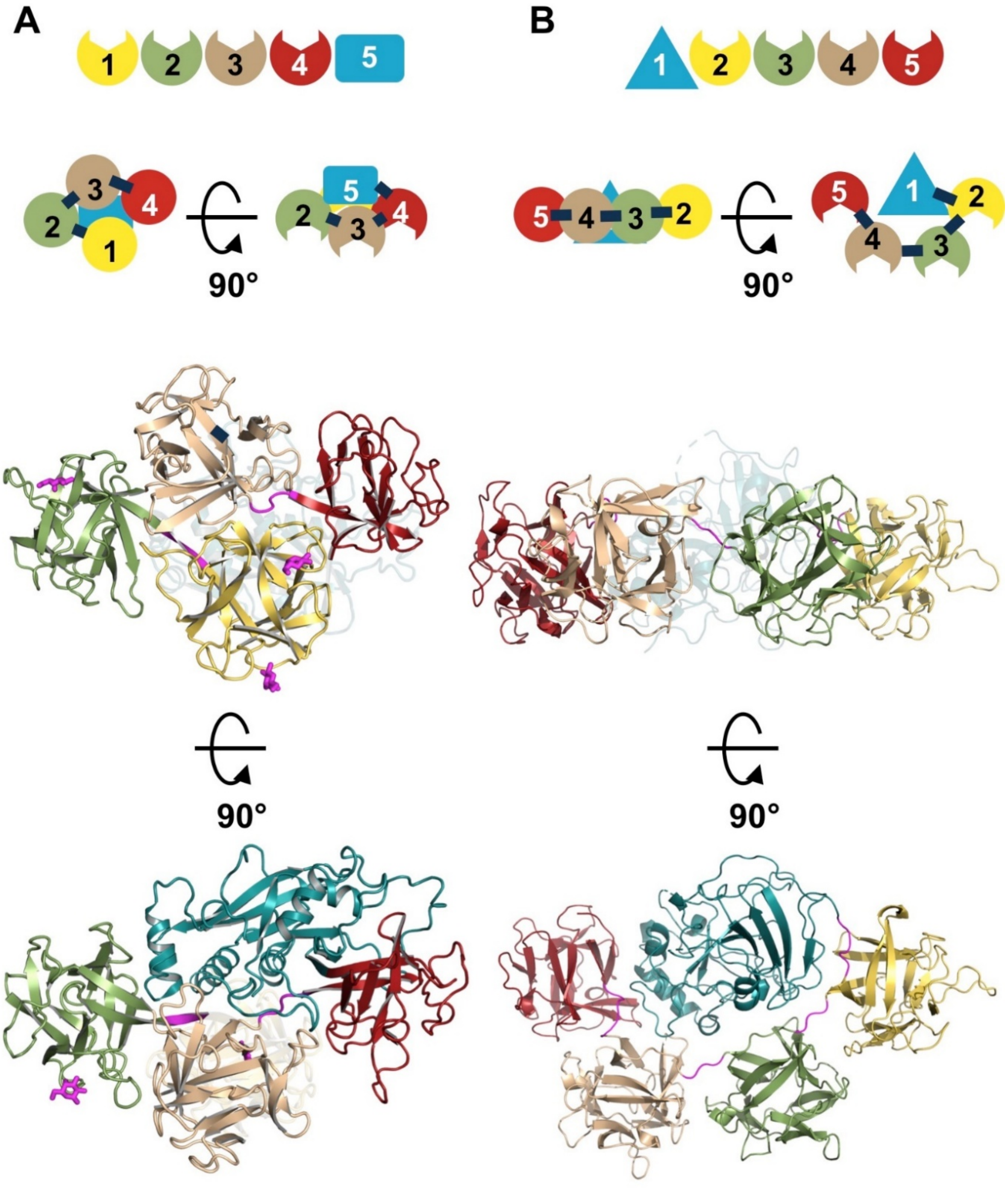

Appendix Figure S13. Comparison of CCTX2 with the mosquitocidal holotoxin from *Pieris rapae* (MTX). **A.** Schematic presentation of CCTX2 domains and topology of the protein tertiary structure (PDB ID: 9S11, this work). In magenta: interdomain linkers and D-galactose, likely retained from the purification process, modeled into weak density. **B.** Schematic presentation of MTX domains and topology of the protein tertiary structure (PDB ID: 2VSA (Treiber et al, 2008)). In magenta: interdomain linkers.

Appendix Table S1. List of full-length CCTX2 homologs in the fungal kingdom

Name and number of homologues on the tree	JGI Genome Assembly ID	JGI Protein ID
Coprinopsis cinerea AB CCTX1	Coprinopsis cinerea AmutBmut v2.0	2257338
Coprinopsis cinerea AB CCTX2	Coprinopsis cinerea AmutBmut v2.0	2257340
Coprinopsis cinerea AB CCTX3	Coprinopsis cinerea AmutBmut v2.0	2257352
Nemania sp.	Nemania sp. FL0916 v1.0	558949
Valsariales sp.	Valsariales sp. FL0756 v1.0	248463
Trichoderma harzianum	Trichoderma harzianum T22 v1.0	634717
Pterula gracilis	Pterula gracilis CBS309.79 v1.0	384083
Stereum hirsutum	Stereum hirsutum FP-91666 SS1 v1.0	116459
Choiromyces venosus 1	Choiromyces venosus 120613-1 v1.0	1801069
Choiromyces venosus 2	Choiromyces venosus 120613-1 v1.0	1809985
Earliella scabrosa	Earliella scabrosa CIRM-BRFM 1817 v1.0	854234
Ceriporiopsis subvermispora B 1	Ceriporiopsis (Gelatoporia) subvermispora B	125532
Ceriporiopsis subvermispora B 2	Ceriporiopsis (Gelatoporia) subvermispora B	97606
Ceriporiopsis subvermispora B 3	Ceriporiopsis (Gelatoporia) subvermispora B	117225
Ceriporiopsis subvermispora B 4	Ceriporiopsis (Gelatoporia) subvermispora B	117217
Sistotrema sernanderi	Sistotrema sernanderi OMC 1753 v1.0	84892
Mycena pura	Mycena pura 9144 v1.0	592943
Phlebia subcretacea 1	Phlebia subcretacea OMC74 v1.0	1018667
Phlebia subcretacea 2	Phlebia subcretacea OMC74 v1.0	2053875
Pisolithus orientalis	Pisolithus orientalis OTSU v2.0	900110
Pisolithus sp. 1	Pisolithus sp. B1 v1.0	617230
Pisolithus sp. 2	Pisolithus sp. B1 v1.0	1098360
Pisolithus albus 1	Pisolithus albus SI12 v1.0	2367539
Pisolithus albus 2	Pisolithus albus SI12 v1.0	2358149
Pisolithus microcarpus	Pisolithus microcarpus 441 v2.0	17071
Pisolithus croceorrhizus ssp. 2 1	Pisolithus croceorrhizus ssp. 2 72 v1.0	2234632
Pisolithus croceorrhizus ssp. A 1	Pisolithus croceorrhizus subspA 74A v1.0	1628709
Pisolithus croceorrhizus ssp. A 2	Pisolithus croceorrhizus subspA 74A v1.1	1583998
Pisolithus croceorrhizus ssp. 2 2	Pisolithus croceorrhizus ssp. 2 72 v1.0	1889515
Pisolithus thermaeus	Pisolithus thermaeus 11 v1.0	1506882
Porodaedalea niemelaei	Porodaedalea niemelaei PN71-100-IP13 v1.0	795801

Appendix Table S2. Cryo-EM data collection and model refinement statistics

**Data collection and processing**	
**EMDB ID**	EMD-54430
**Microscope**	FEI Titan Krios
**Detector**	Gatan K2
**Voltage (kV)**	300
**Magnification (µm)**	215,000
**Pixel size (Å)**	5
**Total dose (e^-/Å^2)**	59.5
**Defocus range (µm)**	-1.5 to -3.0
**Micrographs collected**	7,500
**Micrographs used**	7,498
**Particles (initial extraction / final reconstruction)**	1,794,120 / 204,500
**Symmetry imposed**	n/a (C1)
**Map sharpening**	Global *B*-factor sharpening
**Map resolution (Å)**	3.16
**FSC threshold**	0.143
**Refinement**	
**PDB ID**	9S11
**Initial model used**	Robetta-generated (Kim et al., 2004), template-based and *ab initio* AlphaFold 3 (Abramson et al., 2024)
**Box**	
Pixels	256, 256, 256
Angles (°)	90.00, 90.00, 90.00
**Pixel size (Å) (map)**	1.18
**Map-to-model CC**	
Mask	0.78
Box	0.88
Peaks	0.79
Volume	0.79
**Composition**	
Atoms	6276
Amino acids	787
Other ligands	0
***B*-factors (Å^2)**	
Protein (min/max/mean)	26.4/122.1/63.4
**r.m.s.d. from ideal values**	
Bond length (Å)	0.01
Bond angle (°)	0.84
**Validation**	
MolProbity score (Williams et al, 2018)	2.89
Clashscore	21.55
**Ramachandran plot**	
Favored (%)	85.0
Allowed (%)	14.0
Outliers (%)	1.0[a]
**Rotamer outliers (%)**	3.4
**CaBLAM outliers (%)**	8.9

[a]Ramachandran outliers: eight outliers, as estimated by Phenix (Liebschner et al, 2019) cryo-EM validation tool and RAMPAGE (Lovell et al, 2003). The use of a different tool, employing different thresholds, could yield a different estimate.

Appendix Table S3. Poorly-defined regions in the cryo-EM map

Residue range	Domain	Description
15-19	BTF-1	Subdomain α, loop forming the hypothetical sugar-binding site α_1
24-29	BTF-1	Subdomain α, loop supporting the hypothetical sugar-binding site γ_1
355-360	BTF-3	Subdomain β, surface loop at the interface with domain BTF- 1
458-466	BTF-4	Subdomain β, surface loop
522-529	BTF-4	Subdomain γ, surface loop
531-548	BTF-4	Subdomain γ, loop forming the hypothetical sugar-binding site γ_4
578-589	DUF	Loop connecting DUF strand-2 with DUF helix-1
603-612	DUF	Loop connecting DUF helix-1 with DUF helix-2, supported by domain BTF-1
676-683	DUF	Loop connecting DUF helix-4 (part of DUF insert-2) with DUF strand-4
756-770	DUF	Loop connecting DUF strand-7 with helix-6

Appendix Table S4. CCTX paralogue domain boundaries and features outline

Orthologue				CCTX1	CCTX2	CCTX3
**GenBank ID**				CC1G_10075	CC1G_10077	CC1G_10083
**UniProt ID**				A8NDT5	A8NDT7	A8NDU3
**Model source**				Robetta homology model	cryo-EM	Robetta homology model
**Protein length (AA)**				774	787	783
**BTF domain 1**	**Fold**			ricin B chain-like	ricin B chain-like	ricin B chain-like
	**Boundaries**	**Domain**		1-150	1-150	1-150
		**Subdomain α**		1-56	1-56	1-56
		**Subdomain β**		57-107	**57-107 ***	57-107
		**Subdomain γ**		108-150	**108-150 ***	108-150
	**(QxW)₃**	**Subdomain α**	**Q**	Q 48	Q 48	Q 48
			**W**	W 50	W 50	W 50
		**Subdomain β**	**Q**	Q 96	Q 96	Q 96
			**W**	W 98	W 98	W 98
		**Subdomain γ**	**Q**	Q 45	Q 145	Q 145
			**W**	W 147	W 147	W 147
**Linker 1-2**	**Boundaries**			151-156	151-156	151-156
**BTF domain 2**	**Fold**			ricin B chain-like	ricin B chain-like	ricin B chain-like
	**Boundaries**	**Domain**		157-294	157-296	157-294
		**Subdomain α**		157-204	**157-204 ***	157-204
		**Subdomain β**		205-252	205-254	205-252
		**Subdomain γ**		253-294	255-296	253-294
	**(QxW)₃**	**Subdomain α**	**Q**	Q 196	Q 196	Q 196
			**W**	W 198	W 198	W 198
		**Subdomain β**	**Q**	Q 244	Q 244	Q 244
			**W**	W 246	W 246	W 246
		**Subdomain γ**	**Q**	Q 289	Q 291	Q 289
			**W**	W 291	W 293	W 291
**Linker 2-3**	**Boundaries**			295-298	297-300	295-298
**BTF domain 3**	**Fold**			ricin B chain-like	ricin B chain-like	ricin B chain-like
	**Boundaries**	**Domain**		299-435	301-436	299-436
		**Subdomain α**		299-345	301-347	299-346
		**Subdomain β**		346-393	348-396	347-394
		**Subdomain γ**		394-435	397-436	395-436
	**(QxW)₃**	**Subdomain α**	**Q**	V 337	V 339	V 337
			**W**	W 339	W 341	W 339
		**Subdomain β**	**Q**	D 383	D 385	D 383
			**W**	W 385	W 387	R 385
		**Subdomain γ**	**Q**	R 430	R 431	R 430
			**W**	W 432	W 433	W 432
**Linker 3-4**	**Boundaries**			436-442	437-443	437-442
**BTF 4 domain**	**Fold**			ricin B chain-like	ricin B chain-like	ricin B chain-like
	**Boundaries**	**Domain**		443-552	444-554	443-552
		**Subdomain α**		443-484	443-487	443-485
		**Subdomain β**		485-513	488-516	486-514
		**Subdomain γ**		514-552	517-555	515-552
	**(QxW)₃**	**Subdomain α**	**Q**	A 475	A 478	A 476
			**W**	W 477	W 480	W 478
		**Subdomain β**	**Q**	F 506	A 507	A 505
			**W**	L 508	W 509	W 507
		**Subdomain γ**	**Q**	Q 547	Q 549	Q 547
			**W**	W 549	W 551	F 549
**Linker 4-5**	**Boundaries**			553-554	555-556	553-554
**DUF domain 5**	**Fold**			Unknown (α+β)	Unknown (α+β)	Unknown (α+β)
	**Boundaries**			555-774	557-787	555-783
	**Kexin cleavage site**			586-587	596-597	591-592

*Marked in **red and bold**: active sugar-binding sites (putative).

Appendix Table S5. BTF domain homology

PDB ID	chain	Z-score	r.m.s.d.	aligned residues	total protein length	% identity	Protein name	Organism	Function	UniProt	Reference
**BTF domain 1**											
3PG0	A	23.8	1.3	135	140	26	synthetic β-threefoil protein	synthetic	proof-of-concept	synthetic	(Broom et al, 2012)
8BAD	B	23.1	1.5	140	351	15	Tpp80Aa1 toxin	*Bacillus thuringiensis*	chimerolectin/ toxin	A0A4V1G8Q1	(Best et al, 2022)
3EF2	A	22.8	1.9	143	292	27	*Marasiums oreades* agglutinin (MOA)	*Marasmius oreades*	chimerolectin/proteolytic toxin	Q8X123	(Grahn et al, 2007)
3VSF	C	22.8	1.3	138	482	25	1,3Gal43A	*Acetivibrio thermocellus ATCC 27405*	exo-β-1,3-galactanase	A3DD67	(Jiang et al, 2012)
2X2T	A	21.7	1.8	140	152	17	*Sclerotinia sclerotiorum* agglutinin (SSA)	*Sclerotinia sclerotiorum*	lectin	A7XUK7	(Sulzenbacher et al, 2010)
3NBE	B	21.4	1.9	138	147	25	*Clitocybe nebularis* ricin B-like lectin (CNL)	*Clitocybe nebularis*	lectin	B2ZRS9	(Pohleven et al, 2012)
3PHZ	B	21.3	1.7	135	278	27	*Polyporus squamosus* lectin 1a (PSL1a)	*Polyporus squamosus*	chimerolectin/proteolytic toxin	Q75WT9	(Kadirvelraj et al, 2011)
4G9M	A	20.9	1.6	132	142	20	*Rhizoctonia solani* agglutinin	*Rhizoctonia solani*	lectin	L8WGI4	(Skamnaki et al, 2013)
2VSA	A	20.3	1.7	135	822	21	Mosquitocidal holotoxin (MTX)	*Bacillus sphaericus*	chimerolectin/toxin	Q03988	(Treiber et al., 2008)
1V6W	A	20.1	1.7	128	436	24	xylanase	*Streptomyces olivaceoviridis*	endo-1,4-β-D-xylanase	Q7SI98	(Fujimoto et al, 2004)
**BTF domain 2**											
3PG0	A	23.0	1.8	134	140	26	synthetic β-threefoil protein	synthetic	proof-of-concept	synthetic	(Broom et al., 2012)
3VSF	E	22.6	1.7	134	461	28	1,3Gal43A	*Acetivibrio thermocellus ATCC 27405*	exo-β-1,3-galactanase	A3DD67	(Jiang et al., 2012)
2IHO	A	22.0	2.2	139	292	27	*Marasiums oreades* agglutinin (MOA)	*Marasmius oreades*	chimerolectin/proteolytic toxin	Q8X123	(Grahn et al., 2007)
8BAD	B	21.9	1.7	125	351	19	Tpp80Aa1 toxin	*Bacillus thuringiensis*	chimerolectin/ toxin	A0A4V1G8Q1	(Best et al., 2022)
3A21	A	20.7	1.6	125	614	29	β-L-arabinopyranosidase	Streptomyces avermitilis	β-L-arabinopyranosidase	Q82L26	(Ichinose et al, 2009)
4G9M	A	20.3	1.8	128	142	27	*Rhizoctonia solani* agglutinin	*Rhizoctonia solani*	lectin	L8WGI4	(Skamnaki et al., 2013)
6IFA	C	20.2	1.6	124	126	22	β-trefoil lectin (EntTref)	*Entamoeba histolytica* HM-1:IMSS	lectin	N9TFI9	(Khan et al, 2020)
5GQD	A	20.2	1.6	122	427	27	Glycoside Hydrolase Family 10 Xylanase	*Streptomyces olivaceoviridis*	xylanase	Q7SI98	(Suzuki et al, 2018)
3NBE	B	19.7	2.5	133	147	18	*Clitocybe nebularis* ricin B-like lectin (CNL)	*Clitocybe nebularis*	lectin	B2ZRS9	(Pohleven et al., 2012)
2VLC	B	19.6	1.7	123	518	18	Cinnamomum camphora	*Cinnamomum camphora*	ribosome inactivating protein	Q94BW3	(Azzi et al, 2009)
**BTF domain 3**											
3AJ6	A	19.5	1.9	130	283	19	botulinum type C progenitor toxin	*Clostridium botulinum*	toxin	P0DPR0	(Nakamura et al, 2011)
2X2S	B	19.1	2.0	133	152	12	*Sclerotinia sclerotiorum* agglutinin (SSA)	*Sclerotinia sclerotiorum*	lectin	A7XUK7	(Sulzenbacher et al., 2010)
3EF2	A	19.1	2.1	134	292	11	*Marasiums oreades* agglutinin (MOA)	*Marasmius oreades*	chimerolectin/proteolytic toxin	Q8X123	(Grahn et al, 2009)
1QXM	A	19.1	1.9	130	283	19	hemagglutinin component (HA1)	*Clostridium botulinum* D phage	lectin	P0DPR1	(Inoue et al, 2003)
3O49	A	18.8	1.7	122	123	17	Symfoil-1	synthetic	proof-of-concept	synthetic	(Lee & Blaber, 2011)
3VSF	E	18.6	1.9	126	461	17	1,3Gal43A	Acetivibrio thermocellus ATCC 27405	exo-β1,3-galactanase	A3DD67	(Jiang et al., 2012)
1UPS	B	18.2	2.1	125	397	21	GlcNAcα1-4Gal	Clostridium perfringens	endo-β-galactosidase	Q934G8	(Tempel et al, 2005)
3PHZ	B	18.1	1.9	127	278	17	*Polyporus squamosus* lectin 1a (PSL1a)	*Polyporus squamosus*	chimerolectin/proteolytic toxin	Q75WT9	(Nakamura et al, 2008)
7KCG	A	18.1	1.7	124	143	19	mosquito salivary protein	*Culex quinquefasciatus*	lectin (probable)	Q6TRZ5	(Kern et al, 2021)
2VSA	A	18.0	2.0	127	822	13	Mosquitocidal holotoxin (MTX)	*Bacillus sphaericus*	chimerolectin/toxin	Q03988	(Treiber et al., 2008)
**BTF domain 4**											
3O4D	A	16.2	1.6	104	123	16	Symfoil-4P	synthetic	proof-of-concept	synthetic	(Lee & Blaber, 2011)
4G9N	A	15.4	1.8	109	142	21	Rhizoctonia solani agglutinin	*Rhizoctonia solani*	lectin	L8WGI4	(Skamnaki et al., 2013)
3VSF	A	15.4	1.7	108	461	17	1,3Gal43A	*Acetivibrio thermocellus ATCC 27405*	exo-β-1,3-galactanase	A3DD67	(Jiang et al., 2012)
1BFC	A	15.0	2.0	106	124	17	bFGF - basic fibroblast growth factor/FGF2	*Homo sapiens*	growth factor	P09038	(Faham et al, 1996)
7ZNX	A	14.9	2.1	107	136	20	cocaprin 1	*Coprinopsis cinerea*	cysteine and aspartic proteases inhibitor	A8PCJ3	(Renko et al, 2022)
3UD7	C	14.9	2.1	107	127	13	aFGF - basic fibroblast growth factor/FGF1	*Homo sapiens*	hormone	P05230	(Hu et al, 2012)
3AJ6	A	14.7	1.7	106	283	14	HA33/C (HA1) module of type C neurotoxin	*Clostridium botulinum*	toxin	P0DPR0	(Nakamura et al., 2011)
4IYB	B	14.5	1.9	107	140	15	Macrolepiota procera ricin B-like lectin (MPL)	*Macrolepiota procera*	lectin	F6KMV5	(Žurga et al, 2014)
8BAD	A	14.5	1.8	109	350	11	Tpp80Aa1	*Bacillus thuringiensis*	toxin	A0A4V1G8Q1	(Best et al., 2022)
2VSE	B	14.5	1.7	106	822	16	Mosquitocidal holotoxin (MTX)	*Bacillus sphaericus*	chimerolectin/toxin	Q03988	(Treiber et al., 2008)

Appendix Table S6. CCTX2 β-trefoil fold (BTF) domains: Sequence identity (%) and structural similarity (r.m.s.d. (Å), Cα alignment)

vs.	**BTF domain 1**	**BTF domain 2**	**BTF domain 3**	**BTF domain 4**
**BTF domain 1**	-	35.8% / 1.9 Å	9.8% / 2.5 Å	14.2% /2.9 Å
**BTF domain 2**	35.8% / 1.9 Å	-	11.7% /2.7 Å	17.1% /2.8 Å
**BTF domain 3**	9.8% / 2.5 Å	11.7% /2.7 Å	-	18.4% /3.4 Å
**BTF domain 4**	14.2% /2.9 Å	17.1% /2.8 Å	18.4% /3.4 Å	-

Appendix Table S7. DUF domain (full-length) homology (DALI)

PDB ID	chain	Z-score	r.m.s.d.	aligned residues	total protein length	% identity	Protein name	Organism	Function	UniProt	Reference
8AHX	E	2.7	3.7	95	213	2	RfnE (ion-translocating oxidoreductase complex RFN1, subunit E)	*Azotobacter vinelandii DJ*	Integral membrane protein, forming a dimer with the related protein RfnA. Part of RFN1, a membrane-bound complex, involved in nitrogen fixation, that couples electron transfer with translocation of ions across the membrane.	Q9F5Y1	(Zhang & Einsle, 2024)
7O5Y	D	2.7	5.1	89	131	4	PilA	*Streptococcus sanguinis*	Type IV pilus minor pilin. Together with PilB and PilC forms a tip-located complex promoting adhesion to various host receptors	A0A0B7GNW3	(Shahin et al, 2023)
2RET	A	2.7	3.2	75	84	3	EpsI (extracellular protein secretion system subunit I)	*Vibrio vulnificus*	Pseudopilin. Component of the Type II secretion system, required for the energy-dependent secretion of extracellular factors such as proteases and toxins from the periplasm.	Q7MPZ1	(Yanez et al, 2008)
**8EDG**	A	2.6	4.4	96	550	15	*Hermes* transposase	*Musca domestica*	*Hermes* transposases, encoded by the eukaryotic class II *Hermes* transposon; forms a ring-shaped tetramer of dimers. Homology to the *Hermes*' RNase H-like domain (aa 143-264+552-610).	Q25438	(Lannes et al., 2023)
**6GRF**	B	2.4	3.0	76	211	7	PDLP8 (plasmodesmata-located protein 8)	*Arabidopsis thaliana*	DUF26 ectodomain of cysteine-rich receptor-like protein PDLP8. Full protein modulates cell-to-cell trafficking. DUF26 is land plant-specific but structural analyses of PDLP ectodomains revealed strong similarity to fungal lectins and thus may constitute a group of plant carbohydrate-binding proteins. Similar to DUF26 of cysteine-rich receptor-like secreted protein (CRRSP) Gnk2, a mannose-binding lectin in vitro with antifungal activity	Q6NKQ9	(Vaattovaara et al., 2019)
**9JDI**	C	2.2	3.8	77	164	12	BtpiA (Bacteroides T6SS phosphatase effector A)	*Bacteroides fragilis*	Cognate immunity protein/inhibitory protein forming a heterodimer with the amidase BtpeA, a Type 6 Secretion System (T6SS) effector. Together with another effector, BtpeB, it exerts distinct cell-wall destructive activities, critical for interspecies competition.	A0A5C6HE89	(Li et al., 2025)
7LDK	B	2.2	4.9	84	106	8	NS2 (nonstructural protein 2)	*Human respiratory syncytial virus A strain Long*	Plays several role in blocking the host immune response.	Q86305	(Pei et al, 2021)
8CIL	A	2.2	5.4	108	365	8	Fic (filamentation induced by cyclic AMP) protein 2)	*Coxiella burnetii*	posttranslational AMPylation by transferring adenosine monophosphate (AMP) from adenosine triphosphate (ATP) to a hydroxyl-containing side chains 10 and 28 of histone H3	Q83DB6	(Höpfner et al, 2023)
**3A2E**	A	2.2	3.2	69	108	12	ginkbilobin-2	*Ginkgo biloba*	antifungal protein, homologous to the DUF26 extracellular domain of plant cysteine-rich receptor-like kinases.	A4ZDL6	(Miyakawa et al., 2009)
8WJ5	A	2.1	3.2	69	130	12	YdcD	*Escherichia coli K-12*	Double-wing domain of *E. coli* uncharacterized protein YdcD, possibly an antitoxin to YdcE (endoribonuclease of the MazF/PemK family, inactivates cellular mRNAs) (Pellegrini et al, 2005)	P31991	*unpublished*
7C8Z	B	2.1	4.2	81	161	7	NagH	*Ralstonia sp.*	Small oxygenase component of salicylate 5-hydroxylase (S5H), forms a heterodimer with NagG S5H is a multicomponent enzyme system which catalyzes the 5-hydroxylation of salicylate to gentisate. Active only on substrates with a ring-substituted carboxylate group with an adjacent hydroxyl group. NagH regulates the catalytic activity of NagG. NagH carries a cystatin fold.	O52380	(Hou et al, 2021)
6BAQ	F	2.0	3.4	77	211	5	BPIFA1 (Bacterial permeability-increasing family member A1)	*Mus musculus*	Lipid-binding protein which shows high specificity for the surfactant phospholipid dipalmitoylphosphatidylcholine (DPPC). Innate immunity factor with antimicrobial, surfactant and lipopolysaccharide-binding activities, as well as established roles in lung hydration	P97361	(Little & Redinbo, 2018)

Entries visually matching the CCTX2 DUF domain fold are marked in red and bold.

Appendix Table S8. DUF domain *core* homology (DALI)

PDB ID	chain	Z-score	r.m.s.d.	aligned residues	total protein length	% identity	Protein name	Organism	Function	UniProt	Reference
4IPU	A	3.6	3.6	71	137	7	FimU	*Pseudomonas aeruginosa*	type IV minor pilin	G3XCZ0	(Nguyen et al., 2015)
8ACX	B	3	3.8	71	113	14	KP4 (killer protein 4)	*Zymoseptoria tritici*	Antifungal toxin of viral origin. Ecp2 effector protein-like domain-containing protein. Ecp2 is a member of the Hce2 superfamily of effectors (Homologs of *C. fulvum* Ecp2), widely distributed within the fungal kingdom.	A0A2H1H404	(de Guillen et al, 2025)
1O70	A	3	4.1	76	296	5	Fasciclin i	*Drosophila melanogaster*	Neural cell adhesion molecule.	P10674	(Clout et al, 2003)
4V1A	H	2.5	3.7	51	289	6	ML37, MRPL37 (mitochondrial ribosomal protein L37)	*Sus scrofa*	Mitoribosomal protein. Binds the large subunit of the mammalian mitochondrial ribosome	no entry	(Greber et al, 2014)
4NDV	A	2.4	3.1	53	94	6	LDL (*Lyophyllum decastes* lectin)	*Lyophyllum decastes*	α-galactosyl-binding lectin	A7UNK4	(van Eerde et al, 2015)
5HO2	A	2.4	4.4	59	803	3	AbnA	*Geobacillus stearothermophilus*	Possible carbohydrate-binding domain of an extracellular GH43 (glycosyl hydrolases family 43) α-L-arabinanase	B3EYN2	(Lansky et al, 2022)
5TD8	D	2.4	2.3	53	128	9	Kinetochore protein SPC25	*Saccharomyces cerevisiae S288C*	component of the essential kinetochore-associated NDC80 complex	P40014	(Valverde et al, 2016)
4XEI	F	2.3	4.2	73	168	5	ARPC4 (actin-related protein 2/3 complex subunit 4)	*Bos taurus*	Arp2/3 complex mediates the formation of branched actin networks in the cytoplasm	Q148J6	(Jurgenson & Pollard, 2015)
4DOZ	A	2.3	3	64	557	8	Cmr2 (CAS10)	*Pyrococcus furiosus* DSM 3638	Nucleotide cyclase domain ot Cmr2, a nucleotide cyclase-related enzyme in type III CRISPR-Cas	Q8U1S6	(Zhu & Ye, 2012)
5V6I	A	2.3	3.9	67	112	9	Y3	*Coprinus comatus*	Glycan-binding protein, recognizing the LDNF glycoepitope (GalNAcβ1–4(Fucα1–3)GlcNAc) Cytotoxic towards T-cell leukemia Jurkat cells (mechanism unknown, relies on caspases 3, 8 and 9 activation)	G3BK00	(Zhang et al, 2017)
4V12	A	2.3	4.4	64	337	11	MSMEG_6754	*Mycolicibacterium smegmatis*	MaoC-like domain protein, dehydratase	A0R724	(Carrère-Kremer et al, 2015)
6KHI	V	2.3	6	64	109	5	NdhV (NAD(P)H dehydrogenase-like (NDH) complex, subunit V)	*Thermosynechococcus vestitus BP-1*	Soluble subunit oft he NAD(P)H dehydrogenase-like (NDH) complex NDH-1L of cyanobacteria. Act as a regulator oft he complex.	Q8DLL4	(Pan et al, 2020)
8X6F	C	2.3	3.1	50	1153	6	PpoB	*Staphylococcus aureus*	DNA-dependent RNA polymerase subunit alpha	P47768	(Yuan et al, 2024)
9UKM	I	2.3	3.8	62	303	8	DarA (Defense against restriction protein A)	*Homo sapiens*	Prevents degradation of viral DNA by the host type I restriction-modification antiviral defense system	Q06210	(Ruegenberg et al, 2020)
3WB2	A	2.2	4	76	160	5	HcgB	*Methanocaldococcus jannaschii DSM 2661*	Catalyzes the conjugation of the GMP moiety from GTP to a pyridinol precursor to form guanylylpyridinol	Q57912	(Fujishiro et al, 2013)
6XLD	A	2.2	4	54	918	4	DROSHA	*Homo sapiens*	Double-strand RNA binding domain (dsRBD) of DROSHA, a double-stranded (ds) RNA-specific endoribonuclease. Component of the microprocessor complex that is required to process primary miRNA transcripts (pri-miRNAs) to release precursor miRNA (pre-miRNA) in the nucleus.	Q9NRR4	(Kwon et al., 2016)
9VNJ	A	2.2	4.1	54	108	0	AtaA	*Acinetobacter sp. Tol 5*	Trimeric autotransporter adhesin	K7ZP88	(Yoshimoto et al, 2025)
5XVJ	A	2.1	2.6	58	134	10	ALFIN-LIKE 7	*Arabidopsis thaliana*	PHD finger protein, histone-binding component that specifically recognizes H3 tails trimethylated on 'Lys-4' (H3K4me3).	Q8LA16	(Peng et al, 2018)
6W17	F	2.1	3.8	60	168	5	Arc4 (Actin-related protein 2/3 complex subunit 4)	*Schizosaccharomyces pombe 972h-*	Actin-binding component of the Arp2/3 complex which is involved in regulation of actin polymerization and together with an activating nucleation-promoting factor (NPF) mediates the formation of branched actin networks.	Q92352	(Shaaban et al, 2020)
1KPT	A	2.1	3.7	59	105	10	KP4	*Ustilago maydis*	Toxin. It specifically inhibits voltage-gated calcium channels. It inhibits cell growth and division by blocking calcium import. The toxin is originally encoded by the *Ustilago maydis* P4 virus (UmV4).	Q90121	(Gu et al, 1995)
4AD9	A	2	4.5	65	288	5	LACTB2 (β-lactamase-like protein 2	*Homo sapiens*	Endoribonuclease; cleaves preferentially 3' to purine-pyrimidine dinucleotide motifs in single-stranded RNA.	Q53H82	(Levy et al, 2016)
2V0U	A	2	5.8	58	476	3	NPH1-1 (nonphototropic hypocotyl 1) PHOT1a (Phototropin 1a)	*Avena sativa*	Protein kinase that acts as a blue light photoreceptor in a signal-transduction pathway for phototropic responses	NPH1-1	(Halavaty & Moffat, 2007)

Appendix Table S9. Plasmids generated in this study, and toxicity results

Construct	Usage	Toxicity	Reference
pET24_CCTX2	Purification of CCTX2 (functional studies)	toxic	This study
pET24_CCTX1	Purification of CCTX1	toxic	This study
pET24_CCTX3	Purification of CCTX3	toxic	This study
pET24_CCTX2-ΔN	Purification of CCTX2ΔN deletion mutant (lacking residues 2-303)	non-toxic	This study
pET24_CCTX2-ΔC	Purification of CCTX2ΔC deletion mutant (lacking residues 570-787)	non-toxic	This study
pET24_CCTX2-ΔHSEL	Purification of CCTX2ΔHSEL deletion mutant (lacking residues 784-787)	non-toxic	This study
pET22_MOA	Purification of MOA	toxic	(Wohlschlager et al, 2011)
pET24_CCL2	Purification of CCL2	toxic	(Schubert et al, 2012)
pET24_CGL2	Purification of CGL2	toxic	(Butschi et al, 2010)
pET22b(+)-His8-TEVcs-CCTX2	Purification of CCTX2 (structural studies)	not tested	This study
pET24_CCTX2-D567A	Purification of CCTX2 D567A	toxic	This study
pET24_CCTX2-D572A	Purification of CCTX2 D572A	toxic	This study
pET24_CCTX2-D585A	Purification of CCTX2 D585A	non-toxic up to 500 µg/ml	This study
pET24_CCTX2-K600A	Purification of CCTX2 K600A	toxic	This study
pET24_CCTX2-Q635A	Purification of CCTX2 Q635A	non-toxic up to 100 µg/ml	This study
pET24_CCTX2-D639A	Purification of CCTX2 D639A	toxic	This study
pET24_CCTX2-E656A	Purification of CCTX2 E656A	non-toxic up to 100 µg/ml	This study
pET24_CCTX2-E658A	Purification of CCTX2 E658A	toxic	This study
pET24_CCTX2-R660A	Purification of CCTX2 R660A	non-toxic up to 50 µg/ml	This study
pET24_CCTX2-R685A	Purification of CCTX2 R685A	non-toxic up to 50 µg/ml	This study
pET24_CCTX2-D687A	Purification of CCTX2 D687A	non-toxic	This study
pET24_CCTX2-Q689A	Purification of CCTX2 Q689A	non-toxic	This study
pET24_CCTX2-R724A	Purification of CCTX2 R724A	non-toxic up to 200 µg/ml	This study
pET24_CCTX2-R732A	Purification of CCTX2 R732A	toxic	This study
pET24_CCTX2-T739A	Purification of CCTX2 T739A	non-toxic up to 500 µg/ml	This study
pET24_CCTX2-N767A	Purification of CCTX2 N767A	toxic	This study
pET24_CCTX2-DxQ	Purification of CCTX2 D687A, Q689A double mutant	non-toxic	This study
pET24_CCTX2-RxDxQ	Purification of CCTX2 R685A, D687A, Q689A triple mutant	non-toxic	This study
pET24_CCTX2-DDDxL	Purification of CCTX2 D572A, D573A, D574A, L576A quadruple mutant	non-toxic up to 100 µg/ml	This study
pET24_CCTX2-KR	Purification of CCTX2 E595A, K596A, R597A triple mutant	non-toxic up to 100 µg/ml	This study

Appendix Table S10. ***Caenorhabditis spp.*** **and** ***Escherichia coli*** **strains used in this study**

Strain name	Genotype	Feature	Reference
C. elegans			
VC424(BEC-1)	*bec-1(ok700) IV/nT1 [qIs51]*	*bec-1* (VPS-34-recruiting protein) deletion mutant balanced by GFP-marked translocation	CGC
HY494(BRE-2)	*bre-2(ye31)*	*bre-2* (β1,3 galactosyltransferase) deletion mutant	CGC
HY483(BRE-3)	*bre-3(ye26)*	*bre-3* (β1,4 mannosyltransferase) deletion mutant	CGC
HY498(BRE-5)	*bre-5(ye17)*	*bre-5* (β1,3 *N*-acetylglucosamine transferase) deletion mutant	CGC
HY485(BRE-4)	*bre-4(ye27)*	*bre-4* (β1, 4 *N*-acetylgalactosamine transferase) deletion mutant	CGC
GK70	*dkIs37[P_{act-5}::GFP:pgp-1]*	PGP-1 fused to GFP	CGC
N2 (Bristol type)		Wild type	CGC
RT1315	*pwIs503[vha- 6p::mans::GFP +Cbr-unc-119(+)*	MANS (α-Mannosidase II) fused to GFP	CGC
RT311	*pwIs69[vha6p::GFP::rab-11 + unc-119(+)]*	RAB-11 fused to GFP	CGC
RT327	*pwIs72[vha-6p::GFP::rab-5 + Cbr-unc-199(+)]*	RAB-5 fused to GFP	CGC
VC625(VPS-52)	*vps-52(ok853)*	*vps-52* (GARP complex) deletion mutant	CGC
VC985(VPS-54)	*vps-54(ok1463) V*	*vps-54* (GARP complex) deletion mutant	CGC
C. briggsae			
AF16			CGC
C. tropicalis			
JU1373			CGC
E. coli			
DH5α	F^- *φ80lacZΔM15 Δ(lacZYA-argF)U169 recA1 endA1 hsdR17(rK⁻, mK+) phoA supE44 λ⁻ thi-1 gyrA96 relA1*	Cloning	Thermo Fisher Scientific
Turbo	F’ *proA⁺B⁺ lacI*^{q} Δ *lacZ* M15/ *fhuA2 Δ(lac-proAB) glnV gal R(zgb- 210*::*Tn10*)Tet^S *endA1 thi-1 Δ(hsdS-mcrB)5*	Cloning	New England Biolabs
OP50	*ura-*	*C. elegans* feeding	CGC
BL21(DE3)	*F^- ompT hsdSb($r_B^-m_B^-$) gal dcm* (DE3) F` (traD36, *pro*AB+ *lac*Iq, Δ(*lac*Z)M15)	Protein production	New England Biolabs
C41(DE3)	F^- *ompT hsd*S_B (r_B^- m_B^-) *gal dcm* Δ *AcrB* (DE3)	Protein production	Sigma-Aldrich Merck

Note: CGC: Caenorhabditis Genomics Center (CGC), University of Minnesota, Minneapolis USA

Appendix Table S11. Selected primers used in this study

Primer	Sequence	Usage
CCTX2for_NdeI<br>CCTX2rev_NotI	GGAGTCGGCATATGGCTCTCAACGAAGGTG<br>GAATAGCGGCCGCCTACAACTCGGAGTGCTTG	Cloning of CCTX2 cDNA
CCTX1for_His_NdeI<br><br>CCTX1rev_NotI	GCCCGCCATATGCATCATCATCATCATCATTCTATCTCCGAAGGCGTTT ACTGG<br>GTAATAGCGGCCGCCTACAACTCGGAGTG	Cloning of CCTX1 cDNA
CCTX3for_His_NdeI<br><br>CCTX3rev_NotI	GCCCGGCCATATGCATCATCATCATCATCATTCTCTTTCTCAGGGTGTT TACTGG<br>ATATAGCGGCCGCCTAGAGCTCGGAGTGCTTG	Cloning of CCTX3 cDNA
CCTX2ΔNfor_His_NdeI	CTGGGTACATATGCATCATCATCATCATCATCATCATCCCCTGCCCTCT CCTGGTCC	Cloning of CCTX2ΔN (truncation of the two N-terminal BTF domains)
CCTX2ΔC_rev_NotI	GTGATGCGGCCGCCTAATAGATCAGATCGTAGTCATTGC	Cloning CCTX2ΔC (truncation of the C-terminal DUF domain)
CCTX2ΔHSEL_rev_NotI	GAATAGCGGCCGCCTACTTGATTGCCGAAGTACCGACG	Cloning of CTX2ΔHSEL (truncation of the C-terminal HSEL sequon, CCTX2for_NdeI was used as for primer)
CCTX2_fwd<br>CCTX2_rev<br>pET22_fwd<br>pET22_rev	CTTCCAATCCATGGCTCTCAACGAAGGTG<br>CGATATCCATCTACAACTCGGAGTGCTTG<br>CGAGTTGTAGATGGATATCGGAATTAATTCG<br>TGAGAGCCATGGATTGGAAGTACAAGTTTTC	Cloning of full-length CCTX2 including a N-terminal His_8 tag followed by a TEV cleavage site into pET22b

Appendix References

Abramson J, Adler J, Dunger J, Evans R, Green T, Pritzel A, Ronneberger O, Willmore L, Ballard AJ, Bambrick J et al (2024) Accurate structure prediction of biomolecular interactions with AlphaFold 3. *Nature* 630: 493–500

Azzi A, Wang T, Zhu D-W, Zou YS, Liu W-Y, Lin S-X (2009) Crystal structure of native cinnamomin isoform III and its comparison with other ribosome inactivating proteins. *Proteins* 74: 250–255

Best HL, Williamson LJ, Lipka-Lloyd M, Waller-Evans H, Lloyd-Evans E, Rizkallah PJ, Berry C (2022) The crystal structure of *Bacillus thuringiensis* Tpp80Aa1 and its interaction with galactose-containing glycolipids. *Toxins* 14: 863

Broom A, Doxey AC, Lobsanov YD, Berthin LG, Rose DR, Howell PL, McConkey BJ, Meiering EM (2012) Modular evolution and the origins of symmetry: reconstruction of a three-fold symmetric globular protein. *Structure* 20: 161–171

Butschi A, Titz A, Wälti MA, Olieric V, Paschinger K, Nöbauer K, Guo X, Seeberger PH, Wilson IBH, Aebi M et al (2010) *Caenorhabditis elegans N*-glycan core ß-galactoside confers sensitivity towards nematotoxic fungal galectin CGL2. *PLoS Pathog* 6: e1000717

Carrère-Kremer S, Blaise M, Singh VK, Alibaud L, Tuaillon E, Halloum I, van de Weerd R, Guérardel Y, Drancourt M, Takiff H et al (2015) A new dehydratase conferring innate resistance to thiacetazone and intra-amoebal survival of *Mycobacterium smegmatis*. *Mol Microbiol* 96: 1085–1102

Clout NJ, Tisi D, Hohenester E (2003) Novel fold revealed by the structure of a FAS1 domain pair from the insect cell adhesion molecule fasciclin I. *Structure* 11: 197–203

de Guillen K, Mammri L, Gracy J, Padilla A, Barthe P, Hoh F, Lahfa M, Rouffet J, Petit-Houdenot Y, Kroj T et al (2025) *Zymoseptoria tritici* effectors structurally related to killer proteins UmV-KP4 and UmV-KP6 inhibit fungal growth, and define extended protein families in fungi. *Mol Plant Pathol* 26: e70141

Faham S, Hileman RE, Fromm JR, Linhardt RJ, Rees DC (1996) Heparin structure and interactions with basic fibroblast growth factor. *Science* 271: 1116–1120

Fujimoto Z, Kaneko S, Kuno A, Kobayashi H, Kusakabe I, Mizuno H (2004) Crystal structures of decorated xylooligosaccharides bound to a family 10 xylanase from *Streptomyces olivaceoviridis* E-86. *J Biol Chem* 279: 9606–9614

Fujishiro T, Tamura H, Schick M, Kahnt J, Xie X, Ermler U, Shima S (2013) Identification of the HcgB enzyme in [Fe]-hydrogenase-cofactor biosynthesis. *Angew Chem Int Ed Engl* 52: 12555–12558

Grahn E, Askarieh G, Holmner A, Tateno H, Winter HC, Goldstein IJ, Krengel U (2007) Crystal structure of the *Marasmius oreades* mushroom lectin in complex with a xenotransplantation epitope. *J Mol Biol* 369: 710–721

Grahn EM, Winter HC, Tateno H, Goldstein IJ, Krengel U (2009) Structural characterization of a lectin from the mushroom *Marasmius oreades* in complex with the blood group B trisaccharide and calcium. *J Mol Biol* 390: 457–466

Greber BJ, Boehringer D, Leibundgut M, Bieri P, Leitner A, Schmitz N, Aebersold R, Ban N (2014) The complete structure of the large subunit of the mammalian mitochondrial ribosome. *Nature* 515: 283–286

Gu F, Khimani A, Rane SG, Flurkey WH, Bozarth RF, Smith TJ (1995) Structure and function of a virally encoded fungal toxin from *Ustilago maydis*: a fungal and mammalian Ca^{2+} channel inhibitor. *Structure* 3: 805–814

Halavaty AS, Moffat K (2007) N- and C-terminal flanking regions modulate light-induced signal transduction in the LOV2 domain of the blue light sensor phototropin 1 from *Avena sativa*. *Biochemistry* 46: 14001–14009

Höpfner D, Cichy A, Pogenberg V, Krisp C, Mezouar S, Bach NC, Grotheer J, Zarza SM, Martinez E, Bonazzi M et al (2023) The DNA-binding induced (de)AMPylation activity of a *Coxiella burnetii* Fic enzyme targets histone H3. *Commun Biol* 6: 1124

Hou Y-J, Guo Y, Li D-F, Zhou N-Y (2021) Structural and biochemical analysis reveals a distinct catalytic site of salicylate 5-monooxygenase NagGH from Rieske dioxygenases. *Appl Environ Microbiol* 87

Hu Y-P, Zhong Y-Q, Chen Z-G, Chen C-Y, Shi Z, Zulueta MM, Ku C-C, Lee P-Y, Wang C-C, Hung S-C (2012) Divergent synthesis of 48 heparan sulfate-based disaccharides and probing the specific sugar-fibroblast growth factor-1 interaction. *J Am Chem Soc* 134: 20722–20727

Ichinose H, Fujimoto Z, Honda M, Harazono K, Nishimoto Y, Uzura A, Kaneko S (2009) A β-L-arabinopyranosidase from *Streptomyces avermitilis* is a novel member of glycoside hydrolase family 27. *J Biol Chem* 284: 25097–25106

Inoue K, Sobhany M, Transue TR, Oguma K, Pedersen LC, Negishi M (2003) Structural analysis by X-ray crystallography and calorimetry of a haemagglutinin component (HA1) of the progenitor toxin from *Clostridium botulinum*. *Microbiology* 149: 3361–3370

Jiang D, Fan J, Wang X, Zhao Y, Huang B, Liu J, Zhang XC (2012) Crystal structure of 1,3Gal43A, an exo-β-1,3-galactanase from *Clostridium thermocellum*. *J Struct Biol* 180: 447–457

Jurgenson CT, Pollard TD (2015) Crystals of the Arp2/3 complex in two new space groups with structural information about actin-related protein 2 and potential WASP binding sites. *Acta Crystallogr F Struct Biol Commun* 71: 1161–1168

Kadirvelraj R, Grant OC, Goldstein IJ, Winter HC, Tateno H, Fadda E, Woods RJ (2011) Structure and binding analysis of *Polyporus squamosus* lectin in complex with the Neu5Acα2-6Galβ1-4GlcNAc human-type influenza receptor. *Glycobiology* 21: 973–984

Kern O, Valenzuela Leon PC, Gittis AG, Bonilla B, Cruz P, Campos Chagas AC, Ganesan S, Ribeiro JMC, Garboczi DN, Martin-Martin I et al (2021) The structures of two salivary proteins from the West Nile vector *Culex quinquefasciatus* reveal a β-trefoil fold with putative sugar binding properties. *Curr Res Struct Biol* 3: 95–105

Khan F, Kurre D, Suguna K (2020) Crystal structures of a β-trefoil lectin from *Entamoeba histolytica* in monomeric and a novel disulfide bond-mediated dimeric forms. *Glycobiology* 30: 474–488

Kim DE, Chivian D, Baker D (2004) Protein structure prediction and analysis using the *Robetta* server. *Nucleic Acids Res* 32: W526–W531

Kombrink A, Tayyrov A, Essig A, Stöckli M, Micheller S, Hintze J, van Heuvel Y, Dürig N, Lin C-W, Kallio PT et al (2019) ISME J. Supplementary Information 2 (xlsx-file) https://www.nature.com/articles/s41396-018-0293-8#Sec21 [DATASET]

Kwon SC, Nguyen TA, Choi YG, Jo MH, Hohng S, Kim VN, Woo JS (2016) Structure of human DROSHA. *Cell* 164: 81–90

Lannes L, Furman CM, Hickman AB, Dyda F (2023) Zinc-finger BED domains drive the formation of the active *Hermes* transpososome by asymmetric DNA binding. *Nat Commun* 14: 4470

Lansky S, Salama R, Biarnés X, Shwartstein O, Schneidman-Duhovny D, Planas A, Shoham Y, Shoham G (2022) Integrative structure determination reveals functional global flexibility for an ultra-multimodular arabinanase. *Commun Biol* 5: 465

Lee J, Blaber M (2011) Experimental support for the evolution of symmetric protein architecture from a simple peptide motif. *Proc Natl Acad Sci USA* 108: 126–130

Levy S, Allerston C, Liveanu V, Habib MR, Gileadi O, Schuster G (2016) Identification of LACTB2, a metallo-beta-lactamase protein, as a human mitochondrial endoribonuclease. *Nucleic Acids Res* 44: 1813–1832

Li W, Zheng S, Xu X, He J, Jiao X, Wang M, Hu W, Li S, Jiang X, Lim B et al (2025) A conserved adaptor orchestrates co-secretion of synergistic type VI effectors in gut Bacteroidota. *Cell Host Microbe* 33: 1901–1915 e1910

Liebschner D, Afonine PV, Baker ML, Bunkóczi G, Chen VB, Croll TI, Hintze B, Hung L-W, Jain S, McCoy AJ et al (2019) Macromolecular structure determination using X-rays, neutrons and electrons: recent developments in *Phenix*. *Acta Crystallogr D Struct Biol* 75: 861–877

Little MS, Redinbo MR (2018) Crystal structure of the mouse innate immunity factor bacterial permeability-increasing family member A1. *Acta Crystallogr F Struct Biol Commun* 74: 268–276

Lovell SC, Davis IW, Arendall WBI, de Bakker PIW, Word JM, Prisant MG, Richardson JS, Richardson DC (2003) Structure validation by Cα geometry: ϕ,ψ and Cβ deviation. *Proteins* 50: 437–450

Miyakawa T, Miyazono K, Sawano Y, Hatano K, Tanokura M (2009) Crystal structure of ginkbilobin-2 with homology to the extracellular domain of plant cysteine-rich receptor-like kinases. *Proteins* 77: 247–251

Muraguchi H, Umezawa K, Niikura M, Yoshida M, Kozaki T, Ishii K, Sakai K, Shimizu M, Nakahori K, Sakamoto Y et al (2015) NIH Sequence Read Archive (SRA) SRA051294, SRA051421, SRA050788 https://doi.org/10.1371/journal.pone.0141586.s018 [DATASET]

Nakamura T, Tonozuka T, Ide A, Yuzawa T, Oguma K, Nishikawa A (2008) Sugar-binding sites of the HA1 subcomponent of *Clostridium botulinum* type C progenitor toxin. *J Mol Biol* 376: 854–867

Nakamura T, Tonozuka T, Ito S, Takeda Y, Sato R, Matsuo I, Ito Y, Oguma K, Nishikawa A (2011) Molecular diversity of the two sugar-binding sites of the β-trefoil lectin HA33/C (HA1) from *Clostridium botulinum* type C neurotoxin. *Arch Biochem Biophys* 512: 69–77

Nguyen Y, Sugiman-Marangos S, Harvey H, Bell SD, Charlton CL, Junop MS, Burrows LL (2015) *Pseudomonas aeruginosa* minor pilins prime type IVa pilus assembly and promote surface display of the $PilY_1$ adhesin. *J Biol Chem* 290: 601–611

Pan X, Cao D, Xie F, Xu F, Su X, Mi H, Zhang X, Li M (2020) Structural basis for electron transport mechanism of complex I-like photosynthetic NAD(P)H dehydrogenase. *Nat Commun* 11: 610

Pei J, Wagner ND, Zou AJ, Chatterjee S, Borek D, Cole AR, Kim PJ, Basler CF, Otwinowski Z, Gross ML et al (2021) Structural basis for IFN antagonism by human respiratory syncytial virus nonstructural protein 2. *Proc Natl Acad Sci USA* 118

Pellegrini O, Mathy N, Gogos A, Shapiro L, Condon C (2005) The *Bacillus subtilis* ydcDE operon encodes an endoribonuclease of the MazF/PemK family and its inhibitor. *Mol Microbiol* 56: 1139–1148

Peng L, Wang L, Zhang Y, Dong A, Shen W-H, Huang Y (2018) Structural analysis of the arabidopsis AL2-PAL and PRC1 complex provides mechanistic insight into active-to-repressive chromatin state switch. *J Mol Biol* 430: 4245–4259

Pohleven J, Renko M, Magister Š, Smith DF, Künzler M, Štrukelj B, Turk D, Kos J, Sabotič J (2012) Bivalent carbohydrate binding is required for biological activity of *Clitocybe nebularis* lectin (CNL), the *N*,*N*'-diacetyllactosediamine (GalNAcβ1-4GlcNAc, LacdiNAc)-specific lectin from basidiomycete *C. nebularis*. *J Biol Chem* 287: 10602–10612

Renko M, Zupan T, Plaza DF, Schmieder SS, Perisić Nanut M, Kos J, Turk D, Künzler M, Sabotič J (2022) Cocaprins, β-trefoil fold Inhibitors of cysteine and aspartic proteases from *Coprinopsis cinerea*. *Int J Mol Sci* 23

Ruegenberg S, Horn M, Pichlo C, Allmeroth K, Baumann U, Denzel MS (2020) Loss of GFAT-1 feedback regulation activates the hexosamine pathway that modulates protein homeostasis. *Nat Commun* 11: 687

Schubert M, Bleuler-Martinez S, Butschi A, Walti MA, Egloff P, Stutz K, Yan S, Collot M, Mallet JM, Wilson IB et al (2012) Plasticity of the beta-trefoil protein fold in the recognition and control of invertebrate predators and parasites by a fungal defence system. *PLoS Pathog* 8: e1002706

Shaaban M, Chowdhury S, Nolen BJ (2020) Cryo-EM reveals the transition of Arp2/3 complex from inactive to nucleation-competent state. *Nat Struct Mol Biol* 27: 1009–1016

Shahin M, Sheppard D, Raynaud C, Berry J-L, Gurung I, Silva LM, Feizi T, Liu Y, Pelicic V (2023) Characterization of a glycan-binding complex of minor pilins completes the analysis of *Streptococcus sanguinis* type 4 pili subunits. *Proc Natl Acad Sci USA* 120: e2216237120

Skamnaki VT, Peumans WJ, Kantsadi AL, Cubeta MA, Plas K, Pakala S, Zographos SE, Smagghe G, Nierman WC, Van Damme EJM et al (2013) Structural analysis of the *Rhizoctonia solani* agglutinin reveals a domain-swapping dimeric assembly. *FEBS J* 280: 1750–1763

Sulzenbacher G, Roig-Zamboni V, Peumans WJ, Rougé P, Van Damme EJM, Bourne Y (2010) Crystal structure of the GalNAc/Gal-specific agglutinin from the phytopathogenic ascomycete *Sclerotinia sclerotiorum* reveals novel adaptation of a beta-trefoil domain. *J Mol Biol* 400: 715–723

Suzuki R, Fujimoto Z, Kaneko S, Hasegawa T, Kuno A (2018) Enhanced azidolysis by the formation of stable Ser-His catalytic dyad in a glycoside hydrolase family 10 xylanase mutant. *J Appl Glycosci* 65: 1–8

Tayyrov A, Schmieder SS, Bleuler-Martinez S, Plaza DF, Künzler M (2018) ArrayExpress repository MTAB-7005 https://www.ebi.ac.uk/biostudies/ArrayExpress/studies/E-MTAB-7005?query=E-MTAB-7005 [DATASET]

Teh A-H, Kimura M, Yamamoto M, Tanaka N, Yamaguchi I, Kumasaka T (2006) The 1.48 Å resolution crystal structure of the homotetrameric cytidine deaminase from mouse. *Biochemistry* 45: 7825–7833

Tempel W, Liu Z-J, Horanyi PS, Deng L, Lee D, Gary Newton M, Rose JP, Ashida H, Li S-C, Li Y-T et al (2005) Three-dimensional structure of GlcNAcα1-4Gal releasing endo-β-galactosidase from *Clostridium perfringens*. *Proteins* 59: 141–144

Treiber N, Reinert DJ, Carpusca I, Aktories K, Schulz GE (2008) Structure and mode of action of a mosquitocidal holotoxin. *J Mol Biol* 381: 150–159

Vaattovaara A, Brandt B, Rajaraman S, Safronov O, Veidenberg A, Luklová M, Kangasjärvi J, Löytynoja A, Hothorn M, Salojärvi J et al (2019) Mechanistic insights into the evolution of DUF26-containing proteins in land plants. *Commun Biol* 2: 56

Valverde R, Ingram J, Harrison SC (2016) Conserved tetramer junction in the kinetochore Ndc80 complex. *Cell Rep* 17: 1915–1922

van Eerde A, Grahn EM, Winter HC, Goldstein IJ, Krengel U (2015) Atomic-resolution structure of the α-galactosyl binding *Lyophyllum decastes* lectin reveals a new protein family found in both fungi and plants. *Glycobiology* 25: 492–501

Varki A, Cummings RD, Aebi M, Packer NH, Seeberger PH, Esko JD, Stanley P, Hart G, Darvill A, Kinoshita T et al (2015) Symbol nomenclature for graphical representations of glycans. *Glycobiology* 25: 1323–1324

Williams CJ, Headd JJ, Moriarty NW, Prisant MG, Videau LL, Deis LN, Verma V, Keedy DA, Hintze BJ, Chen VB et al (2018) *MolProbity*: More and better reference data for improved all-atom structure validation. *Protein Sci* 27: 293–315

Wohlschlager T, A B, Zurfluh K, Vonesch SC, auf dem Keller U, Gehrig P, Bleuler-Martinez S, Hengartner MO, Aebi M, Kunzler M (2011) Nematotoxicity of Marasmius oreades agglutinin (MOA) depends on glycolipid binding and cysteine protease activity.

Yanez ME, Korotkov KV, Abendroth J, Hol WGJ (2008) The crystal structure of a binary complex of two pseudopilins: EpsI and EpsJ from the type 2 secretion system of *Vibrio vulnificus*. *J Mol Biol* 375: 471–486

Yoshimoto S, Sasahara J, Suzuki A, Kanie J, Koiwai K, Lupas AN, Hori K (2025) Insights into the complex formation of a trimeric autotransporter adhesin with a peptidoglycan-binding periplasmic protein. *Cell Surf* 14: 100155

Yuan L, Liu Q, Xu L, Wu B, Feng Y (2024) Structural basis of promoter recognition by *Staphylococcus aureus* RNA polymerase. *Nat Commun* 15: 4850

Zhang L, Einsle O (2024) Architecture of the RNF1 complex that drives biological nitrogen fixation. *Nat Chem Biol* 20: 1078–1085

Zhang P, Li K, Yang G, Xia C, Polston JE, Li G, Li S, Lin Z, Yang L-J, Bruner SD et al (2017) Cytotoxic protein from the mushroom *Coprinus comatus* possesses a unique mode for glycan binding and specificity. *Proc Natl Acad Sci USA* 114: 8980–8985

Zhu X, Ye K (2012) Crystal structure of Cmr2 suggests a nucleotide cyclase-related enzyme in type III CRISPR-Cas systems. *FEBS Lett* 586: 939–945

Žurga S, Pohleven J, Renko M, Bleuler-Martinez S, Sosnowski P, Turk D, Künzler M, Kos J, Sabotič J (2014) A novel β-trefoil lectin from the parasol mushroom (*Macrolepiota procera*) is nematotoxic. *FEBS J* 281: 3489–3506
