# Supplementary figures and images for: Structure and function of a fungal AB toxin-like chimerolectin involved in anti-nematode defense
Source: EMBO J. 2026 May 26;45(13):4766–86. doi: 10.1038/s44318-026-00812-1 (PMC13324453; doi:10.1038/s44318-026-00812-1)

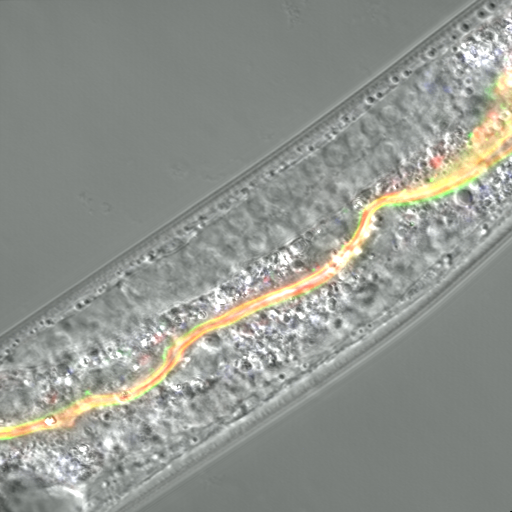

Supplement: Supplementary file 9 — Source data Fig. 1 [file 44318_2026_812_MOESM9_ESM.zip › Figure 1/1DE/Fig1D.tif]

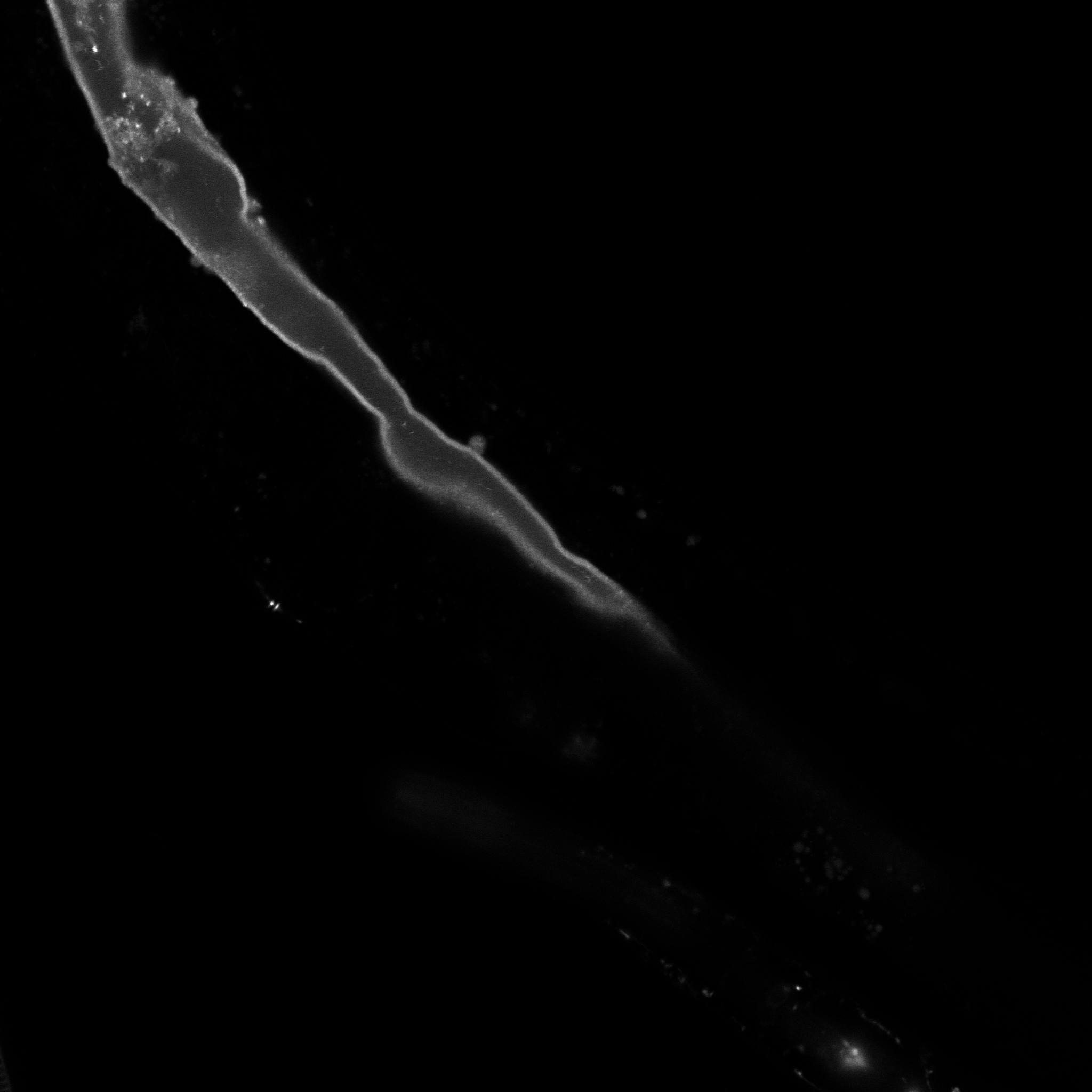

Supplement: Supplementary file 9 — Source data Fig. 1 [file 44318_2026_812_MOESM9_ESM.zip › Figure 1/1DE/Fig1E_16h.tif]

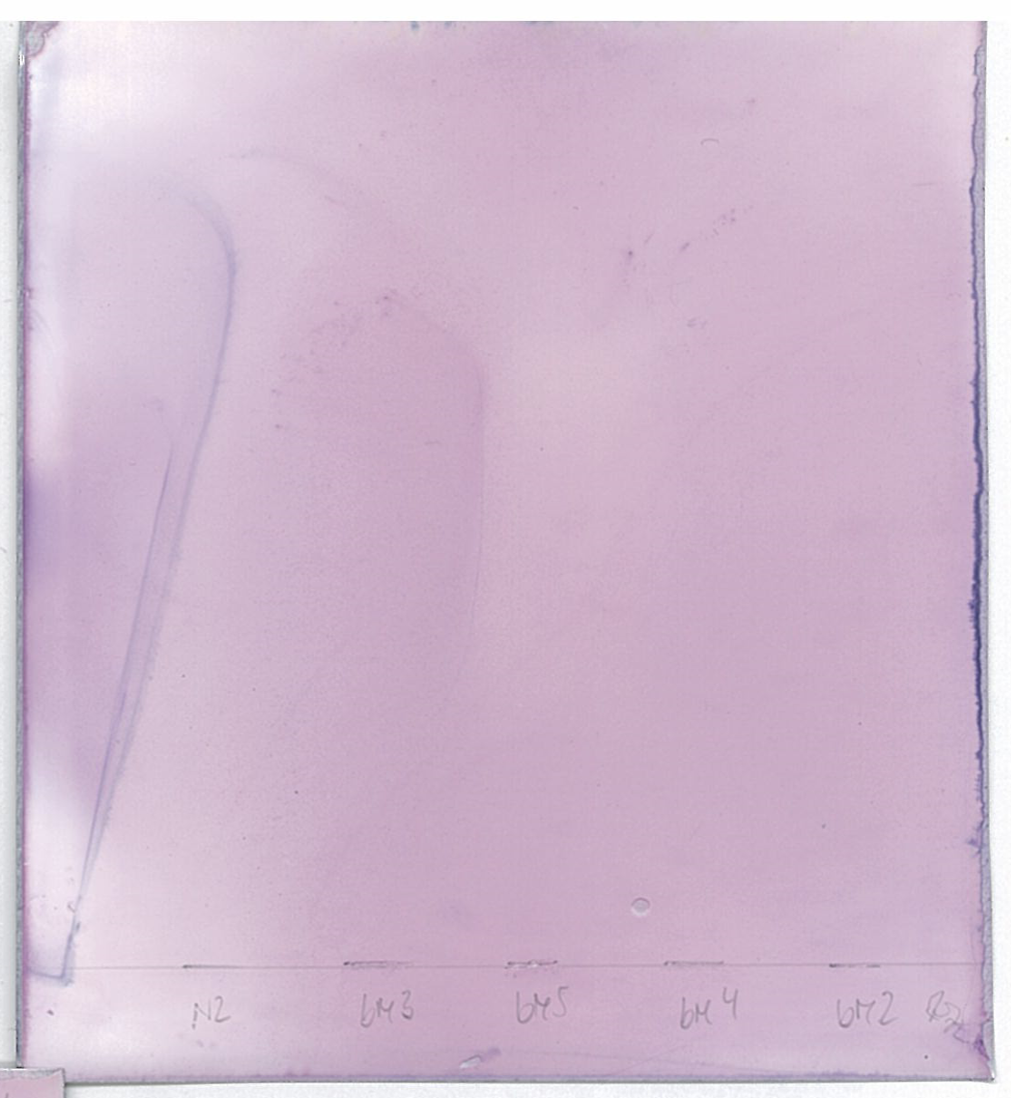

Supplement: Supplementary file 11 — Source data Fig. 5 [file 44318_2026_812_MOESM11_ESM.zip › Figure 5/5C/CCTX2dN.tif]
