# Supplementary material for: Structure and function of a fungal AB toxin-like chimerolectin involved in anti-nematode defense
Source: EMBO J. 2026 May 26;45(13):4766–86. doi: 10.1038/s44318-026-00812-1 (PMC13324453; doi:10.1038/s44318-026-00812-1)
Supplement: Supplementary file 2 — Peer Review File [file 44318_2026_812_MOESM2_ESM.pdf]

THE EMBO JOURNAL

Structure and function of a fungal AB toxin-like chimerolectin involved in anti-nematode defense

Stefanie Schmieder, Gabriele Cordara, Flore Kersten, Kevin Steiner, Clara Samim, David Plaza, Ahmad Ali-Ahmad, Andreas Bøggild, Jesper Lykkegaard Karlsen, Blanka Sokolowska, Thomas Boesen, Ute Krengel, and Markus Künzler

Corresponding author(s): Markus Künzler (mkuenzle@ethz.ch) , Ute Krengel (ute.krengel@kjemi.uio.no), Gabriele Cordara (gabriele.cordara@kjemi.uio.no)

Review Timeline:

Submission Date:	8th Aug 25
Editorial Decision:	27th Oct 25
Revision Received:	27th Feb 26
Editorial Decision:	8th Apr 26
Revision Received:	23rd Apr 26
Accepted:	5th May 26

Editor: Daniel Klimmeck

Transaction Report:

(Note: With the exception of the correction of typographical or spelling errors that could be a source of ambiguity, letters and reports are not edited. Depending on transfer agreements, referee reports obtained elsewhere may or may not be included in this compilation. Referee reports are anonymous unless the Referee chooses to sign their reports.)

Dear Dr Künzler,

Thank you again for the submission of your manuscript (EMBOJ-2025-121470) to The EMBO Journal. Please accept my sincere apologies again for getting back to you with this unusual delay, which is due to protracted referee input at this time as well as detailed discussion on your work in the editorial team. As mentioned earlier, your study was assessed by two reviewers with expertise in structural biology of toxins and microbiology, whose comments are enclosed below.

As you will see from the experts' reports, the referees acknowledge the analysis and potential interest and value of your findings. However, they also express important issues regarding the completeness of your study in supporting your claims, which need to be addressed thoroughly to make them supportive of publication in the EMBO Journal. Further, the reviewers raise a number of issues related to the presentation of the findings, additional controls and improved methods annotation required, statistics applied and overall discussion of related literature, that would need to be conclusively addressed to achieve the level of robustness and clarity needed for The EMBO Journal.

Given the overall interest stated and broader angle of your findings, we are able to invite you to revise your manuscript experimentally to address the referees' comments. I need to stress though that we do require strong support from the referees on a revised version of the study in order to move on to publication of the work.

Please feel free to contact me if you have any questions or need further input on the referee comments.

We generally allow three months as standard revision time. As a matter of policy, competing manuscripts published during this period will not negatively impact on our assessment of the conceptual advance presented by your study. However, we request that you contact the editor as soon as possible upon publication of any related work, to discuss how to proceed. Should you foresee a problem in meeting this three-month deadline, please let us know in advance and we may be able to grant an extension.

When submitting your revised manuscript, please carefully review the instructions below.

Please feel free to approach me any time should you have additional questions related to this.

Thank you for the opportunity to consider your work for publication.

I look forward to your revision.

Kind regards,

Daniel Klimmeck

Daniel Klimmeck, PhD
Senior Editor
The EMBO Journal

Instruction for the preparation of your revised manuscript:

1) a .docx formatted version of the manuscript text (including legends for main figures, EV figures and tables). Please make sure that the changes are highlighted to be clearly visible.

2) individual production quality figure files as .eps, .tif, .jpg (one file per figure).

3) a .docx formatted letter INCLUDING the reviewers' reports and your detailed point-by-point response to their comments. As part of the EMBO Press transparent editorial process, the point-by-point response is part of the Review Process File (RPF), which will be published alongside your paper.

4) a complete author checklist, which you can download from our author guidelines (https://wol-prod-cdn.literatumonline.com/pb-assets/embo-site/Author Checklist%20-%20EMBO%20J-1561436015657.xlsx). Please insert information in the checklist that is also reflected in the manuscript. The completed author checklist will also be part of the RPF.

5) Please note that all corresponding authors are required to supply an ORCID ID for their name upon submission of a revised manuscript.

6) It is mandatory to include a 'Data Availability' section after the Materials and Methods. Before submitting your revision, primary datasets produced in this study need to be deposited in an appropriate public database, and the accession numbers and database listed under 'Data Availability'. Please remember to provide a reviewer password if the datasets are not yet public (see https://www.embopress.org/page/journal/14602075/authorguide#datadeposition).
In case you have no data that requires deposition in a public database, please state so in this section. Note that the Data Availability Section is restricted to new primary data that are part of this study.
*** Note - All links should resolve to a page where the data can be accessed. ***

7) Our journal encourages inclusion of *data citations in the reference list* to directly cite datasets that were re-used and obtained from public databases. Data citations in the article text are distinct from normal bibliographical citations and should directly link to the database records from which the data can be accessed. In the main text, data citations are formatted as follows: "Data ref: Smith et al, 2001" or "Data ref: NCBI Sequence Read Archive PRJNA342805, 2017". In the Reference list, data citations must be labeled with "[DATASET]". A data reference must provide the database name, accession number/identifiers and a resolvable link to the landing page from which the data can be accessed at the end of the reference. Further instructions are available at .

8) At EMBO Press we ask authors to provide source data for the main and EV figures. Our source data coordinator will contact you to discuss which figure panels we would need source data for and will also provide you with helpful tips on how to upload and organize the files.

Numerical data can be provided as individual .xls or .csv files (including a tab describing the data). For 'blots' or microscopy, uncropped images should be submitted (using a zip archive or a single pdf per main figure if multiple images need to be supplied for one panel). Additional information on source data and instruction on how to label the files are available at .

9) We replaced Supplementary Information with Expanded View (EV) Figures and Tables that are collapsible/expandable online (see examples in https://www.embopress.org/doi/10.15252/embj.201695874). A maximum of 5 EV Figures can be typeset. EV Figures should be cited as 'Figure EV1, Figure EV2" etc. in the text and their respective legends should be included in the main text after the legends of regular figures.

- For the figures that you do NOT wish to display as Expanded View figures, they should be bundled together with their legends in a single PDF file called *Appendix*, which should start with a short Table of Content. Appendix figures should be referred to in the main text as: "Appendix Figure S1, Appendix Figure S2" etc. See detailed instructions regarding expanded view here: .

- Additional Tables/Datasets should be labelled and referred to as Table EV1, Dataset EV1, etc. Legends have to be provided in a separate tab in case of .xls files. Alternatively, the legend can be supplied as a separate text file (README) and zipped together with the Table/Dataset file.

10) When assembling figures, please refer to our figure preparation guideline in order to ensure proper formatting and readability in print as well as on screen:
http://bit.ly/EMBOPressFigurePreparationGuideline

Please remember: Digital image enhancement is acceptable practice, as long as it accurately represents the original data and conforms to community standards. If a figure has been subjected to significant electronic manipulation, this must be noted in the figure legend or in the 'Materials and Methods' section. The editors reserve the right to request original versions of figures and the original images that were used to assemble the figure.

11) For data quantification: please specify the name of the statistical test used to generate error bars and P values, the number (n) of independent experiments (specify technical or biological replicates) underlying each data point and the test used to calculate p-values in each figure legend. The figure legends should contain a basic description of n, P and the test applied. Graphs must include a description of the bars and the error bars (s.d., s.e.m.).

Please remember: Digital image enhancement is acceptable practice, as long as it accurately represents the original data and conforms to community standards. If a figure has been subjected to significant electronic manipulation, this must be noted in the figure legend or in the 'Materials and Methods' section. The editors reserve the right to request original versions of figures and the original images that were used to assemble the figure.

Further information is available in our Guide For Authors: https://www.embopress.org/page/journal/14602075/authorguide

We realize that it is difficult to revise to a specific deadline. In the interest of protecting the conceptual advance provided by the work, we recommend a revision within 3 months (25th Jan 2026). Please discuss the revision progress ahead of this time with the editor if you require more time to complete the revisions. Use the link below to submit your revision:

https://emboj.msubmit.net/cgi-bin/main.plex

Referee #1:

The manuscript describes the structure and nematotoxic function of a novel fungal toxin. The protein has dual function with four domains able to bind to glycolipids in the nematode digestive tract, and an additional domain that has no similarity with know ones but is necessary for the toxic activity. The work is of high interest since on one hand it relates on other two-component toxins, and on the other hand it describes a novel domain. The work is thoroughly performed, with full biochemical and structural characterization and biological experimentation in model nematode system.

My main concern is the absence of discussion about the possible oligomerization of the CCTX2 protein. From the purification procedure, and the electron microscopy data, the protein is monomeric in solution with a compact shape. However, the possibility that it oligomerizes when in contact with glycolipid-containing membrane maybe be considered There is an interesting overall structural similarity between CCTX2 and the CEL3 toxin from sea cucumber (doi: 10.1074/jbc.M404065200). Indeed, CEL3 consists of two b-trefoil domains at the N-term and a b-sheet with two helices at the C-term. This latter domain oligomerizes and forms a pore when the lectin domains are in contact with membrane. It would be of interest to discuss the possibility that CCTX2 is a pore-forming toxin and eventually to test its ability of hemolyze red cells with appropriate glycolipids in membrane.

Other points
The original citation for QxW domain in b-trefoil domain is 10.1002/pro.5560050805 ("The (QxW)3 domain: a flexible lectin scaffold" by B. Hazes 1996)

Other lectins from C cinerea appear line 131 in the result section, while it would have been of interest to have them described in the introduction.

Referee #2:

Excellent manuscript by Schmieder et al. provides a detailed study of the novel fungal toxin CCTX2. Such toxins are by their very nature bi-functional as they require both binding to the target cells and (often) enzymatic activity that disrupts them. It is fairly common for such toxins to be multi-protein complexes, yet in some cases of which CCTX2 is an example of, it is a single polypeptide. Authors performed several experiments that convincingly demonstrate that the N-teminal lectin domains of the protein drive binding to cellular glycolipids, and that retrograde trafficking of the toxin is essential for its biological function. Authors did an excellent job identifying potential key residues of the putative enzymatic C-termial domain, yet its specific functionality remains unknown.

As a structural biologist, I find this report fascinating - more often than not these days the putative function of any protein structural element suspected of enzymatic activity can at the very least be implicated by homology modeling. Yet in this case it appears that the key C-terminal domain presents a novel fold, and thus its function cannot be guessed in this way. The possibility that this toxin will have a unique enzymatic mechanism of toxicity is truly exciting.

My recommendation would be to publish this report after some revisions. I provide a number of specific points below, and I do realize that not everything is feasible in a reasonable timeframe. I have been on the receiving end of quite a number of obnoxious reviews that demanded that a multi-year research effort be implemented before some incognito reviewer could

graciously grant a permission to publish our work. I strongly believe that while reviewers (myself in this current scenario) are welcome to recommend any corrections, revisions, or further studies, ultimately it is the editor's responsibility and purview to determine whether any of my suggestions really have to be implemented. With that said - good job and see my comments below.

Minor revisions:
- Toxin is upregulated when nematodes are present. Please comment on any knowledge of the upregulation mechanism (i.e. if it is known how upregulation is triggered by environmental changes)?
- The C-terminal domain having a novel fold is an important conclusion. Could the authors provide information on specifics of how this conclusion was reached, specifically what DALI algorithm returns as the closest structural homologue and why it is not similar enough?
- Admittedly light microscopy of nematodes is not my area of expertise, so this may sound naive. However, are the images shown in Fig.1 of the same worm or different ones? How representative are these pictures and can the "undulation" authors mention as evidence of toxin damage be quantified? Furthermore, why is the data on lethality not shown?
- Toxicity charts are tricky to interpret given repeating colors and overlapping curves. I was initially confused by Fig1 and assumed that CCTX2 is much less toxic than MOA (it being positive control). The difference is in symbols, and it's not jumping off the page. I would leave to authors to come up with improved representation.
- Please specify whether CCTX2 is secreted or consumed by nematodes as they harvest nutrients from fungal cells.
- Surely the glow discharge current was in mA, not uA.
- Physical pixel of Gatan K2 is 5um, not 0.63A - that's the magnified pixel size. In fact, quoting "nominal magnification" is completely pointless - on FEI microscopes those are calibrated to the plane of the CETA box, and if this scope had the usual configuration of the bottom mounted K2, it's way off. Calibrated magnification is thus around 80K assuming a natural pixel, unless data was collected in super-resolution mode. Either way, I am just pointing out that nominal resolution is a completely irrelevant parameter, and just reporting the pixel size and the type of camera is sufficient to figure out the correct magnification value.
Several other issues with data stats. "Electron exposure" is usually listed as "Total dose" - also, the PDB validation report lists a rather different value, please correct as necessary (I suspect it's the validation report value that is wrong). It's unclear what the two extracted particle number values are referring to, please clarify. Symmetry is misspelled. Why are different resolution numbers listed in data collection and refinement sections?
- Please add representative 2D classes from the final stages of the 2D filtering.
- What, if any, steps were taken to clarify the unresolved density? Flex refinement could be very useful in such areas as well as extensive 3D classification (assuming that better resolving these regions is important).
- Validation report lists the projected molecular weight at 35kDa. That can't be right, please find the cause of this error and correct accordingly.
- This is not always fixable, but the FSC curve has a medium resolution drop. This is sometimes indicative of presence of flexible parts in the model and/or significant fraction of poor quality particles. Perhaps more stringent 2D/3D subclassification could have produced higher quality maps. Like with other suggestions here, improving map quality is always desirable, but one has to be reminded that it only makes sense to invest significant time in such optimization if it is expected to improve the model to the point of providing crucial new information.

Suggested possible experiments (minor):

The toxicity curve for CCTX2 is incomplete at the lower concentration, and few more data points would be useful in determining the relative toxicity of different agents. In particular, authors may consider putting more emphasis on the fact that CCTX2 is much more toxic than any other protein investigated, and having a proper estimate of the toxicity "halfpoint" concentrations could help drive that.

While structural homologue search failed, perhaps some lower probability cutoff sequence alignments could provide some guesses on the function of the C-terminal domain? I understand that none of this would constitute proof, but it would be great to have a list of possibilities to look into in the future.

Authors constructed a number of mutants and most of these show reduced toxicity. Some have rather extensive deletions, so it's important to demonstrate that these are stable and properly folded. I understand that authors indicate that all the proteins produced had no solubility issues, but whether their stability is compromised is important to know in order to address whether reduced toxicity can be partially driven by protein unfolding in vivo. In particular, authors indicate that limited proteolysis does not separate the C-terminal domain from the rest of the protein in non-denaturing conditions, so I find it particularly important to compare stability of the full length protein with N- and C-terminal deletions. Nothing fancy, some DSF curves with Tm and physiological temperature deltaG would be sufficient.

It appears that the unusual downsampling of the original micrographs was applied (nothing wrong with that per se). It is quite common to downsample the initial particle stacks to make them smaller and the subsequent 2D classification less memory intensive and faster (which is OK because in cryoSPARC the particles are downsampled even further internally during 2D classification). It is, however, also common to re-extract particles with the full pixel for the final density refinement. This could improve final resolution (whether that is really important - probably not, since it won't alter structural conclusions reached by the

authors).

Suggested possible experiments (major, including any further structural characterization):

Additional density corresponding to bound sugars is indeed likely to be resulting from ligand picked up in purification. Would it be possible to determine the structure of the protein incubated with higher concentration of putative glycolipid targets or their analogs (in ideal world this would be complex with membrane, e.g. with nanodiscs)? To be fair, the impact of such structural characterization might be limited.

I am assuming that no additional density was observed in the putative zinc binding site. Perhaps authors could solve this structure in the presence of zinc to confirm this prediction?

Ribosyltransferase is a common type of enzyme utilized by toxins. Shouldn't authors check if CCTX2 exhibits such activity? At the very least, comment whether the structure indicates the absence of a potential cofactor binding site.

I find the Kex2 cleavage intriguing in structural context. Authors do allude to the possibility that site directed proteolysis could trigger significant reorganization of the C-terminal domain. An excellent and significant improvement to the paper would be to include the structure of the Kex2 cleaved toxin (I assume that it remains assembled into a single particle that will be large enough for cryoEM). I appreciate that this could be what authors are planning for their next manuscript, so they could just state that this is under investigation. But if authors choose to test the toxin for ribosyltransferase activity, perhaps they could check if it is triggered by Kex2 action. Another relevant computational experiment would be to insert a polyalanine linker into the cleavage site and see if alphafold comes up with a different prediction (I don't know whether alphafold allows to designate a cleaved site, so this would be a trick to emulate the same effect).

Referee #1:
The manuscript describes the structure and nematotoxic function of a novel fungal toxin. The protein has dual function with four domains able to bind to glycolipids in the nematode digestive tract, and an additional domain that has no similarity with know ones but is necessary for the toxic activity. The work is of high interest since on one hand it relates on other two-component toxins, and on the other hand it describes a novel domain. The work is thoroughly performed, with full biochemical and structural characterization and biological experimentation in model nematode system.

My **main concern** is the absence of discussion about the possible oligomerization of the CCTX2 protein. From the purification procedure, and the electron microscopy data, the protein is monomeric in solution with a compact shape. However, the possibility that it oligomerizes when in contact with glycolipid-containing membrane maybe be considered. There is an interesting overall structural similarity between CCTX2 and the CEL3 toxin from sea cucumber (doi: 10.1074/jbc.M404065200). Indeed, CEL3 consists of two b-trefoil domains at the N-term and a b-sheet with two helices at the C-term. This latter domain oligomerizes and forms a pore when the lectin domains are in contact with membrane. It would be of interest to discuss the possibility that CCTX2 is a pore-forming toxin and eventually to test its ability of hemolyze red cells with appropriate glycolipids in membrane.
RE: *We thank the reviewer for this interesting suggestion. We performed hemolysis assays with pig and sheep erythrocytes at CCTX2 concentrations of 40 and 400 µg/ml and BSA (at 400 µg/ml) and Triton X-100 (at 1% v/v) as negative and positive controls. In these assays, incubation with CCTX2 did not result in any hemolysis (see panel A of the figure at the end of this paragraph). Since this negative result could also be explained by the absence of the receptor, we performed hemagglutination assays with pig erythrocytes and CCTX2 and MOA (as positive control) at the same toxin concentrations. In these experiments, both CCTX2 and MOA clearly led to hemagglutination (see panel B of the figure at the end of this paragraph). These results suggest that the surface of mammalian erythrocytes displays receptors for both CCTX2 and MOA but that binding of these receptors by the fungal toxins does not lead to cell lysis.*

Figure for review only. A. *Pig blood agglutination assay. Solution of 0.5% pig erythrocytes in PBS was incubated 1:1 for 30min/RT with either [100ug/ml] MOA as positive agglutination ctrl (row A, wells 1-3), [400ug/ml] CCTX2 (row D, wells 4-6) and [40ug/ml] CCTX2 (row E, wells 4-6). CCTX2 agglutinates pig erythrocytes.* ***B.*** *Pig and sheep blood hemolysis assay. Solution of 0.5% pig or sheep erythrocytes in PBS were incubated 1:1 for 1h/RT with [400ug/ml] BSA or PBS as negative ctrls, 1% TritonX-100 in PBS as positive lysis ctrl and [400ug/ml] and [40ug/ml] CCTX2. Erythrocyte solutions were spun down and absorbance of the supernatants were measured at 502nm. No hemolysis detected for CCTX2.*

A

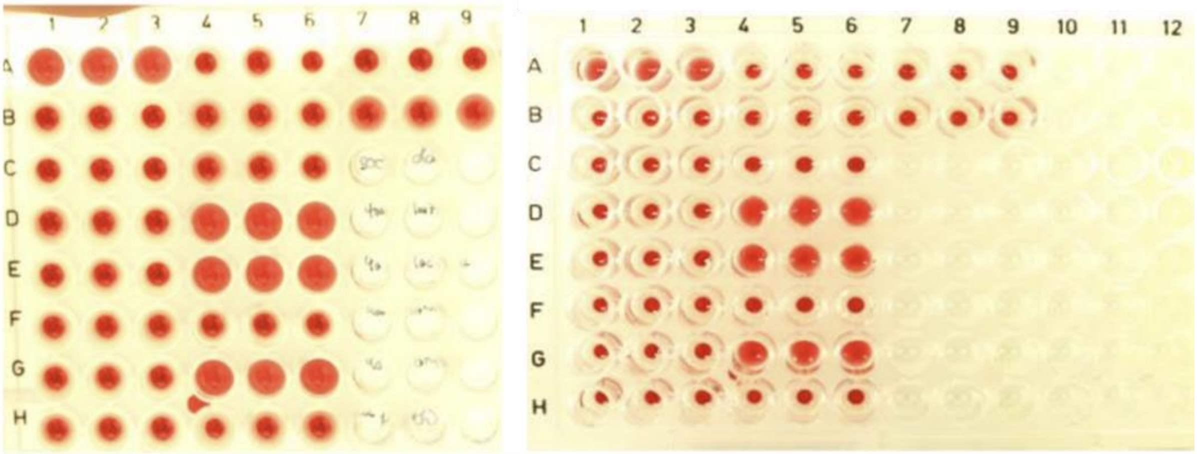

B

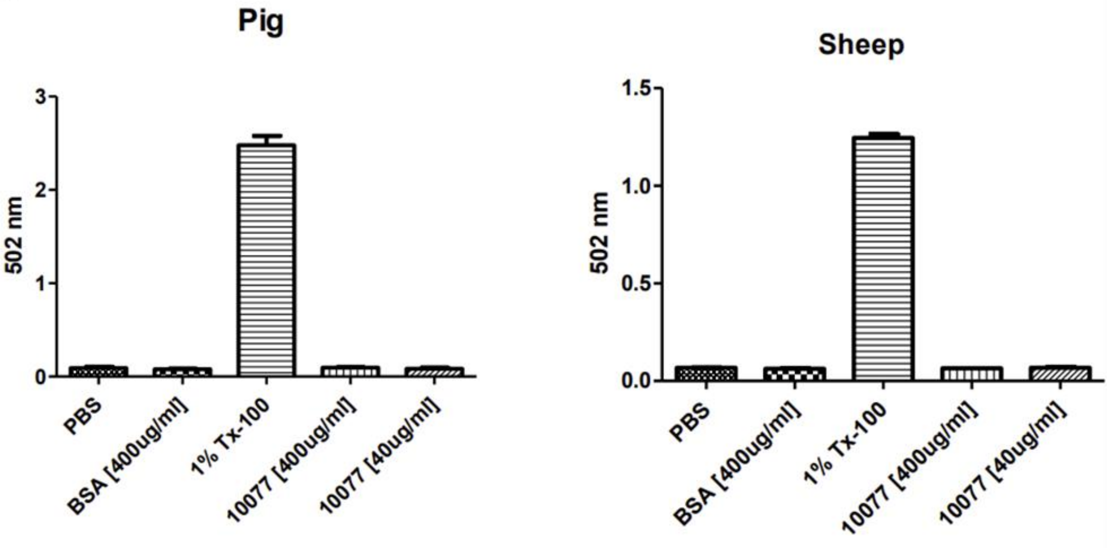

Since it is possible that, in these cells, the receptors are glycoproteins rather than glycolipids, we also incubated Drosophila melanogaster Schneider S2 cells, which contain arthroseries glycosphingolipids, with fluorescently labelled CCTX2. CCTX2 clearly bound to the surface of these cells and was internalized and localized to intracellular compartments, which were also stained with lysotracker. No cell lysis was observed under these conditions, however, suggesting that CCTX2 is not a pore-forming toxin (Supplementary data for review only). Similarly, the endocytosis and retrograde trafficking of fluorescently labelled CCTX2 in C. elegans intestinal epithelial cells and the partial resistance of respective C. elegans mutants argue against CCTX2 being a pore-forming toxin.

Figure for review only. *Internalization of CCTX2 into Schneider S2 cells (derived from Drosophila melanogaster). S2 cells were incubated for 1h/25C with [2.5ug/ml] CCTX2-Alexa488 and lysotrackerRed, fixed in formalin and stained with nuclear dye DAPI (blue). CCTX2 is internalized into S2 cells. White arrow indicates membrane staining of CCTX2.*

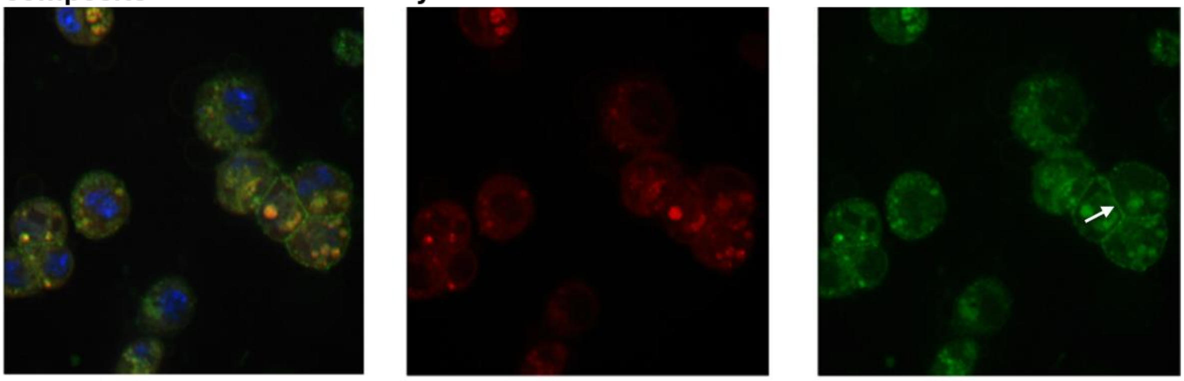

<u>Other points</u>

The original citation for QxW domain in b-trefoil domain is 10.1002/pro.5560050805 ("The (QxW)3 domain: a flexible lectin scaffold" by B. Hazes 1996)

RE: *We are grateful to the reviewer for pointing this out. The citation was replaced in the revised version of the manuscript.*

Other lectins from C cinerea appear line 131 in the result section, while it would have been of interest to have them described in the introduction.

RE: *We prefer to mention the C. cinerea hololectins only at this position because the introduction focuses on the structure and function of chimerolectins from different agaricomycetes and their comparison with bacterial and plant AB toxins.*

Referee #2:

Excellent manuscript by Schmieder et al. provides a detailed study of the novel fungal toxin CCTX2. Such toxins are by their very nature bi-functional as they require both binding to the target cells and (often) enzymatic activity that disrupts them. It is fairly common for such toxins to be multi-protein complexes, yet in some cases of which CCTX2 is an example of, it is a single polypeptide. Authors performed several experiments that convincingly demonstrate that the N-teminal lectin domains of the protein drive binding to cellular glycolipids, and that retrograde trafficking of the toxin is essential for its biological function. Authors did an excellent job identifying potential key residues of the putative enzymatic C-termial domain, yet its specific functionality remains unknown.

As a structural biologist, I find this report fascinating - more often than not these days the putative function of any protein structural element suspected of enzymatic activity can at the very least be implicated by homology modeling. Yet in this case it appears that the key C-terminal domain presents a novel fold, and thus its function cannot be guessed in this way. The possibility that this toxin will have a unique enzymatic mechanism of toxicity is truly exciting.

My recommendation would be to publish this report after some revisions. I provide a number of specific points below, and I do realize that not everything is feasible in a reasonable timeframe. I have been on the receiving end of quite a number of obnoxious

reviews that demanded that a multi-year research effort be implemented before some incognito reviewer could graciously grant a permission to publish our work. I strongly believe that while reviewers (myself in this current scenario) are welcome to recommend any corrections, revisions, or further studies, ultimately it is the editor's responsibility and purview to determine whether any of my suggestions really have to be implemented. With that said - good job and see my comments below.

Minor revisions:

- Toxin is upregulated when nematodes are present. Please comment on any knowledge of the upregulation mechanism (i.e. if it is known how upregulation is triggered by environmental changes)?

RE: *The trigger for the induction of the antinematode defense is still unknown and under investigation in the Künzler laboratory. Thus far, it is known that active feeding by the fungivorous nematode is needed for induction (Plaza et al 2025 DOI: 10.1534/g3.115.023069). Moreover, preliminary experiments using FluidFM as a proxy for the nematode stylet suggest that the drop of the hyphal turgor pressure caused by active feeding (sucking) might act as trigger (Guillaume-Gentil et al 2022 DOI: 10.1038/s42003-022-03127-z). Current experiments aim at confirming latter hypothesis by knocking out components of the cell wall integrity and osmosensing and -regulating pathways in C. cinerea and testing the mutants for defense (toxin) induction. A respective sentence was added to the Discussion.*

- The C-terminal domain having a novel fold is an important conclusion. Could the authors provide information on specifics of how this conclusion was reached, specifically what DALI algorithm returns as the closest structural homologue and why it is not similar enough?

RE: *The result from the last DALI search using the DUF domain, run a little over a year ago, reported a very short list of hits (< 10), none of which bore any significant resemblance to the DUF domain fold (i.e. the match was limited to a single α-helix, or a couple of strands of β-sheet). A similar result was obtained from FoldSeek runs. However, repeating the search with the latest release of DALI yielded a different output, with weak structural matches for the fold architecture (that is, a central β-sheet supported by two α-helices, arranged as observed in CCTX2 DUF domain). Even more interesting results were obtained by running the DALI search using the core portion of the DUF domain, providing some further insights that may be used as leads in future investigations. The manuscript was modified to include this information in the Results section (pp. 7-8), Discussion (pp. 16) as well as in additional panels in Figure 3 (panels D and E), Appendix Figures S7 and S8 and Supplementary Tables S7 and S8.*

- Admittedly light microscopy of nematodes is not my area of expertise, so this may sound naive. However, are the images shown in Fig.1 of the same worm or different ones? How representative are these pictures and can the "undulation" authors mention as evidence of toxin damage be quantified? Furthermore, why is the data on lethality not shown?

RE: *Pictures in Fig. 1D are of the same worm, pictures in Fig. 1E are of different worms but representative for the population. In order to improve clarity, we added a respective explanation to the figure legend. The assessment of the dilatation (undulation) of the intestinal lumen is rather tedious (fluorescence microscopy) and difficult to quantify and, thus, we prefer the assessment of larval stages (by counting) for quantification of toxin damage. For the same reason , the straightness of adult worms which is a sign of death for nematodes. The lethality statement is based on the fact that, in feeding experiments with adult worms and CCTX2-expressing E. coli BL21 cells, many of the worms ended up to be immotile and completely straight (Exemplary picture at the end of this paragraph). This phenotype, which is also difficult to quantify, is a recognized sign of death for nematodes.*

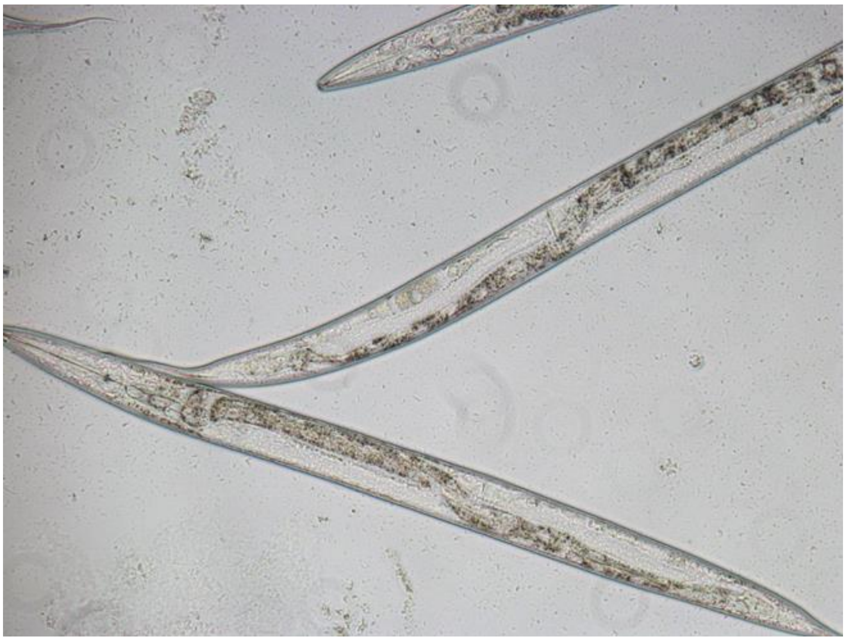

Figure for review only. *DIC Micrographs of L4 larvae (adults) of C. elegans N2 which were fed for 16h with E. coli BL21 expressing CCTX2.*

Toxicity charts are tricky to interpret given repeating colors and overlapping curves. I was initially confused by Fig1 and assumed that CCTX2 is much less toxic than MOA (it being positive control). The difference is in symbols, and it's not jumping off the page. I would leave to authors to come up with improved representation.
RE: *In order to improve clarity and avoid misunderstandings, we modified the figure legend, which now reads: "Nematotoxicity of the paralogous CCTX1, CCTX2 and CCTX3, measured by assessing the percentage of L1 larvae reaching adulthood. Development of C. elegans N2 larvae was arrested at the L1 stage in the presence of either of the three proteins at concentrations >10 µg/ml. This toxicity is higher than the one of the MOA chimerolectin which was included as positive control. BSA served as negative control. Data points with error bars indicate means of N = 4 biological replicates with standard error of the mean (SEM). Overlapping data points were nudged by +/- 2 data units in Y for better readability. For the same reason, the data points were connected despite the fact that no data points in between*

were determined." We hope that this helps to interpret the figure. To us, the presentation is sufficiently clear and we cannot think of any clearer way to present it, but we would be happy for suggestions.

- Please specify whether CCTX2 is secreted or consumed by nematodes as they harvest nutrients from fungal cells.

RE: *The CCTX2 protein does not contain any signal peptide for secretion and is, thus, presumed to be produced and kept in the fungal cytoplasm. Thus, it is taken up by the sucking fungivorous nematodes and ending up in the nematode digestive tract. Similarly, CCTX is produced and kept in the cytoplasm of the heterologous host E. coli, taken up together with the bacterial cells by the swallowing bacterivorous nematode C. elegans and released by disruption of the bacterial cells in the digestive tract of the nematode. This defense and intoxication mechanism is explained in lines 57 – 60 of the introduction. We amended these sentences to make this mechanism more prominent.*

- Surely the glow discharge current was in mA, not uA.

RE: *Corrected in the revised manuscript.*

- Physical pixel of Gatan K2 is 5um, not 0.63A - that's the magnified pixel size. In fact, quoting "nominal magnification" is completely pointless - on FEI microscopes those are calibrated to the plane of the CETA box, and if this scope had the usual configuration of the bottom mounted K2, it's way off. Calibrated magnification is thus around 80K assuming a natural pixel, unless data was collected in super-resolution mode. Either way, I am just pointing out that nominal resolution is a completely irrelevant parameter, and just reporting the pixel size and the type of camera is sufficient to figure out the correct magnification value.

RE: *The sentence has been edited to take into account the comments of Referee #2. The new sentence reads "7,500 movies were collected on a FEI Titan Krios (Thermo Scientific) operating at 300 kV and equipped with a Gatan K2 camera, at a magnification of 215,000 x and with a calibrated pixel size of 0.63 Å".*

- Several other issues with data stats. "Electron exposure" is usually listed as "Total dose" - also, the PDB validation report lists a rather different value, please correct as necessary (I suspect it's the validation report value that is wrong).

RE: *The text and deposition data have been edited.*

- It's unclear what the two extracted particle number values are referring to, please clarify.

RE: *We have rewritten the relevant part to clarify how the final 204,000 particles were processed, specifically highlighting the non-uniform refinement strategy and resolution validation in CryoSPARC. The phrase " 204,000 particles were selected from two rounds of heterogeneous refinement." has been changed to "A final stack of 204,000 particles was selected for non-uniform refinement in CryoSPARC v3.1. Global resolution was determined based on the gold-standard Fourier Shell Correlation (FSC) 0.143 cut-off using two*

independently refined half-maps (Fig. S4B). Local resolution estimates were calculated using CryoSPARC (FigS4C). The final map was locally filtered and sharpened using the overall B-factor determined during the refinement step."

- Symmetry is misspelled.

RE: *Corrected in the revised table.*

- Why are different resolution numbers listed in data collection and refinement sections?

RE: *These were calculated using the validation tool of the Phenix software suite. We acknowledge, though, that the information is redundant (the resolution was estimated in CryoSPARC) and including it would just generate confusion, so we removed that section from Table S2.*

- Please add representative 2D classes from the final stages of the 2D filtering.

RE: *We have now included representative 2D classes in Figure S4C.*

- What, if any, steps were taken to clarify the unresolved density? Flex refinement could be very useful in such areas as well as extensive 3D classification (assuming that better resolving these regions is important).

RE: *We appreciate the reviewer's suggestion to test Flex refinement/3D classification to resolve the flexible regions. While we acknowledge the potential utility of these tools, we believe that the current map quality and resolution are sufficient to support the structural conclusions drawn in this study. Given the inherent flexibility of these domains, further computational processing is unlikely to yield significantly interpretable density that would alter our biological interpretation.*

- Validation report lists the projected molecular weight at 35kDa. That can't be right, please find the cause of this error and correct accordingly.

RE: *We thank the reviewer for pointing this out: the wrong estimate stems from setting a cryo-EM map contour level of 0.489 during deposition, which led to an inconsistent volume-based estimate of the protein molecular weight. The contour level has now been adjusted to 0.125, yielding an isosurface enclosing a volume of 103 700 $Å^3$. This is approximately consistent with a protein of 88.8 kDa (assuming V = 1.21 x MW), which corresponds to the molecular weight of CCTX2.*

- This is not always fixable, but the FSC curve has a medium resolution drop. This is sometimes indicative of presence of flexible parts in the model and/or significant fraction of poor quality particles. Perhaps more stringent 2D/3D subclassification could have produced higher quality maps. Like with other suggestions here, improving map quality is always desirable, but one has to be reminded that it only makes sense to invest significant time in such optimization if it is expected to improve the model to the point of providing crucial new information.

RE: We are aware that reprocessing the data, especially using the latest iteration of CryoSPARC, would yield a higher-quality map. However, as we already stated about the request to perform Flex refinement, further computational processing is unlikely to yield a map that would alter our biological interpretation.

Suggested possible experiments (**minor**):

The toxicity curve for CCTX2 is incomplete at the lower concentration, and few more data points would be useful in determining the relative toxicity of different agents. In particular, authors may consider putting more emphasis on the fact that CCTX2 is much more toxic than any other protein investigated, and having a proper estimate of the toxicity "halfpoint" concentrations could help drive that.

***RE:** This point is well taken, but, unfortunately, we do not have sufficient toxicity data for other purified protein toxins. The TC50 value of CCTX2 compiled from all our toxicity assays with this protein toxin is 5.5 µg/ml (see figure at the end of this paragraph). We cannot compare this value with the ones of other protein toxins, however, since we investigated their nematoxicity mainly by feeding toxin-expressing E. coli BL21 cells rather than purified protein together with 'empty' E. coli BL21 cells. From the few available datapoints for MOA shown in Fig. 1C, one can conclude that the TC50 of this protein toxin must be >20 µg/ml i.e. that CCTX2 is more toxic than MOA.*

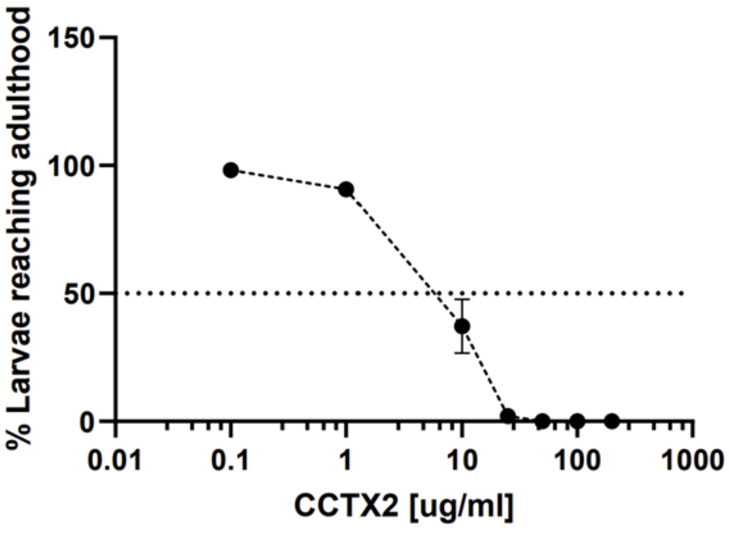

***Figure for review only.** Compiled toxicity data over several different experiments for CCTX2. TC50 is around [5.5ug/ml] CCTX2.*

While structural homologue search failed, perhaps some lower probability cutoff sequence alignments could provide some guesses on the function of the C-terminal domain? I understand that none of this would constitute proof, but it would be great to have a list of possibilities to look into in the future.

***RE:** We lowered the cutoff of our BLASTP searches of the CCTX2 DUF (C-terminal) domain in the NCBI database to 20% identity but could still not detect any protein with annotated*

function that could provide us with a hint towards its function. However, as mentioned in response to a previous comment, a very recent DALI search for structural homologues yielded hits consistent with a role of the DUF domain in fungal host defense.

Authors constructed a number of mutants and most of these show reduced toxicity. Some have rather extensive deletions, so it's important to demonstrate that these are stable and properly folded. I understand that authors indicate that all the proteins produced had no solubility issues, but whether their stability is compromised is important to know in order to address whether reduced toxicity can be partially driven by protein unfolding in vivo. In particular, authors indicate that limited proteolysis does not separate the C-terminal domain from the rest of the protein in non-denaturing conditions, so I find it particularly important to compare stability of the full length protein with N- and C-terminal deletions. Nothing fancy, some DSF curves with Tm and physiological temperature deltaG would be sufficient.
***RE:** We acknowledge this reasonable suggestion by the reviewer. Since the truncated versions of the CCTX2 protein that were used for the toxicity experiments were not available any more and, thus, would have had to be newly produced, we were unfortunately unable to perform this experiment within the given time frame. We hope that the solubility of the proteins is sufficient proof for the proper folding of the proteins. The most relevant assessment of protein stability would anyway be in the intestinal tract of the nematode, which is hard to assess by in vitro methods.*

- It appears that the unusual downsampling of the original micrographs was applied (nothing wrong with that per se). It is quite common to downsample the initial particle stacks to make them smaller and the subsequent 2D classification less memory intensive and faster (which is OK because in cryoSPARC the particles are downsampled even further internally during 2D classification). It is, however, also common to re-extract particles with the full pixel for the final density refinement. This could improve final resolution (whether that is really important - probably not, since it won't alter structural conclusions reached by the authors).
***RE:** Particles were extracted with a box size of 480 pixels and downsampled to 256 pixels, corresponding to a final pixel size of 1.218 Å. This sampling rate imposes a theoretical resolution limit (Nyquist frequency) of approximately 2.44 Å (Shannon, 1949). Since the final reconstruction reached 3.2 Å resolution - well below the Nyquist limit - the downsampling step was not the resolution-limiting factor, which we attribute instead to inherent conformational flexibility of the complex.*

<u>Suggested possible experiments (**major, including any further structural characterization):**</u>
Additional density corresponding to bound sugars is indeed likely to be resulting from ligand picked up in purification. Would it be possible to determine the structure of the protein incubated with higher concentration of putative glycolipid targets or their analogs (in ideal world this would be complex with membrane, e.g. with nanodiscs)? To be fair, the impact of such structural characterization might be limited.
***RE:** We thank the reviewer for bringing up this experimental idea. We aim at solving the*

structure of complexes between CCTX2 and parts of the GSL glycan core including the LacdiNAc epitope in the near future. Such experiments are, however, time-consuming and beyond the time frame of the revision of the current manuscript.

I am assuming that no additional density was observed in the putative zinc binding site. Perhaps authors could solve this structure in the presence of zinc to confirm this prediction?
RE: *No density that could be clearly attributed to a metal ion could be discerned at the proposed zinc-binding site. However, cysteines are commonly found as ligands at zinc-coordinating sites in proteins, as extensively described by e.g. Wolfgang Maret and coworkers in several papers (e.g. Maret W. and Li Y., 2009, Chem. Rev., 109, 4682–4707 or Maret W., 2005, J. Trace Elem. Med. Biol., 19, 7-2). Both the presence of three cysteine residues at the same location and their geometric arrangement strongly suggest that BTF domain 4 hosts a Zn^{2+}-binding site. Reinforcing that notion, AlphaFold3 folding simulations run with the inclusion of a zinc ion resulted in the placement of Zn^{2+} at the proposed location. Finally, it is worth mentioning that the cysteine arrangement found at the proposed zinc-binding site of CCTX2 has been observed in nature, e.g. in cytidine deaminases (PDB ID: 2FR6). This was addressed by amending the respective text of the revised manuscript and by adding an additional Supplementary Figure S6 to the Appendix.*

Ribosyltransferase is a common type of enzyme utilized by toxins. Shouldn't authors check if CCTX2 exhibits such activity? At the very least, comment whether the structure indicates the absence of a potential cofactor binding site.
RE: *We already tested CCTX2 for binding to NAD, both experimentally and by modelling, but could not detect any convincing evidence for binding (data not shown). It is planned to test CCTX2, in its native and Kex2-cleaved form (see comment below), for enzymatic activities, including ADP-ribosyltransferase, UDP-glucosyltransferase, D-amidase, protease, RNase, RNA N-glucosidase, DNase, adenylate cyclase, etc. However, these assays are challenging, because the substrates of some of these activities, e.g. ADP-ribosyltransferase, could be toxin-specific, and, thus, beyond the scope of this study.*
We hoped to get clues regarding the enzymatic activity of the C-terminal domain from its structure. A DALI search did not reveal any homology to ribosyltransferases; however, it returned several hits of proteins binding to a variety of different ligands, including FAD and ATP (see Appendix Fig. S8).

I find the Kex2 cleavage intriguing in structural context. Authors do allude to the possibility that site directed proteolysis could trigger significant reorganization of the C-terminal domain. An excellent and significant improvement to the paper would be to include the structure of the Kex2 cleaved toxin (I assume that it remains assembled into a single particle that will be large enough for cryoEM). I appreciate that this could be what authors are planning for their next manuscript, so they could just state that this is under investigation. But if authors choose to test the toxin for ribosyltransferase activity, perhaps they could check if it is triggered by Kex2 action. Another relevant computational experiment would be

to insert a polyalanine linker into the cleavage site and see if alphafold comes up with a different prediction (I don't know whether alphafold allows to designate a cleaved site, so this would be a trick to emulate the same effect).
RE: *We agree that the activation of the enzymatic activity of CCTX2 by Kex2 cleavage is an interesting hypothesis. As mentioned above, we are planning, as a continuation of this study, to test this hypothesis at functional level, by assaying CCTX2 in its native and Kex2-cleaved form, for various known enzymatic activities, including ADP-ribosyltransferase activity. At structural level, we also aim at determining the cryo-EM structure of the Kex2-cleaved protein. We have now, however, performed AlphaFold3 predictions of CCTX2, both cleaved by Kex2 and with a polyalanine or polyglycine linker inserted into the Kex2 cleavage site (60 alanines or glycines, respectively). The insertion of a polyalanine linker turned out to be problematic, as AlphaFold models it as an extensive α-helix, which imposes unnatural constraints on the final structure; however, switching to a polyglycine linker proved to be a viable option. Neither modelling CCTX2 starting from Kex2 cleavage products nor the linker insertion significantly altered the overall fold of the C-terminal domain, as shown in the figure attached at the end of this paragraph. Quite interestingly, though, the cleaved CCTX2 AlphaFold3 simulation consistently folds showing a small conformational change, with the last ten C-terminal amino acids shifting towards the solvent-exposed surface of the DUF domain β-sheet. This finding has now been included in the manuscript and visualized in an additional Supplementary Figure S12 in the Appendix.*

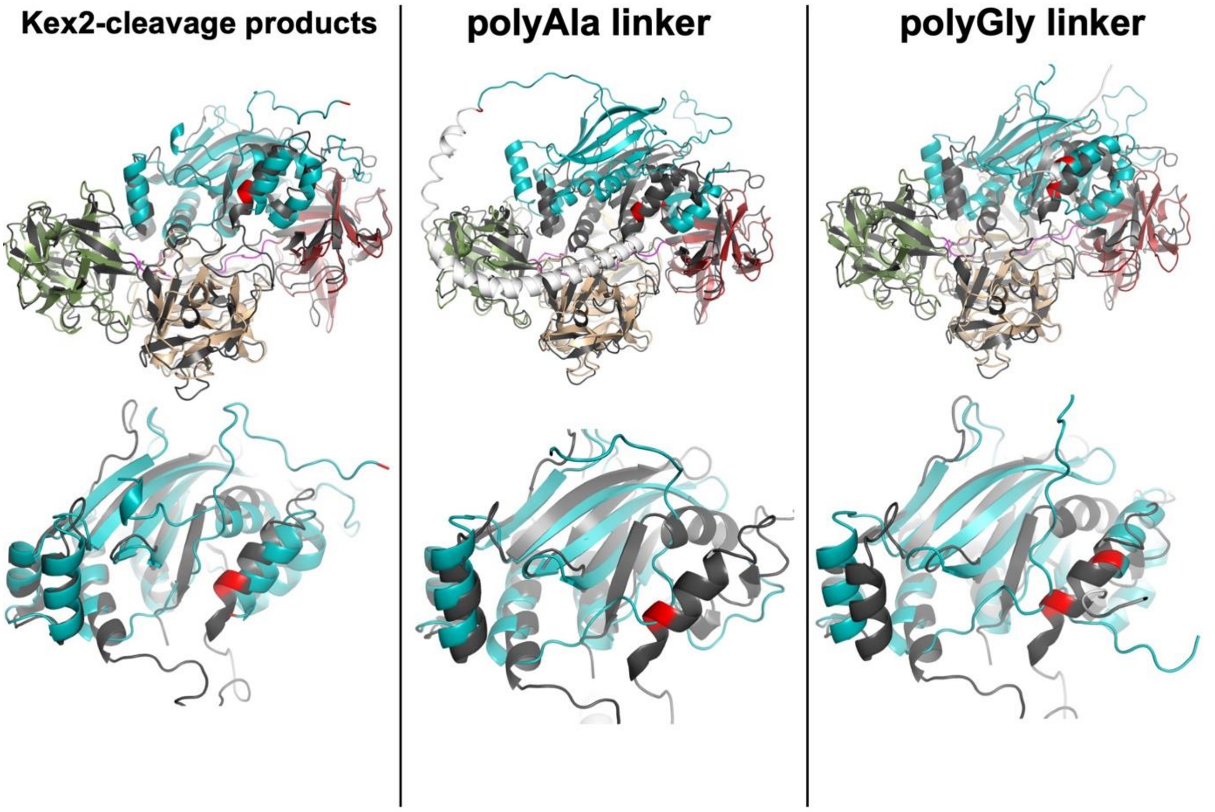

Figure for review only. *AlphaFold3 folding simulations run on the CCTX2 Kex2-cleavage products or on the contiguous full-length protein carrying a flexible insert at the putative kexin cleavage site (colored in red). In each of the three panels, the upper section shows the*

overlay of the cryo-EM structure of full-length CCTX2 protein (black) and the AlphaFold3 simulation of the respective CCTX2 variant (color); the lower section of each panel shows the respective overlay of the DUF domain only.

Dear Dr Künzler,

Thank you again for submitting your amended manuscript (EMBOJ-2025-121420R) to The EMBO Journal. Please accept my apologies for the protraction with the reassessment due to delayed expert input at this time of the year and detailed discussion in the editorial team. Your revised study was sent back to the referees for their scientific reassessment, and we have received detailed re-reports from both, which I attach below. As you will see, the referees state that the work has been substantially enhanced by the revisions and they are now in favour of publication, pending minor revision.

Thus, we are pleased to inform you that your manuscript has been accepted in principle for publication in The EMBO Journal.

Please carefully consider the remaining minor point still raised by referee #2 by adjusting the data processing information in the manuscript text where appropriate.

Also, we now need you to take care of a number of minor issues related to formatting and data annotation as detailed below, together with additional changes and requests by our production team for Source Data provision.

As you might have seen on our web page, every paper at the EMBO Journal now includes a 'Synopsis', displayed on the html and freely accessible to all readers. The synopsis includes a 'model' figure as well as 2-5 one-short-sentence bullet points that summarize the article. I would appreciate if you could provide this figure and the bullet points.

Please submit a revised version of the manuscript using the link enclosed below.

Thank you again for giving us the chance to consider your manuscript for The EMBO Journal. I look forward to receiving your final revised version of the manuscript fro swift acceptance and online publication.

Kind regards,

Daniel Klimmeck

Daniel Klimmeck PhD
Senior Editor
The EMBO Journal

****

Formatting changes required for the revised version of the manuscript:

>> Please add up to five keywords to your study.

>> Author Contributions: Remove the author contributions information from the manuscript text. Note that CRediT has replaced the traditional author contributions section as of now because it offers a systematic machine-readable author contributions format that allows for more effective research assessment. and use the free text boxes beneath each contributing author's name to add specific details on the author's contribution.

More information is available in our guide to authors.
https://link.springer.com/journal/44318/submission-guidelines

>> Author information: please resolve the name discrepancy between Ahmad A. Ahmad (manuscript) vs Ahmad Ali-Ahmad (online system).

>> Authors: All corresponding authors need accounts in our online system with their ORCID number linked (U.K.). Please see below for additional information.

>> Provide a 'Disclosure and Competing Interests Statement' and place after Acknowledgements.

>> 'MATERIALS AND METHODS' should be labeled as 'Methods'.

>> Section order should be as follows: title page with complete author information, abstract, keywords, introduction, results, discussion, methods, data availability section, acknowledgements, disclosure and competing interests statement, references, main figure legends, tables, expanded figure legends.´.

>> Figure callouts: please add a callout for a Appendix Table S10 in the main text.

>> Remove the 'data not shown' statement on p.7, or add respective information.

>> Funding: Please add the complete list of funders to our online system; currently ' Norwegian INFRASTRUKTUR-program (project no. 245828) as well as from UiO (core facility funds)' are missing; the list will be linked to the central database in PubMed in all published articles, it is therefore essential that the list in our system is complete and accurate.

>>Appendix file with ToC: needs to be in PDF, needs a Table of Contents on the title page with each item listed (figures, tables) with its page number, cover page should include Appendix for 'MS Title'; the nomenclature is Appendix Figure S1, etc. Appendix Table S1, etc.; callouts in manuscript to be adjusted - all figures/tables needs to be called out as Appendix Figure Sx and Appendix Table Sx

>> Add a separate 'Statistical Analysis' section to the Methods part, detailing the algorithms and statistical tests applied.

>> Data availability section: please make sure the PDB entry information is updated and datasets are made publicly accessible.

>> The 'Abbreviations' list needs to be removed from the manuscript. Abbreviations should be defined in brackets after their first mention in the text, not in a list of abbreviations.

Further information is available in our Guide For Authors: https://link.springer.com/journal/44318/submission-guidelines

Please use the link below to submit your revision:
https://emboj.msubmit.net/cgi-bin/main.plex

Referee #1:

The authors performed new experiments to answer my previous questions. They included the missing reference. The new version is fine for me

Referee #2:

Authors have done an excellent job addressing issues raised in review. I have only two minor comments.
I made an extensive comment on how to report magnification. While authors made some changes, I do not believe the way they

report these values is correct. Specifically, they state
"...magnification of 215,000 x and with a calibrated pixel size of 0.63 Å..."
This would mean that the physical pixel size of the detector is about 13.5 micron, which, to my knowledge, is not what Gatan K2 physical pixel size is. It is 5um instead, which means that the true value of the magnification is around 80,000x (assuming that data was not collected in super resolution mode).
I also commented on the somewhat odd downsampling of the particles that authors used. There is nothing wrong with that and no need to change anything. I would just like to point out that using a smaller pixel size available from raw data could still improve resolution, at least that is my personal observation on many occasions. Of course, authors are correct that such data reprocessing has no potential to alter any conclusions they reach based on the structure.

All editorial and formatting issues were resolved by the authors.

2nd Revision - Editorial Decision 5th May 2026

Dear Dr. Künzler,

Thank you for submitting the revised version of your manuscript. I have now evaluated your amended manuscript and concluded that the remaining minor concerns have been sufficiently addressed.

I am thus pleased to inform you that your manuscript has been accepted for publication in the EMBO Journal.

Your manuscript will be processed for publication by EMBO Press. It will be copy edited and you will receive page proofs prior to publication. Please note that you will be contacted by Springer Nature Author Services to complete licensing and payment information.

Please note that it is The EMBO Journal policy for the transcript of the editorial process (containing referee reports and your response letter) to be published as an online supplement to each paper. If you do NOT want this, you will need to inform the Editorial Office via email immediately. More information is available here: https://www.embopress.org/transparent-process#Review_Process

You may qualify for financial assistance for your publication charges - either via a Springer Nature fully open access agreement or an EMBO initiative. Check your eligibility: https://www.embopress.org/page/journal/14602075/authorguide#chargesguide

Should you be planning a Press Release on your article, please get in contact with embo_production@springernature.com as early as possible in order to coordinate publication and release dates.

If you have any questions, please do not hesitate to contact the Editorial Office. Thank you for your contribution to The EMBO Journal.

Thank you again for this contribution to The EMBO Journal and congratulations on a successful publication! Please consider us again in the future for your most exciting work.

Best regards,

Daniel Klimmeck

Daniel Klimmeck, PhD
Senior Editor
The EMBO Journal
EMBO
Postfach 1022-40
Meyerhofstrasse 1
D-69117 Heidelberg
contact@embojournal.org

*****
Please note that it is The EMBO Journal policy for the transcript of the editorial process (containing referee reports and your response letters) to be published as an online supplement to each paper. If you should prefer removal of any referee-only figures included in the point-by-point response(s), e.g. because they may still be used for future publication or because they have been reproduced from published work by others, please do let us know immediately via response email.
More information is available here: https://link.springer.com/partners/embo-press/editorial-policies#Peer%20review

** Click here to be directed to your login page: https://emboj.msubmit.net

EMBO Press Author Checklist

Corresponding Author Name: Ute Krengel and Markus Künzler
Journal Submitted to: The EMBO Journal
Manuscript Number: EMBOJ-2025-121420

USEFUL LINKS FOR COMPLETING THIS FORM
The EMBO Journal - Author Guidelines
EMBO Reports - Author Guidelines
Molecular Systems Biology - Author Guidelines
EMBO Molecular Medicine - Author Guidelines

Reporting Checklist for Life Science Articles (updated January
This checklist is adapted from Materials Design Analysis Reporting (MDAR) Checklist for Authors. MDAR establishes a minimum set of requirements in transparent reporting in the life sciences (see Statement of Task: 10.31222/osf.io/9sm4x). Please follow the journal's guidelines in preparing your
Please note that a copy of this checklist will be published alongside your article.

Abridged guidelines for figures
1. Data
The data shown in figures should satisfy the following conditions:
- the data were obtained and processed according to the field's best practice and are presented to reflect the results of the experiments in an accurate and unbiased manner.
- ideally, figure panels should include only measurements that are directly comparable to each other and obtained with the same assay.
- plots include clearly labeled error bars for independent experiments and sample sizes. Unless justified, error bars should not be shown for technical
- if n<5, the individual data points from each experiment should be plotted. Any statistical test employed should be justified.
- Source Data should be included to report the data underlying figures according to the guidelines set out in the authorship guidelines on Data

2. Captions
Each figure caption should contain the following information, for each panel where they are relevant:
- a specification of the experimental system investigated (eg cell line, species name).
- the assay(s) and method(s) used to carry out the reported observations and measurements.
- an explicit mention of the biological and chemical entity(ies) that are being measured.
- an explicit mention of the biological and chemical entity(ies) that are altered/varied/perturbed in a controlled manner.
- the exact sample size (n) for each experimental group/condition, given as a number, not a range;
- a description of the sample collection allowing the reader to understand whether the samples represent technical or biological replicates (including how many animals, litters, cultures, etc.).
- a statement of how many times the experiment shown was independently replicated in the laboratory.
- definitions of statistical methods and measures:
  - common tests, such as t-test (please specify whether paired vs. unpaired), simple χ2 tests, Wilcoxon and Mann-Whitney tests, can be unambiguously identified by name only, but more complex techniques should be described in the methods section;
  - are tests one-sided or two-sided?
  - are there adjustments for multiple comparisons?
  - exact statistical test results, e.g., P values = x but not P values < x;
  - definition of 'center values' as median or average;
  - definition of error bars as s.d. or s.e.m.

Please complete ALL of the questions below.
Select "Not Applicable" only when the requested information is not relevant for your study.

Materials

Newly Created Materials	Information included in the manuscript?	In which section is the information available? (Reagents and Tools Table, Materials and Methods, Figures, Data Availability Section)
New materials and reagents need to be available; do any restrictions apply?	Yes	Appendix Table S9

Antibodies	Information included in the manuscript?	In which section is the information available? (Reagents and Tools Table, Materials and Methods, Figures, Data Availability Section)
For **antibodies** provide the following information:<br>- Commercial antibodies: RRID (if possible) or supplier name, catalogue number and or/clone number<br>- Non-commercial: RRID or citation	Yes	Materials and Methods

DNA and RNA sequences	Information included in the manuscript?	In which section is the information available? (Reagents and Tools Table, Materials and Methods, Figures, Data Availability Section)
**Short novel DNA or RNA including primers, probes**: provide the sequences.	Yes	Appendix Table S11

Cell materials	Information included in the manuscript?	In which section is the information available? (Reagents and Tools Table, Materials and Methods, Figures, Data Availability Section)
**Cell lines:** Provide species information, strain. Provide accession number in repository **OR** supplier name, catalog number, clone number, and/**OR** RRID.	Not Applicable	
**Primary cultures:** Provide species, strain, sex of origin, genetic modification status.	Not Applicable	
Report if the cell lines were recently **authenticated** (e.g., by STR profiling) and tested for mycoplasma contamination.	Not Applicable	

Experimental animals	Information included in the manuscript?	In which section is the information available? (Reagents and Tools Table, Materials and Methods, Figures, Data Availability Section)
**Laboratory animals or Model organisms:** Provide species, strain, sex, age, genetic modification status. Provide accession number in repository **OR** supplier name, catalog number, clone number, **OR** RRID.	Yes	Appendix Table S10
**Animal observed in or captured from the field:** Provide species, sex, and age where possible.	Not Applicable	
Please detail **housing and husbandry conditions**.	Not Applicable	

Plants and microbes	Information included in the manuscript?	In which section is the information available? (Reagents and Tools Table, Materials and Methods, Figures, Data Availability Section)
**Plants:** provide species and strain, ecotype and cultivar where relevant, unique accession number if available, and source (including location for collected wild specimens).	Not Applicable	
**Microbes:** provide species and strain, unique accession number if available, and source.	Yes	Appendix Table S10

Human research participants	Information included in the manuscript?	In which section is the information available? (Reagents and Tools Table, Materials and Methods, Figures, Data Availability Section)
If collected and within the bounds of privacy constraints report on age, sex and gender or ethnicity for all study participants.	Not Applicable	

Core facilities	Information included in the manuscript?	In which section is the information available? (Reagents and Tools Table, Materials and Methods, Figures, Data Availability Section)
If your work benefited from core facilities, was their service mentioned in the acknowledgments section?	Yes	Acknowledgments

Design

Study protocol	Information included in the manuscript?	In which section is the information available? (Reagents and Tools Table, Materials and Methods, Figures, Data Availability Section)
If study protocol has been **pre-registered, provide DOI in the manuscript**. For clinical trials, provide the trial registration number **OR** cite DOI.	Not Applicable	
Report the **clinical trial registration number** (at ClinicalTrials.gov or equivalent), where applicable.	Not Applicable	

Laboratory protocol	Information included in the manuscript?	In which section is the information available? (Reagents and Tools Table, Materials and Methods, Figures, Data Availability Section)
Provide DOI OR other citation details if **external detailed step-by-step protocols** are available.	Not Applicable	

Experimental study design and statistics	Information included in the manuscript?	In which section is the information available? (Reagents and Tools Table, Materials and Methods, Figures, Data Availability Section)
Include a statement about **sample size** estimate even if no statistical methods were used.	Not Applicable	
Were any steps taken to minimize the effects of subjective bias when allocating animals/samples to treatment (e.g. **randomization procedure**)? If yes, have they been described?	Not Applicable	
Include a statement about **blinding** even if no blinding was done.	Not Applicable	
Describe **inclusion/exclusion criteria** if samples or animals were excluded from the analysis. Were the criteria pre-established?<br><br>If sample or data points were omitted from analysis, report if this was due to attrition or intentional exclusion and provide justification.	Not Applicable	
For every figure, are **statistical tests** justified as appropriate? Do the data meet the assumptions of the tests (e.g., normal distribution)? Describe any methods used to assess it. Is there an estimate of variation within each group of data? Is the variance similar between the groups that are being statistically compared?	Yes	Figure Legends

Sample definition and in-laboratory replication	Information included in the manuscript?	In which section is the information available? (Reagents and Tools Table, Materials and Methods, Figures, Data Availability Section)
In the figure legends: state number of times the experiment was **replicated** in laboratory.	Yes	Figure Legends
In the figure legends: define whether data describe **technical or biological replicates**.	Yes	Figure Legends

Ethics

Ethics	Information included in the manuscript?	In which section is the information available? (Reagents and Tools Table, Materials and Methods, Figures, Data Availability Section)
Studies involving **human participants**: State details of **authority granting ethics approval** (IRB or equivalent committee(s), provide reference number for approval.	Not Applicable	
Studies involving **human participants**: Include a statement confirming that **informed consent** was obtained from all subjects and that the experiments conformed to the principles set out in the WMA Declaration of Helsinki and the Department of Health and Human Services Belmont Report.	Not Applicable	
Studies involving **human participants:** For publication of **patient photos**, include a statement confirming that consent to publish was obtained.	Not Applicable	
Studies involving experimental **animals**: State details of **authority granting ethics approval** (IRB or equivalent committee(s), provide reference number for approval. Include a statement of compliance with ethical regulations.	Not Applicable	
Studies involving **specimen and field samples:** State if relevant **permits** obtained, provide details of authority approving study; if none were required, explain why.	Not Applicable	

Dual Use Research of Concern (DURC)	Information included in the manuscript?	In which section is the information available? (Reagents and Tools Table, Materials and Methods, Figures, Data Availability Section)
Could your study fall under dual use research restrictions? Please check biosecurity documents and list of **select agents and toxins** (CDC): https://www.selectagents.gov/sat/list.htm	Not Applicable	
If you used a select agent, is the security level of the lab appropriate and reported in the manuscript?	Not Applicable	
If a study is subject to dual use research of concern regulations, is the name of the **authority granting approval and reference number** for the regulatory approval provided in the manuscript?	Not Applicable	

Reporting

The MDAR framework recommends adoption of discipline-specific guidelines, established and endorsed through community initiatives. Journals have their own policy about requiring specific guidelines and recommendations to complement MDAR.

Adherence to community standards	Information included in the manuscript?	In which section is the information available? (Reagents and Tools Table, Materials and Methods, Figures, Data Availability Section)
State if relevant guidelines or checklists (e.g., **ICMJE, MIBBI, ARRIVE, PRISMA**) have been followed or provided.	Not Applicable	
For **tumor marker prognostic studies,** we recommend that you follow the **REMARK** reporting guidelines (see link list at top right). See author guidelines, under 'Reporting Guidelines'. Please confirm you have followed these guidelines.	Not Applicable	
For **phase II and III randomized controlled trials**, please refer to the **CONSORT** flow diagram (see link list at top right) and submit the CONSORT checklist (see link list at top right) with your submission. See author guidelines, under 'Reporting Guidelines'. Please confirm you have submitted this list.	Not Applicable	

Data Availability

Data availability	Information included in the manuscript?	In which section is the information available? (Reagents and Tools Table, Materials and Methods, Figures, Data Availability Section)
Have **primary datasets** been deposited according to the journal's guidelines (see 'Data Deposition' section) and the respective accession numbers provided in the Data Availability Section?	Yes	Data Availability Section
Were **human clinical and genomic datasets** deposited in a public access-controlled repository in accordance to ethical obligations to the patients and to the applicable consent agreement?	Not Applicable	
Are **computational models** that are central and integral to a study available without restrictions in a machine-readable form? Were the relevant accession numbers or links provided?	Not Applicable	
If publicly available data were reused, provide the respective **data citations in the reference list**.	Yes	Reference List
